# Efficient synthesis of *O*-glycosylated amino acids†

**DOI:** 10.1039/d5cb00076a

**Published:** 2025-05-07

**Authors:** Felicity J. Frank, Rebecca A. Lawson, Tom E. McAllister

**Affiliations:** a School of Natural and Environmental Science, Newcastle University Newcastle upon Tyne NE1 7RU UK tom.mcallister@newcastle.ac.uk

## Abstract

Protein glycosylation is one of the most abundant and complex post-translational modifications, necessitating many different approaches to fully understand the biological effects. Investigation using synthetic glycopeptides is limited by the high cost of building blocks; typically >100*x* more than other modified amino acids *e.g.* phosphorylation. We report a simple, low cost route to *O*-glycosylated amino acids suitable for Fmoc-SPPS in two or three steps starting from peracetylated sugars. One set of reagents can furnish either the α- or β-anomer through adjusting the equivalents and reaction time. Depending on the derivative, the cost of our route is 25–60× less than commercial alternatives and offers scope for producing modified analogues. Overall, this is a convenient and user-friendly approach to access *O*-glycosylated amino acids, urgently required for continued investigation of the manifold roles of glycosylation in biology.

## Abstract 2

Synthetic glycopeptides are versatile tools to investigate protein glycosylation. In this work, we outline a short, economical synthesis to *O*-glycosylated amino acids compatible with Fmoc-SPPS.

## Graphical abstract

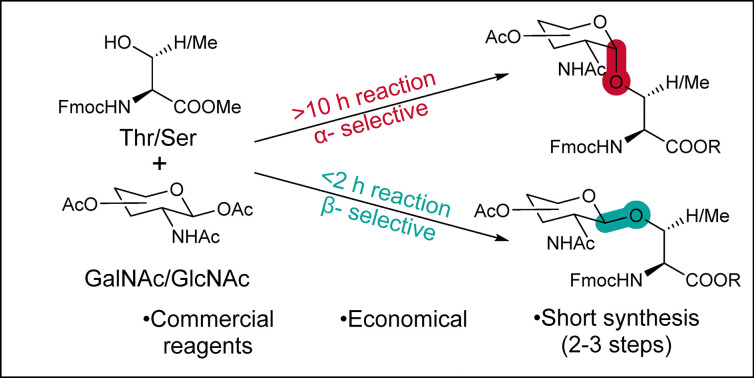

Protein glycosylation is an abundant post-translational modification,^1^ with glycans linked to specific amino acid side chains and categorised by the linking atom with *C*-, *O*-, *N*- and *S*-glycans all observed.^2^ The *O*-glycans are the most diverse with the covalent attachment occurring through serine (Ser) and threonine (Thr) (as well as reports of tyrosine, Tyr) sidechains. They are subdivided by the identity of initial monosaccharide, which can be glucose (Glc), galactose (Gal), mannose (Man), fucose (Fuc), xylose (Xyl), *N*-acetylglucosamine (GlcNAc) or *N*-acetylgalactosamine (GalNAc).^2^ Canonical α-*O*-GalNAc glycosylation is the most abundant and complex form, occurring on secreted and membrane proteins in dense clusters in mucin domains forming the principal component of the mucosal layer in the gut epithelium and providing physical protection from bacteria.^3^ Additionally, α-*O*-GalNAc glycans occur on non-mucin proteins and are involved in cellular communication, regulation of protein half-life and host pathogen interactions,^4^ as well as co-occurrence with other *O*-glycan types such as *O*-mannose, observed in α-dystroglycan.^5,6^ Among the best characterised of these specific modifications is the fibroblast growth factor (FGF) 23 α-*O*-GalNAc modification at Thr178 introduced by ppGalNAcT3, regulating FGF23 secretion and thus calcium homeostasis.^7,8^ The apparent dichotomy between both generic and specific functions is one of the pressing areas of research for *O*-GalNAc glycans; there are likely further specific examples yet to be identified.^9^*O*-GalNAc glycans are also implicated in disease pathology; dysregulation of the GalNAc transferases and production of truncated glycans is associated with various diseases including cancer.^10–12^

A common approach to studying these modifications is through production of synthetic glycopeptides *e.g.* for *in vitro* enzyme reactions,^7,13,14^ structural biology^15,16^ or vaccines.^17,18^ While some *O*-GalNAc glycosylated amino acid building blocks for Fmoc-SPPS are commercially available, they are typically very expensive. From a survey of UK online prices, they are >7000× more expensive on a molar basis than their non-glycosylated counterparts and >100× more than derivatives of other common modifications *e.g.* phosphorylation (Table S1, ESI†). Approaches have thus been developed to minimise the quantities needed but this is not feasible for all applications.^19^ The high costs are a barrier to progress and particularly likely to deter non-specialist researchers from venturing into the area. Chemical synthesis requires formation of the glycosidic bond and many methods exist, though the low reactivity of oxygen nucleophiles makes *O*-glycosylation more challenging. More generally, a major challenge in carbohydrate chemistry is achieving selective formation of 1,2-*cis*-glycosides when C2 bears an ester or amide (α-stereochemistry in gluco- and galacto-configured pyranoses), as neighbouring-group participation (NGP) from C2 typically directs glycosylations to the β-(1,2-*trans*) configuration.^20^ This is not always the case and there appear to be more subtleties and nuances to this process than previously realised.^21^ There are many existing syntheses (>30) to Fmoc-Thr[GalNAc(Ac)_3_-α-d]-OH α1, recently reviewed by Liu *et al.*,^22^ but on average these require at least seven steps from commercial reagents and have varying degrees of control over the anomer formed.

As part of our ongoing work to investigate protein *O*-GalNAc glycosylation, we set out to synthesise the building blocks Fmoc-Thr[GalNAc(Ac)_3_-α-d]-OH α1 and Fmoc-Ser[GalNAc(Ac)_3_-α-d]-OH α2 (Fig. 1).

**Fig. 1:**
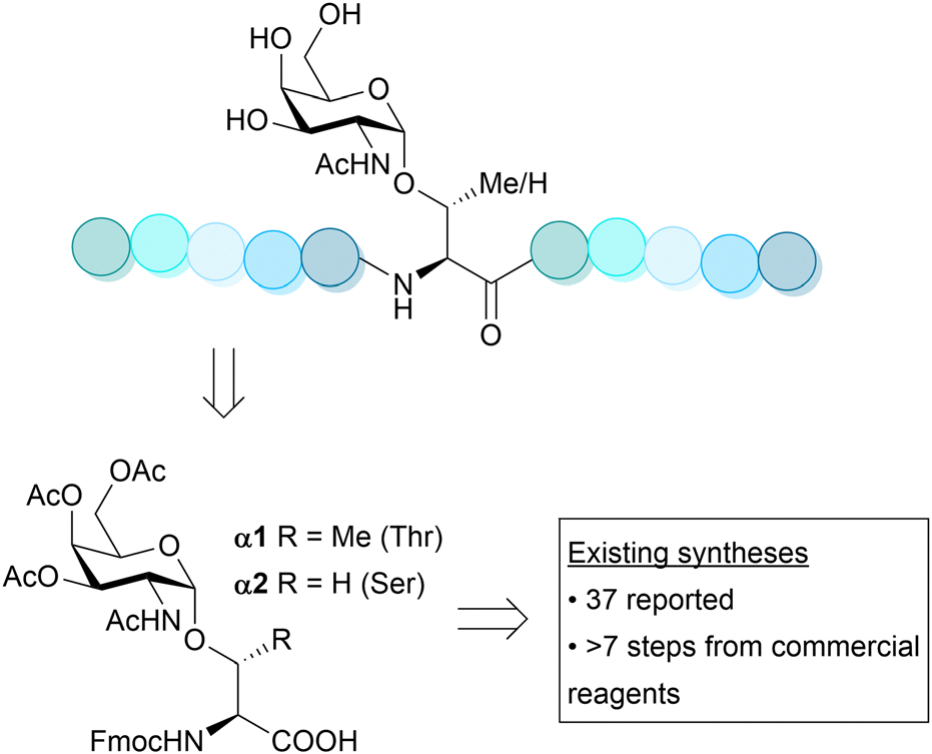
Glycopeptides are useful reagents for understanding protein glycosylation but require compatible building blocks for Fmoc-SPPS. Reported syntheses of α1 were recently reviewed by Liu *et al.*^22^

We initially deployed the Ni-catalysed synthesis reported by Yu *et al.*^23^ using a C2 imine to overcome NGP but found preparation of the appropriate donor lengthy and the glycosylation inconsistent in our hands. Eager to pursue a shorter synthesis, we investigated the ferric chloride-catalysed reaction reported by Wei *et al.*^24^ using commercially available *N*-acetylgalactosamine tetraacetate β-GalNAc(Ac_4_) β3 and Fmoc-Ser-OMe 4 in refluxing 1,2-dichloroethane (DCE) (see Table 1 for structures). Using this method, we could only produce small quantities of the β-anomer as product. A similar procedure was more recently described by Sommer *et al.*^25^ using copper(ii) triflate (Cu(OTf)_2_) to catalyse glycosylation of simple alcohols with *N*-acetylglucosamine tetraacetate (β-GlcNAc(Ac_4_)) β5 (see Table 1 for structure) in refluxing DCE. This reaction was shown to be stereodivergent as either the α- or β-anomers could be produced using the same reagents but under different reaction conditions; shorter reaction times giving predominantly β-product with more α-product from prolonged reaction times. In this paper we detail our work to explore the scope of this reaction for producing either anomer for both GalNAc and GlcNAc glycosylated threonine and serine amino acids, to generate building blocks suitable for Fmoc-SPPS.

**Table 1:** Exploration of glycosylation reaction

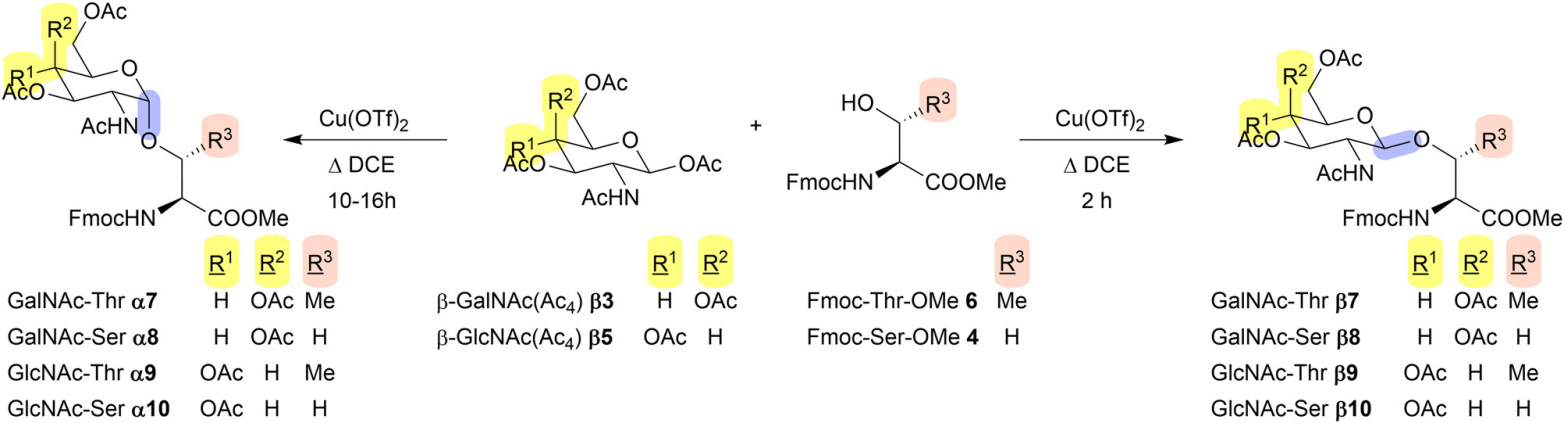						
Entry^a^	Donor	Acceptor	Equiv. acceptor	Time/h	Product (yield/%)	*α* : *β* ratio
1	β3	6	5	1.6	β7 (82)	β selective^f^
2	β3	4	5	1.6	β8 (66)	β selective^f^
3	β5	6	5	1.6	β9 (57)	β selective^f^
4	β5	4	5	1.6	β10 (68)	β selective^f^
5	β3	4	5	16	β8 + α8 (68)^b^	1 : 1
6	β3	4	2	10	β8 + α8 (34)^b^	3 : 1
7	β3	4	1	10	α8 (12)	α selective^f^
8	β3	4^c^	0.2	10	β8 + α8^e^	2 : 1
9	β3	4	0.2	10	α8^e^	α selective^f^
10	β5	4	1	10	β10 (19)	β selective^f^
11	β5	4	1	24	β10 + α10 (9)^b^	2 : 1
12	β3	6	5	16	α7 (39), β7 (20)	1.9 : 1
13	β5	6	5	16	α9 (10), β9 (29)	1 : 2.7
14	β5	6	1	16	α9(6), β9(7)	1 : 1.2
15^d^	β3	6	5	16	α7 (32)	N.D.
16^d^	β3	6	5	1.6	β7 (75)	N.D.
17^g^	β3	4	1	10	α8 (12)	N.D.
18^d^	β3	4	5	1.6	β8 (95)	N.D.

a Unless otherwise stated all reactions were performed with 258 μmol of donor, 1 equiv. of promoter Cu(OTf)_2_ and 5 equiv. of acceptor (equiv. relative to donor) at 51.6 mM [donor] in refluxing DCE.

b α and β products were inseparable *via* silica gel column chromatography, ratio determined by ^1^H NMR.

c 0.2 equiv. of Cu(OTf)_2_ was used in this reaction.

d Reactions were performed on 5.15 mmol.

e Yield too low to recover, product identified by LCMS.

f None of the other anomer was observed.

g Reaction was performed on 774 μmol; N. D. – not determined.

While primarily targeting GalNAc-modified amino acids, the capacity to also use GlcNAc donors makes the reaction more versatile and the resulting glycosylated amino acids could have applications in dissecting the effects of the glycosidic linkage stereochemistry in biological settings. Furthermore, β-*O*-GlcNAc addition to serine and threonine is an abundant dynamic modification of intracellular proteins^26^ and while β-*O*-GalNAc and α-*O*-GlcNAc are not known protein modifications in humans, the potential for UPD-GlcNAc to be used by human GalNAc transferases (generating Ser/Thr-α-*O*-GlcNAc) has been shown,^27^ and in other species such as trypanosomes, GlcNAc is used exclusively in place of GalNAc in mucin-type glycans.^28^

As aforementioned, formation of β-*O*-GlcNAc/GalNAc typically predominates through NGP and routes to these Fmoc-amino acids are relatively straightforward,^29–31^ but facile access to all derivatives with the same reagents would be a timely advance to facilitate further study of protein *O*-glycosylation. More divergent approaches have gained significant interest in recent years with the development of bimodal donors capable of yielding either anomer by use of different activation conditions.^32^ Furthermore, in the absence of NGP the choice of solvent or additives can lead to different glycosylation selectivities,^33,34^ though we could not find many precedents applicable to our proposed conditions. Using peracetylated glucose (*i.e.* with OAc at C2 so likely to undergo NGP) and serine derived acceptors under reflux conditions, Lefever *et al.* showed reactions in DCE to give higher proportions of α-product than in toluene, a less polar solvent.^30^ Based on these findings and the work from Sommer *et al.*^25^ we restricted our studies to DCE as reaction solvent.

We initially used commercially available Fmoc-Thr-OH with β-GalNAc(Ac_4_) β3 and Cu(OTf)_2_ in refluxing DCE and while we did observe formation of new products, separation from unreacted acceptor was laborious and anomers proved impossible to resolve (data not shown). Hence, we elected to use esters of the corresponding amino acids. We initially considered commonly used orthogonal esters such as *t*Bu and allyl, but the *t*Bu is unlikely to survive the high temperatures and acidic conditions (Cu(OTf)_2_ has been previously used as a deprotection agent for *t*Bu groups on amines).^35^ We initially prepared allyl esters, which functioned as acceptors in reactions with β-GalNAc(Ac_4_) β3, but purification was hampered by formation of additional products (Fig. S1, ESI†). Further investigation suggested other modifications to the amino acid side chain hydroxyl, including possible migration of the allyl group (Fig. S1, ESI†). Thus, we focussed our efforts on methyl esters as they can also be orthogonally removed post glycosylation.^36^ Fmoc-Ser-OMe 4 is commercially available while Fmoc-Thr-OMe 6 was synthesised from the corresponding methyl ester hydrochloride salt quantitatively (details in ESI†).

Initial results with the methyl esters were very encouraging; glycosylation of Fmoc-Thr-OMe 6 (5 equiv.) with β-GalNAc(Ac_4_) β3 in the presence of Cu(OTf)_2_ (1 equiv.) for 1.6 h yielded the β-product Fmoc-Thr[GalNAc(Ac)_3_-β-d]-OMe β7 in 82% isolated yield (Table 1, entry 1). Likewise, the corresponding reaction with serine acceptor 4 gave 66% isolated yield of Fmoc-Ser[GalNAc(Ac)_3_-β-d]-OMe β8 (Table 1, entry 2) and in both cases we could also recover most of the unreacted acceptor during purification (69% and 68% respectively).‡‡The maximum amount that could be recovered is 80%. The quoted product yields are based on donor input so full conversion of donor to product would result in 80% unreacted acceptor as the acceptor is used in 5-fold excess. The corresponding reaction with β-GlcNAc(Ac_4_) β5 gave similar yields for Fmoc-Thr[GlcNAc(Ac)_3_-β-d]-OMe β9 and Fmoc-Ser[GlcNAc(Ac)_3_-β-d]-OMe β10 (Table 1, entries 3 and 4).

Following the successful isolation of β7–β10, our investigation turned to the synthesis of the equivalent α isomers. Initially, β3 was reacted with 5 equiv. 4 for 16 hours, showing the formation of one major product by TLC. However, after isolation of this compound, it was determined that both products Fmoc-Ser[GalNAc(Ac)_3_-α-d]-OMe α8 and β8 have identical Rf values, resulting in an inseparable 1 : 1 ratio of the two products (Table 1, entry 5). Therefore, a series of screening reactions were carried out to selectively produce α8. First, we reduced the equivalents of acceptor 4 to 3.5 and 2 equiv. relative to β3, while maintaining the 1 equiv. of Cu(OTf)_2_. After 16 hours reflux in DCE, both reactions showed only α8 as determined by LCMS. However, the yield was so low that α8 could not be isolated. Interestingly, the LCMS also showed a major peak consistent (based on observed mass) with Fmoc-Ser(Ac)-OMe 11 (Fig. S2, ESI†). Acetate is liberated from the donor and is presumably able to compete for reaction with the acceptor, which is in excess. Close inspection of previous LCMS reaction monitoring data also showed varying (minor) amounts of acetylated amino acid byproduct in reactions with Thr acceptor 6 as well (Fig. S3, ESI†). We postulated that a shorter reaction time may limit this side reaction occurring, therefore β3 was reacted with 2 equiv. of 4 for 10 hours. Isolation of α8 and β8 in a 3 : 1 ratio (calculated by NMR) was observed, suggesting that the shorter reaction time did indeed limit the competing acetylation (Table 1, entry 6). Further reduction in acceptor 4 to 1 equiv. enhanced α selectivity, resulting in 12% isolated yield of α8 (Table 1, entry 7) without any co-production of β8. Although this was an excellent result for selectivity, a reduction in yield was observed, therefore a final screening reaction was carried out using 5 equiv. β3 and 1 equiv. 4 to increase yields of β3 further (Table 1, entry 8). Unfortunately, although LCMS indicated the formation α8, only trace amounts were formed that could not be isolated with a major product of Fmoc-Ser(Ac)-OMe 11.

After the successful synthesis of α8, these same optimised reaction conditions were utilised, using β5 as an alternative donor (Table 1, entry 10). After 10 h, only β10 was isolated, indicating the formation of α10 is significantly slower than α8. The lower reactivity of glucopyranosyl donors relative to galactopyranosyl donors has been reported previously^37^ though the apparent complete lack of α10 was unexpected. Extension of the reaction time to 24 hours resulted in a 9% combined yield of α10 and β10 in a 2 : 1 ratio, calculated by NMR (Table 1, entry 11). Despite further attempts we could not synthesise α10 without co-production of β10 and since we were unable to separate them during purification we conclude that α10 cannot be produced directly by this method in our hands.

Next, we investigated the corresponding threonine products. Donor β3 was refluxed with 5 equiv. of 6 for 16 hours, resulting in the formation of a mixture with 39% yield of Fmoc-Thr[GalNAc(Ac)_3_-α-d]-OMe α7, 20% yield β7 and recovery of 47% unreacted 6,^+^ with the anomers proving easily separable by silica gel column chromatography (Table 1, entry 12). Further, β5 was reacted with 5 equiv. of 6 for 16 h resulting in 10% and 29% isolated yields of Fmoc-Thr[GlcNAc(Ac)_3_-α-d]-OMe α9 and β9 respectively (Table 1, entry 13). This again indicates that GlcNAc donor β5 reacts slower than its GalNAc counterpart β3. Finally, β5 was reacted with 1 equiv. 6, reasoning this may increase the ratio of α9 (Table 1, entry 14) as was seen previously for α10. Although this resulted in less β9, the yield of the desired α-anomer α9 had also decreased, therefore it was determined that 5 equiv. of acceptor 6 was more suitable in this case as the α- and β-products are separable on silica. To explore the scalability of the reaction, we performed glycosylations with the previously optimised conditions on up to 5 mmol-scale (2 g of donor β3) to generate α7, β7, α8 and β8 in comparable yields to previously (entries 15–18).

While demonstrating that both α- and β-anomers can be produced using this method, yields for the β-anomer were significantly higher (up to 95%; Table 1, entry 18). Sommer *et al.* reported that β-GlcNAc glycosides could be anomerised to the corresponding α-glycosides by refluxing in DCE with 0.05 equiv. of Cu(OTf)_2_ and 1 equiv. HOAc.^25^ We attempted anomerization of β7 using identical conditions, however conversion to α7 was not observed, showing only degradation to unidentifiable compounds. We subsequently investigated further using a sample containing both α7 and β7 (1 : 7), with 1 equiv. of Cu(OTf)_2_ and monitored *via* LCMS with UV detection at 280 nm (Fig. 2). Over the first 5 hours we observed disappearance of β7 with concomitant generation of amino acid 6; apparently driving the reaction backwards to the starting materials.§§A new peak for the GalNAc portion of the molecule was also observed by MS but gives no UV signal so could not be quantified; data not shown. In contrast, the amount of α7 was unchanged throughout this period, but upon prolonged reaction times (23 h) the proportion of α7 did increase, suggesting this anomerisation is possible but we also observed formation additional products to 6, α7 or β7. Previously, Frem *et al.* observed no anomerization of GlcNAc-Ser β10 to α10 with Cu(OTf)_2_ in DCE under microwave conditions, though yield of β10 was lower than other substrates (30%), which could be due to side reactions.^38^ Our data suggest that for GalNAc-derivatives anomerisation proceeds *via* cleavage of the exocyclic bond in the β-glycoside, as has been previously proposed by Ikemoto *et al.*^39^ Further evidence came from immediate quenching of a sample from the reaction mixture with excess methanol, which showed formation of the methyl-galactoside (from LCMS data; Fig. S4, ESI†), whose formation is only possible if the exocyclic bond is broken. We did not investigate the anomerisation reaction further, though future work could focus on different Lewis acids.^38,40^

**Fig. 2:**
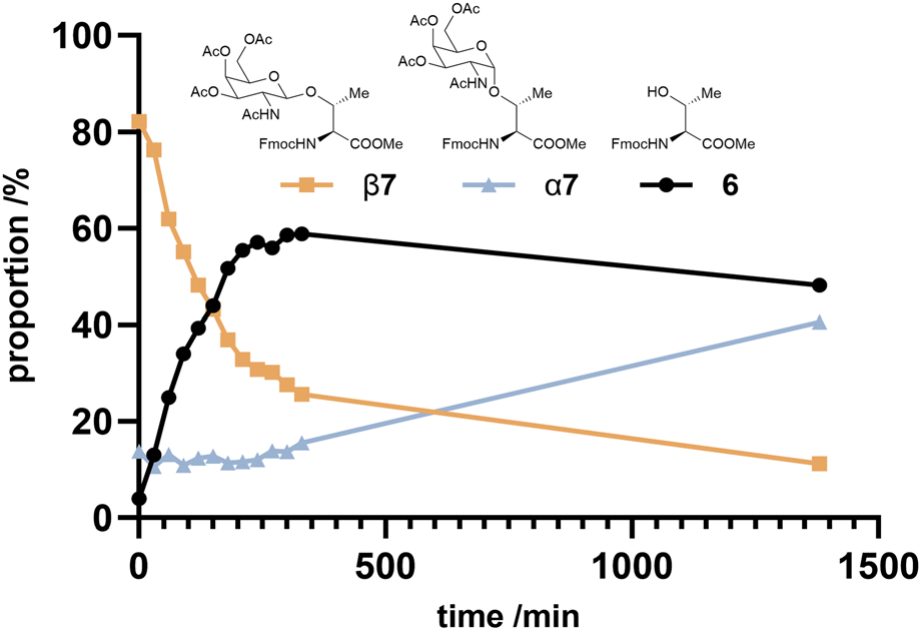
Product distribution over time following treatment of a 7 : 1 β7/α7 mixture to 1 equiv. Cu(OTf)_2_ in refluxing DCE as determined by LCMS (UV absorbance at 280 nm).

Finally, to furnish building blocks suitable for Fmoc-SPPS the methyl esters were removed using the LiI conditions reported by Mayato and coworkers.^36^ Aqueous workup yielded the corresponding amino acids without the need for additional purification, showing selective demethylation in up to 85% yield (Table 2). Overall, Fmoc-SPPS compatible glycosylated amino acids can be produced in up to 68% yield from commercial materials. Yields are variable between different products, but this route is significantly more economical than commercially purchased products, using cheap readily available materials with only one chromatographic purification required.

**Table 2:** Selective methyl ester removal

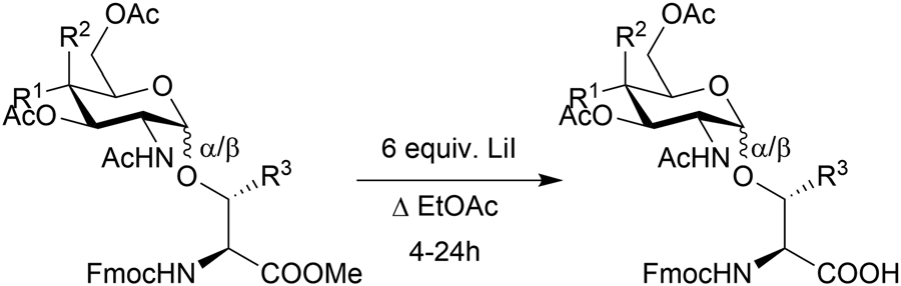							
Entry	*R* ^1^	*R* ^2^	*R* ^3^	α/β	Starting material	Product	Yield/%
1	H	OAc	Me	α	α7	α1	44
2	H	OAc	Me	β	β7	β1	84
3	H	OAc	H	α	α8	α2	61
4	H	OAc	H	β	β8	β2	85
5	OAc	H	Me	β	β9	β12	58
6	OAc	H	H	β	β10	β13	66

For comparison, we calculated the cost of synthesising 100 mg of both Fmoc-Thr[GalNAc(Ac)3-α-d]-OH α1 and Fmoc-Ser[GalNAc(Ac)3-α-d]-OH α2 using methods outlined above as being £6.11 and £7.64 respectively. This is 1.5% and 3.5% the price of the cheapest commercial options we could find (based on list prices – see ESI,† for details)¶¶Our price includes all reagents and reaction solvents but not labour, solvent used for workup and purification or potential reductions in costs through recovery of unreacted acceptor.. Furthermore, the β-anomers of both GalNAc and GlcNAc on serine and threonine can also be produced cheaply (£1.83–£3.93, per 100 mg), demonstrating access to a range of building blocks.

In conclusion, we present a simple two- or three-step synthesis (for Ser and Thr derivatives respectively) to make glycosylated Fmoc-SPPS compatible building blocks. We are hopeful that this new route will make glycopeptide synthesis more accessible/affordable and enable further advancements in the understanding of the myriad roles of protein glycosylation.

## Author contributions

FJF conceptualization, methodology, investigation, formal analysis, data curation, visualisation, writing – original draft, writing – review & editing. RAL methodology, investigation, formal analysis, data curation. TEM conceptualization, methodology, writing – original draft, writing – review & editing, visualisation, supervision, project administration, funding acquisition.

## Data availability

The data supporting this article have been included as part of the ESI.†

## Conflicts of interest

There are no conflicts to declare.

## Supplementary Material

CB-OLF-D5CB00076A-s001

## References

[cit1] An H. J., Froehlich J. W., Lebrilla C. B. (2009). Curr. Opin. Chem. Biol..

[cit2] Spiro R. G. (2002). Glycobiology.

[cit3] Lang T., Hansson G. C., Samuelsson T. (2007). Proc. Natl. Acad. Sci. U. S. A..

[cit4] Wandall H. H., Nielsen M. A. I., King-Smith S., de Haan N., Bagdonaite I. (2021). FEBS J..

[cit5] Tran D. T., Lim J.-M., Liu M., Stalnaker S. H., Wells L., Ten Hagen K. G., Live D. (2012). J. Biol. Chem..

[cit6] Borgert A., Foley B. L., Live D. (2021). Glycobiology.

[cit7] Rivas M., Paul Daniel E. J., Narimatsu Y., Compañón I., Kato K., Hermosilla P., Thureau A., Ceballos-Laita L., Coelho H., Bernadó P., Marcelo F., Hansen L., Maeda R., Lostao A., Corzana F., Clausen H., Gerken T. A., Hurtado-Guerrero R. (2020). Nat. Chem. Biol..

[cit8] Kato K., Jeanneau C., Tarp M. A., Benet-Pagès A., Lorenz-Depiereux B., Bennett E. P., Mandel U., Strom T. M., Clausen H. (2006). J. Biol. Chem..

[cit9] de Las Rivas M., Lira-Navarrete E., Gerken T. A., Hurtado-Guerrero R. (2019). Curr. Opin. Struct. Biol..

[cit10] Scott E., Hodgson K., Calle B., Turner H., Cheung K., Bermudez A., Marques F. J. G., Pye H., Yo E. C., Islam K., Oo H. Z., McClurg U. L., Wilson L., Thomas H., Frame F. M., Orozco-Moreno M., Bastian K., Arredondo H. M., Roustan C., Gray M. A., Kelly L., Tolson A., Mellor E., Hysenaj G., Goode E. A., Garnham R., Duxfield A., Heavey S., Stopka-Farooqui U., Haider A., Freeman A., Singh S., Johnston E. W., Punwani S., Knight B., McCullagh P., McGrath J., Crundwell M., Harries L., Bogdan D., Westaby D., Fowler G., Flohr P., Yuan W., Sharp A., de Bono J., Maitland N. J., Wisnovsky S., Bertozzi C. R., Heer R., Guerrero R. H., Daugaard M., Leivo J., Whitaker H., Pitteri S., Wang N., Elliott D. J., Schumann B., Munkley J. (2023). Oncogene.

[cit11] Julien S., Videira P. A., Delannoy P. (2012). Biomolecules.

[cit12] Ju T., Otto V. I., Cummings R. D. (2011). Angew. Chem., Int. Ed..

[cit13] Collette A. M., Hassan S. A., Schmidt S. I., Lara A. J., Yang W., Samara N. L. (2024). Sci. Adv..

[cit14] Pratt M. R., Hang H. C., Ten Hagen K. G., Rarick J., Gerken T. A., Tabak L. A., Bertozzi C. R. (2004). Chem. Biol..

[cit15] De Las Rivas M., Paul Daniel E. J., Coelho H., Lira-Navarrete E., Raich L., Compañón I., Diniz A., Lagartera L., Jiménez-Barbero J., Clausen H., Rovira C., Marcelo F., Corzana F., Gerken T. A., Hurtado-Guerrero R. (2018). ACS Cent. Sci..

[cit16] González-Ramírez A. M., Grosso A. S., Yang Z., Compañón I., Coelho H., Narimatsu Y., Clausen H., Marcelo F., Corzana F., Hurtado-Guerrero R. (2022). Nat. Commun..

[cit17] Bermejo I. A., Guerreiro A., Eguskiza A., Martínez-Sáez N., Lazaris F. S., Asín A., Somovilla V. J., Compañón I., Raju T. K., Tadic S., Garrido P., García-Sanmartín J., Mangini V., Grosso A. S., Marcelo F., Avenoza A., Busto J. H., García-Martín F., Hurtado-Guerrero R., Peregrina J. M., Bernardes G. J. L., Martínez A., Fiammengo R., Corzana F. (2024). JACS Au.

[cit18] Rosenbaum P., Artaud C., Bay S., Ganneau C., Campone M., Delaloge S., Gourmelon C., Loirat D., Medioni J., Pein F., Sablin M. P., Tredan O., Varga A., Leclerc C. (2020). Cancer Immunol. Immunother..

[cit19] Mehta A. Y., Veeraiah R. K. H., Dutta S., Goth C. K., Hanes M. S., Gao C., Stavenhagen K., Kardish R., Matsumoto Y., Heimburg-Molinaro J., Boyce M., Pohl N. L. B., Cummings R. D. (2020). Cell Chem. Biol..

[cit20] Frush H. L., Isbell H. S. (1934). J. Res. Natl. Bur. Stand..

[cit21] Basu P., Crich D. (2025). J. Am. Chem. Soc..

[cit22] Liu W., He P., Shang S., Tan Z. (2023). J. Carbohydr. Chem..

[cit23] Yu F., McConnell M. S., Nguyen H. M. (2015). Org. Lett..

[cit24] Wei G., Lv X., Du Y. (2008). Carbohydr. Res..

[cit25] Sommer R., Hauck D., Titz A. (2017). ChemistrySelect.

[cit26] Wells L., Vosseller K., Hart G. W. (2001). Science.

[cit27] Both P., Green A. P., Gray C. J., Šardzík R., Voglmeir J., Fontana C., Austeri M., Rejzek M., Richardson D., Field R. A., Widmalm G., Flitsch S. L., Eyers C. E. (2014). Nat. Chem..

[cit28] Mendonça-Previato L., Penha L., Garcez T. C., Jones C., Previato J. O. (2013). Glycoconjugate J..

[cit29] De Leon C. A., Lang G., Saavedra M. I., Pratt M. R. (2018). Org. Lett..

[cit30] Lefever M. R., Szab L. Z., Anglin B., Ferracane M., Hogan J., Cooney L., Polt R. (2012). Carbohydr. Res..

[cit31] Galesic A., Rakshit A., Cutolo G., Pacheco R. P., Balana A. T., Moon S. P., Pratt M. R. (2021). ACS Chem. Biol..

[cit32] Warnes M. E., Fascione M. A. (2024). Chem. – Eur. J..

[cit33] Dorst K. M., Engström O., Angles d’Ortoli T., Mobarak H., Ebrahemi A., Fagerberg U., Whitfield D. M., Widmalm G. (2024). Carbohydr. Res..

[cit34] Lu S.-R., Lai Y.-H., Chen J.-H., Liu C.-Y., Mong K.-K. T. (2011). Angew. Chem., Int. Ed..

[cit35] Evans V., Mahon M. F., Webster R. L. (2014). Tetrahedron.

[cit36] Mayato C., Dorta R. L., Vázquez J. T. (2008). Tetrahedron Lett..

[cit37] Zhang Z., Ollmann I. R., Ye X. S., Wischnat R., Baasov T., Wong C. H. (1999). J. Am. Chem. Soc..

[cit38] Frem D., Urban D., Norsikian S., Beau J. (2017). Eur. J. Org. Chem..

[cit39] Ikemoto N., Kim O. K., Lo L.-C., Satyanarayana V., Chang M., Nakanishi K. (1992). Tetrahedron Lett..

[cit40] Pilgrim W., Murphy P. V. (2010). J. Org. Chem..

