# Supplementary material for: Efficient synthesis of O-glycosylated amino acids
Source: RSC Chem Biol. 2025 May 7;6(6):851–6. doi: 10.1039/d5cb00076a (PMC12070420; doi:10.1039/d5cb00076a)
Supplement: CB-006-D5CB00076A-s001 [file CB-006-D5CB00076A-s001.pdf]

Supplementary Information (SI) for RSC Chemical Biology.
This journal is © The Royal Society of Chemistry 2025

Supporting Information

Efficient Synthesis of *O*-Glycosylated amino acids

Felicity J. Frank, Rebecca A. Lawson and Tom E. McAllister*

Chemistry, School of Natural and Environmental Sciences, Newcastle University, Newcastle upon Tyne, NE1 7RU.

Supporting Information

Cost of commercial Fmoc-amino acids 4

Supplementary Figures 5

General experimental information 8

Fmoc-Thr-OMe 6 ... 9

Fmoc-Thr-OAllyl S1 ... 10

Fmoc-Ser-OAllyl S2 ... 10

Typical procedure for Glycosylation 11

Fmoc-Thr[GalNAc(Ac)$_3$-α-D]-OMe α7 ... 12

Fmoc-Thr[GalNAc(Ac)$_3$-β-D]-OMe β7 ... 13

Fmoc-Ser[GalNAc(Ac)$_3$-α-D]-OMe α8 ... 14

Fmoc-Ser[GalNAc(Ac)$_3$-β-D]-OMe β8 ... 15

Fmoc-Thr[GlcNAc(Ac)$_3$-α-D]-OMe α9 ... 16

Fmoc-Thr[GlcNAc(Ac)$_3$-β-D]-OMe β9 ... 17

Fmoc-Ser[GlcNAc(Ac)$_3$-β-D]-OMe β10 ... 18

Typical procedure for demethylation with LiI. 19

Fmoc-Thr[GalNAc(Ac)$_3$-α-D]-OH α1 ... 19

Fmoc-Thr[GalNAc(Ac)$_3$-β-D]-OH β1 ... 20

Fmoc-Ser[GalNAc(Ac)$_3$-α-D]-OH α2 ... 21

Fmoc-Ser[GalNAc(Ac)$_3$-β-D]-OH β2 ... 22

Fmoc-Thr[GlcNAc(Ac)$_3$-β-D]-OH β12 ... 23

Fmoc-Ser[GlcNAc(Ac)$_3$-β-D]-OH β13 ... 23

Anomerisation time course experiment 24

Calculation of Synthesis cost 26

NMR Spectra 30

Fmoc Thr-OMe 6 ... 30

Fmoc-Thr[GalNAc(Ac)3-α-D]-OMe α7 ... 32

Fmoc-Thr[GalNAc(Ac)3-β-D]-OMe β7 ... 34

Fmoc-Ser[GalNAc(Ac)3-α-D]-OMe α8 ... 36

Fmoc-Ser[GalNAc(Ac)3-β-D]-OMe β8 ... 38

Fmoc-Thr[GlcNAc(Ac)3-α-D]-OMe α9 ... 40

Fmoc-Thr[GlcNAc(Ac)3-β-D]-OMe β9 ... 42

Fmoc-Ser[GlcNAc(Ac)3-β-D]-OMe β10 ... 44

Fmoc-Thr[GalNAc(Ac)3-α-D]-OH α1 ... 46

Fmoc-Thr[GalNAc(Ac)3-β-D]-OH β1 48
Fmoc-Ser[GalNAc(Ac)3-α-D]-OH α2 50
Fmoc-Ser[GalNAc(Ac)3-β-D]-OH β2 52
Supplementary references 54

Cost of commercial Fmoc-amino acids

Name; CAS	Supplier	Amount/g	Price/£	£/g	rMM	mmol	£/mmol	Link
Fmoc-Thr[GalNAc(Ac)3-α-D]-OH; 116783-35-8	Sigma Aldrich	0.1	770	7700	670.237	0.149	5160.825	772437-100MG
	Doug Discovery	0.1	477	4770	670.237	0.149	3197.030	F567280
	**BLD Pharm**	**0.25**	**1049**	**4196**	**670.237**	**0.373**	**2812.314**	**BD131423**
Fmoc-Thr(PO(OBzl)OH)-OH; 175291-56-2	Key Organics	5	462	92.4	511.467	9.776	47.260	AS-75165
	**Doug Discovery**	**5**	**222**	**44.4**	**511.467**	**9.776**	**22.709**	**M03392**
	BLD Pharm	5	234	46.8	511.467	9.776	23.937	175291-56-2
Fmoc-Thr(tBu)-OH; 71989-35-0	**Doug Discovery**	**100**	**84**	**0.84**	**397.471**	**251.591**	**0.334**	**M03389**
	Biosynth	100	108.45	1.0845	397.471	251.591	0.431	FF15778
	Key Organics	100	90	0.9	397.471	251.591	0.358	AS-14178
Fmoc-Ser[GalNAc(Ac)3-α-D]-OH; 120173-57-1	Sigma Aldrich	0.1	755	7550	656.22	0.152	4954.461	772445-100MG
	**Key Organics**	**0.25**	**552**	**2208**	**656.22**	**0.381**	**1448.934**	**BS-49043**
	BLD Pharma	0.25	556	2224	656.22	0.381	1459.433	BD131411
Fmoc-Ser(PO(OBzl)OH)-OH; 158171-14-3	Biosynth	5	350	70	497.43	10.052	34.820	FF47773
	**Doug Discovery**	**5**	**141**	**28.2**	**497.43**	**10.052**	**14.028**	**M03387**
	BLD Pharm	5	149	29.8	497.43	10.052	14.823	158171-14-3
Fmoc-Ser(tBu)-OH; 71989-33-8	**Doug Discovery**	**100**	**53**	**0.53**	**383.444**	**260.794**	**0.203**	**M03382**
	BLD Pharm	100	61	0.61	383.444	260.794	0.234	BD8607
	Key Organics	100	120	1.2	383.444	260.794	0.460	DS-13762

Table S1: Commercial pricing of amino acids. Correct as of 27th March 2025. The lowest prices we could identify, as listed on the websites for each compound from 3 separate suppliers are shown; cheaper options may be available. The cheapest identified is shown in bold for each compound and was used for price comparison.

Supplementary Figures

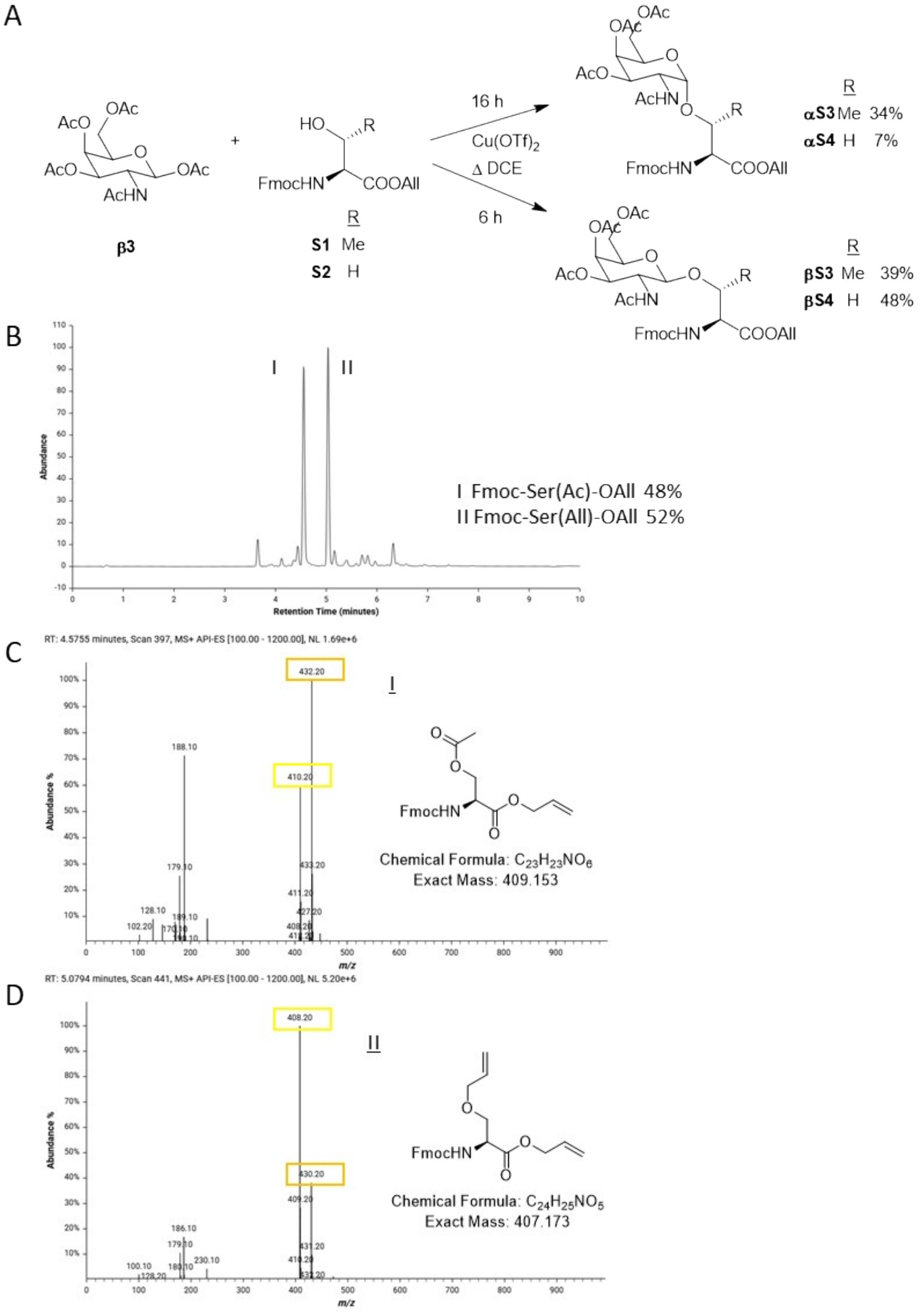

Figure S1: A) Initial experiments with **S1** and **S2** were carried out as per the general procedure described in the experimental section and using the conditions described in Table 1, main text. Yields are for isolated material, which was used in ^{1}H NMR experiments to assign α/β stereochemistry. B-D) Side products isolated from the reaction forming **αS4**. B) UV absorbance at 280 nm from LCMS with calculated relative areas for major peaks **I** & **II**, which we putatively assign as **I**: Fmoc-Ser(Ac)-OAll and

II: Fmoc-Ser(All)-OAll based on the corresponding mass spectrometry results: C) Mass spectrometry results for peak I; 410.20 *m/z* = $[M+H]^+$ highlighted in yellow and 432.20 *m/z* = $[M+Na]^+$ highlighted in orange; D) peak II; 408.20 *m/z* = $[M+H]^+$ (yellow) and 430.20 *m/z* = $[M+Na]^+$ (orange).

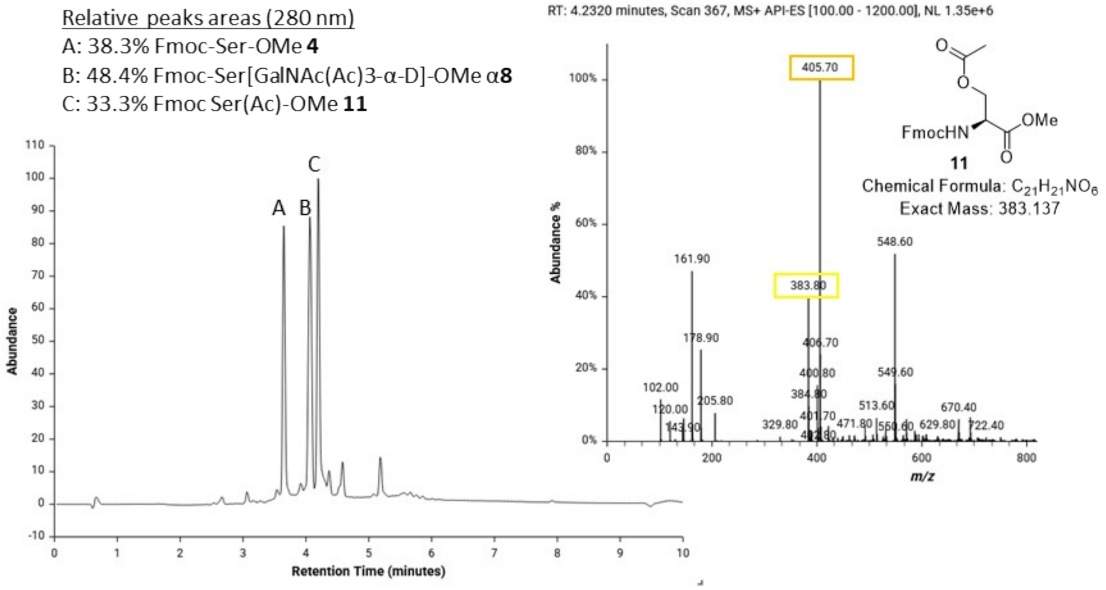

Figure S2. LCMS analysis of the reaction between 1 equiv. GalNAc donor **β3**, 1 equiv. $Cu(OTf)_2$ and 1 equiv. Fmoc-Ser-OMe **4** (table 1, entry 7) after 10 h (compound numbering as in main text). UV absorbance at 280 nm with calculated relative areas for the peaks labelled A-C. Inset: Mass spectrometry results for peak C; $[M+H]^+$ highlighted in yellow and $[M+Na]^+$ highlighted in orange.

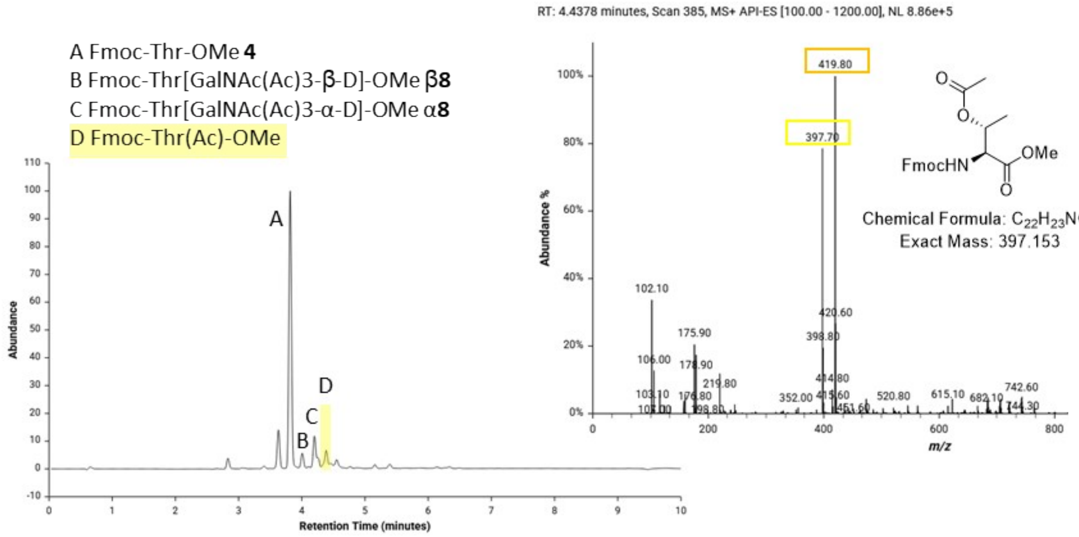

Figure S3. LCMS analysis of the reaction between 1 equiv. GalNAc donor β**3**, 1 equiv. $Cu(OTf)_2$ and 5 equiv. Fmoc-Thr-OMe **6** (table 1, entry 15) after 16 h (compound numbering as in main text). UV absorbance at 280 nm with main peaks labelled. Inset: Mass spectrometry results for peak D corresponding to Fmoc-Thr(Ac)-OMe; $[M+H]^+$ highlighted in yellow and $[M+Na]^+$ highlighted in orange.

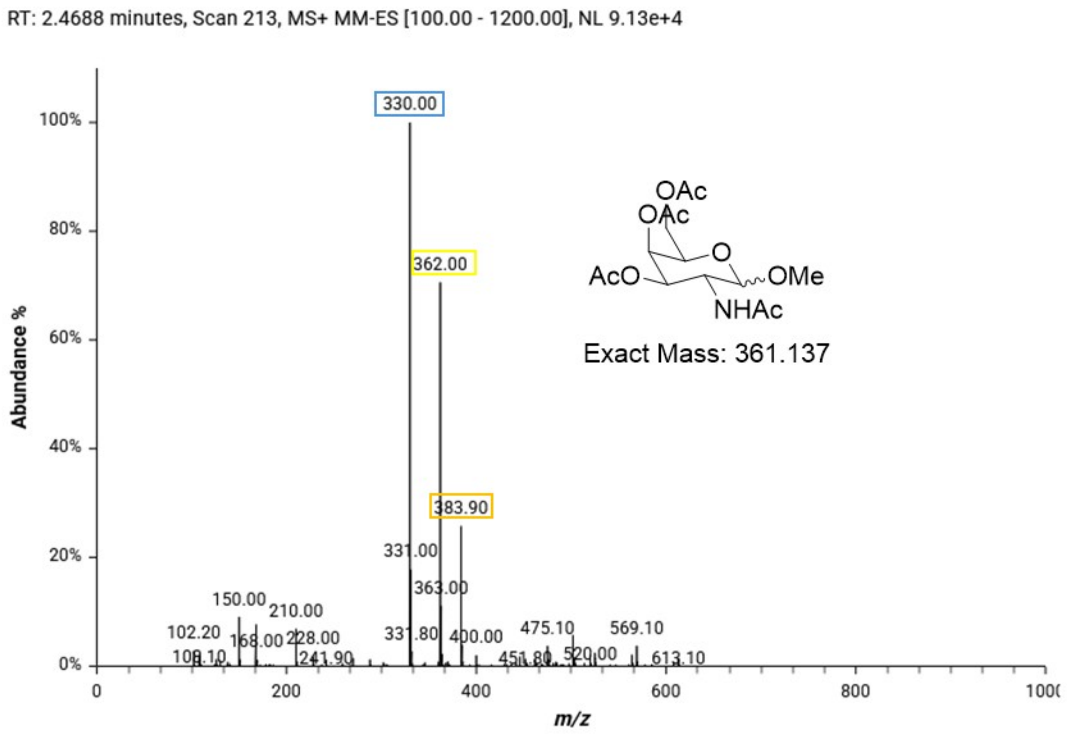

Figure S4. Mass spectrum showing formation of methyl 2-amino-2-deoxy-α/β-D-galactopyranoside by quenching an aliquot from the anomerisation reaction with methanol. 362.00 *m/z* [M+H]$^+$ highlighted in yellow, 383.90 *m/z* [M+Na]$^+$ highlighted in orange and the oxocarbenium ion/dioxalenium ion arising from neutral loss of methanol 330 *m/z* highlighted in blue.

General experimental information

^{1}H and ^{13}C NMR spectra were recorded directly with Bruker Advance III HD 700 MHz, a Jeol Lambda 500 MHz, Jeol ECS-400 MHz or Brucker Avance 300 MHz. LCMS data was obtained from samples either diluted with MeCN or MeOH using an Agilent Infinity 1290 II UPLC using a Raptor C_{18} LC column (2.7 µm particle size, 100 × 3.0 mm) coupled with Agilent MSD-XT. Each LCMS run used a solvent composition of MeCN:water with 0.1% (*v/v*) formic acid, from 5 to 95% MeCN over 10 mins. HRMS data was obtained from samples diluted in MeCN using a Waters G2-XS_QToF. Specific rotation ([α]) was calculated via measurement of observed rotation of each compound in DMSO using an Optical Activity PolAAr 2001 polarimeter. IR spectra were obtained as neat samples using a Varian 800 FT-IR Scimitar Series spectrometer scanning from 4000-600 cm^{-1}. Chemicals were purchased from Sigma Aldrich, Doug discovery (Fluorochem) or ThermoFisher Scientific and used without further purification.

Fmoc-Thr-OMe 6

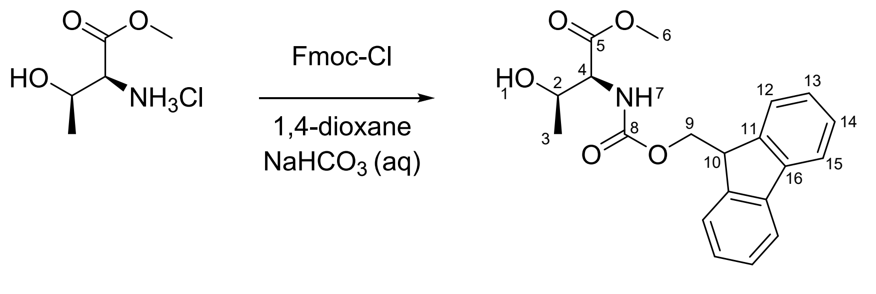

To a 100 mL rbf was added Thr-OMe HCl (2 g, 11.79 mmol) and aqueous $NaHCO_3$ (2.4 M, 20 mL, 47.6 mmol, 4 equiv.) and stirred for 1 min. 1,4-dioxane (6.06 mL) and Fmoc Cl (3.05 g, 11.79 mmol, 1 equiv.) was added and the reaction mixture was stirred vigorously for 1 hour. The reaction mixture was poured over water (10 mL), extracted with EtOAc (2 × 10 mL) and the combined organic extracts washed with brine (1 × 20 mL) and water (1 × 20 mL). The organic later was dried over $MgSO_4$, filtered and solvent removed under reduced pressure to give Fmoc-Thr-OMe as a white solid (4.17g, 99%). The crude Fmoc Thr-OMe taken forward without further purification due to high purity shown in ^{1}H NMR.

Data matched literature reports.[1]

^{1}H NMR (400 MHz, $CDCl_3$) δ 7.77 (d, *J* = 7.5 Hz, 2H, H^{15}), 7.62 (t, *J* = 6.2 Hz, 2H, H^{12}), 7.41 (t, *J* = 7.5 Hz, 2H, H^{14}), 7.32 (t, *J* = 7.5 Hz, 2H, H^{13}), 5.57 (s, 1H, H^7), 4.43 (d, *J* = 7.1 Hz, 2H, H^9), 4.36 (t, *J* = 7.0 Hz, 2H, $H^{2,4}$), 4.25 (t, *J* = 7.0 Hz, 1H, H^{10}), 3.79 (s, 3H, H^6), 1.89 (s, 1H, H^1), 1.26 (d, *J* = 6.2 Hz, 3H, H^3). **^{13}C NMR** (101 MHz, $CDCl_3$) δ 171.8 (C^5), 156.9 (C^8), 144.0/143.8 ($C^{16/16'}$), 141.5 (C^{11}), 127.9 (C^{14}), 127.2 (C^{13}), 125.2 (C^{12}), 120.2 (C^{15}), 68.1 (C^2), 67.4 (C^9), 59.1 (C^4), 52.8 (C^6), 47.3 (C^{10}), 20.0 (C^3).

Fmoc-Thr-OAllyl S1

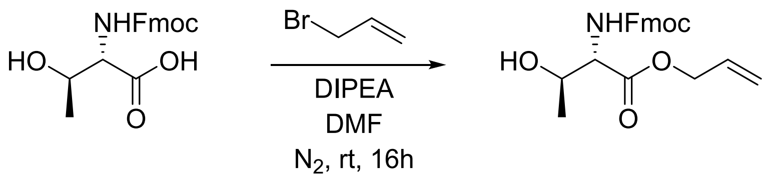

To a 50 mL rbf, under nitrogen, was added Fmoc Thr OH (1g, 2.93 mmol), dry DMF (12 mL), allyl bromide (506 uL, 5.86 mmol), DIPEA (1.02 mL, 5.86 mmol) and stirred at r.t. for 16 hours. The reaction mixture was diluted with ethyl acetate (15 mL) and washed with brine (4 x 30 mL). The organic layer was dried over $MgSO_4$, filtered and removed solvent under reduced pressure to give a white solid. The crude reaction mixture was purified by silica gel column chromatography (3:1 Hexane:Ethyl Acetate) to give Fmoc Thr OAllyl (1.10 g, 99%) as a white solid.

Data matched literature reports.[2]

Fmoc-Ser-OAllyl S2

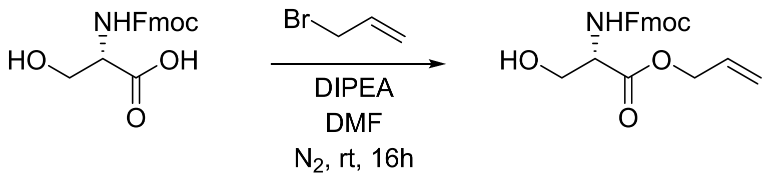

Fmoc Ser-OAllyl was prepared using the same procedure for the synthesis of Fmoc Thr OAllyl. This resulted in the isolation of Fmoc Ser OAllyl (1.08 g, 93%) as a white solid.

Data matched literature reports.[3]

Typical procedure for Glycosylation

To a 2-neck 50 mL rbf, under a flow nitrogen gas, was added donor (*N*-acetyl-β-D-galactosamine tetraacetate β or *N*-acetyl-β-D-glucosamine tetraacetate, 100 mg, 0.258 mmol), $Cu(OTf)_2$ (93 mg, 0.258 mmol, 1 equiv. relative to donor), DCE (5 mL) and acceptor (Fmoc-Thr-OMe, 458 mg or Fmoc-Ser-OMe 440 mg, 1.29 mmol, 5 equiv. relative to donor). The reaction mixture was degassed three times and stirred at reflux (1.6 to 16 hours depending on reaction). The reaction mixture was cooled, diluted with dichloromethane (10 mL) and washed with water (3 × 15 mL). The organic layer was dried over $MgSO_4$, filtered and the solvent removed under reduced pressure to give a brown oil. The crude reaction mixture was purified by silica gel column chromatography (3:1 EtOAc:Hexane) to afford glycosylated product.

Variations on this procedure were conducted as outlined in Table 1.

Fmoc-Thr[GalNAc(Ac)$_3$-α-D]-OMe α7

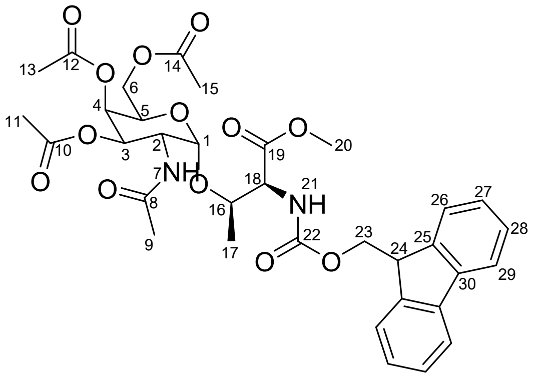

Followed typical glycosylation procedure, in which the donor was *N*-acetylgalactosamine tetra acetate and acceptor was Fmoc-Thr-OMe (458 mg, 1.29 mmol, 5 equiv.) and reacted for 16 hours. Fmoc-Thr[GalNAc(Ac)$_3$-β-D]-OMe (β, 35 mg, 20%) and Fmoc-Thr[GalNAc(Ac)$_3$-α-D]-OMe (α, 69 mg, 39%) were each isolated as light brown oils following purification by silica gel column chromatography eluting with 3:1 EtOAc:Hexane. Characterisation data below corresponds to α**7**.

R$_f$: 0.19 (2:1 EtOAc:Hexane). **IR** (neat): v_{max}/cm^{-1} 3329.2 (N-H, w, broad), 3072.3 – 2965.7 (C-H, w), 1753.1 (C=O, s), 1662.1 (C=C, m). **HRMS:** (ESI)+ calcd for $C_{34}H_{40}N_2O_{13}$ [M+H]$^+$: 685.2603, found 685.2598. **^{1}H NMR** (400 MHz, $CDCl_3$) δ 7.78 (d, *J* = 7.5 Hz, 2H, H^{29}), 7.64 (dd, *J* = 7.5, 2.9 Hz, 2H, H^{26}), 7.41 (td, *J* = 7.5, 2.7 Hz, 2H, H^{28}), 7.34 (ddd, *J* = 10.2, 5.1, 2.5 Hz, 2H, H^{27}), 5.82 (d, *J* = 9.7 Hz, 1H, H^7), 5.63 (d, *J* = 9.6 Hz, 1H, H^{21}), 5.38 (d, *J* = 3.2 Hz, 1H, H^4), 5.09 (dd, *J* = 11.4, 3.2 Hz, 1H, H^3), 4.87 (d, *J* = 3.7 Hz, 1H, H^1), 4.55 (td, *J* = 10.5, 3.6 Hz, 1H, H^2), 4.49 – 4.39 (m, 3H, H^{18,23}), 4.31 – 4.24 (m, 2H, H^{16,24}), 4.21 (t, *J* = 6.4 Hz, 1H, H^5), 4.14 – 4.03 (m, 2H, H^6), 3.74 (s, 3H, H^{20}), 2.16 (s, 3H, H$^{11/13/15}$), 2.03 (s, 3H, H$^{11/13/15}$), 2.00 (s, 3H, H$^{11/13/15}$), 1.99 (s, 3H, H^9), 1.32 (d, *J* = 6.4 Hz, 3H, H^{17}). **^{13}C NMR** (101 MHz, $CDCl_3$) δ 171.5 (C^{19}), 171.1 (C^{10}), 170.5 (C$^{8/12}$), 170.5 (C$^{8/12}$), 170.4 (C^{14}), 156.7 (C^{22}), 143.9/143.8 (C$^{30/30'}$), 141.5 (C^{25}), 127.9 (C^{28}), 127.3 (C^{27}), 125.2/125.2 (C$^{26/26'}$), 120.2/120.2 (C$^{29/29'}$), 100.1 (C^1), 68.5 (C^3), 67.5 (C^4), 67.5 (C^5), 67.4 (C^{23}), 62.2 (C^6), 58.6 (C^{18}), 52.8 (C^{20}), 47.7 (C^2), 47.3 (C^{24}), 23.3 (C^9), 20.9 (C$^{11/13/15}$), 20.9 (C$^{11/13/15}$), 20.8 (C$^{11/13/15}$), 18.3 (C^{17}).

Fmoc-Thr[GalNAc(Ac)$_3$-β-D]-OMe β7

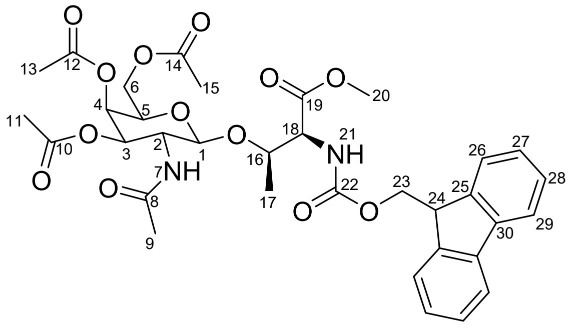

Followed typical glycosylation procedure, in which the donor was *N*-acetylgalactosamine tetra acetate and acceptor was Fmoc Thr-OMe (458 mg, 1.29 mmol, 5 equiv.) and reacted for 1 hour 40 mins. Fmoc-Thr[GalNAc(Ac)$_3$-β-D]-OMe was isolated (142 mg, 82%) as a white solid following purification by silica gel column chromatography eluting with 3:1 EtOAc:Hexane.

R_f: 0.07 (2:1 EtOAc:Hexane). **IR** (neat): v_{max}/cm^{-1} 3331.3 (N-H, w, broad), 3092.3 – 2954.4 (C-H, w), 1745.0 (C=O, s), 1665.7 (C=C, m). **HRMS:** (ESI)+ calcd for $C_{34}H_{40}N_2O_{13}$ [M+H]$^+$: 685.2603, found 685.2599. **^{1}H NMR** (300 MHz, $CDCl_3$) δ 7.76 (d, *J* = 7.4 Hz, 2H, H^{29}), 7.66 (t, *J* = 6.5 Hz, 2H, H^{26}), 7.40 (t, *J* = 6.8 Hz, 2H, H^{28}), 7.32 (td, *J* = 7.4, 1.3 Hz, 2H, H^{27}), 5.76 (d, *J* = 9.0 Hz, 1H, H^{21}), 5.57 (d, *J* = 8.4 Hz, 1H, H^{7}), 5.34 (d, *J* = 3.0 Hz, 1H, H^{4}), 5.28 (dd, *J* = 10.3, 4.2 Hz, 1H, H^{3}), 4.70 (d, *J* = 8.3 Hz, 1H, H^{1}), 4.43 (td, *J* = 10.8, 7.1 Hz, 3H, H^{16,23}), 4.37 – 4.31 (m, 1H, H^{18}), 4.26 (t, *J* = 7.2 Hz, 1H, H^{24}), 4.09 (d, *J* = 6.7 Hz, 2H, H^{6}), 3.93 – 3.79 (m, 2H, H^{2,5}), 3.75 (s, 3H, H^{20}), 2.13 (s, 3H, H^{13}), 2.05 (s, 3H, H^{11}), 2.00 (s, 3H, H^{15}), 1.95 (s, 3H, H^{9}), 1.20 (d, *J* = 6.3 Hz, 3H, H^{17}). **^{13}C NMR** (75 MHz, $CDCl_3$) δ 170.8 (C^{19}), 170.6 (C^{14}), 170.6 (C^{9,10}), 170.4 (C^{12}), 156.9 (C^{22}), 144.1/143.9 (C$^{30/30'}$), 141.4 (C^{25}), 127.8 (C^{28}), 127.2/127.2 (C^{27}), 125.4/125.3 (C^{26}), 120.1 (C^{29}), 99.5 (C^{1}), 75.0 (C^{16}), 70.6 (C^{5}), 69.6 (C^{3}), 67.3 (C^{23}), 66.7 (C^{4}), 61.3 (C^{6}), 58.7 (C^{18}), 52.7 (C^{20}), 52.0 (C^{2}), 47.3 (C^{24}), 23.6 (C^{9}), 20.8 (C$^{11/13/15}$), 20.8 (C$^{11/13/15}$), 17.4 (C^{17}).

Fmoc-Ser[GalNAc(Ac)$_3$-α-D]-OMe α8

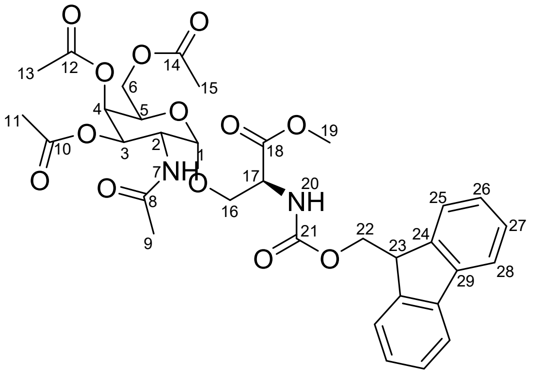

Followed typical glycosylation procedure, in which the donor was *N*-acetylgalactosamine tetra acetate and acceptor was Fmoc Ser-OMe (88 mg, 0.258 mmol, 1 equiv.) and reacted for 10 hours. Fmoc-Ser[GalNAc(Ac)$_3$-α-D]-OMe was isolated (20 mg, 12%) as a light brown oil following purification by silica gel column chromatography eluting with 3:1 EtOAc:Hexane.

R$_f$: 0.22 (2:1 EtOAc:Hexane). **IR** (neat): v_{max}/cm^{-1} 3329.8 (N-H, w, broad), 3062.1 – 2899.2 (C-H, w), 1755.2 (C=O, s), 1666.6 (C=C, m). **HRMS:** (ESI)+ calcd for $C_{33}H_{38}N_2O_{13}$ [M+H]$^+$: 671.2447, found 671.2443. **^{1}H NMR** (400 MHz, $CDCl_3$) δ 7.77 (d, *J* = 7.5 Hz, 2H, H^{28}), 7.62 (d, *J* = 7.6 Hz, 2H, H^{25}), 7.41 (t, *J* = 7.4 Hz, 2H, H^{26}), 7.33 (t, *J* = 7.4 Hz, 2H, H^{27}), 5.82 (d, *J* = 8.2 Hz, 1H, H^{20}), 5.67 (d, *J* = 9.7 Hz, 1H, H^{7}), 5.37 (d, *J* = 3.2 Hz, 1H, H^{4}), 5.10 (dd, *J* = 11.8, 3.1 Hz, 1H, H^{3}), 4.84 (d, *J* = 3.6 Hz, 1H, H^{1}), 4.62 – 4.51 (m, 2H, H^{2,17}), 4.44 (d, *J* = 7.1 Hz, 2H, H^{22}), 4.25 (t, *J* = 6.9 Hz, 1H, H^{23}), 4.15 – 4.01 (m, 3H, H^{5,6}), 4.00 – 3.89 (m, 2H, H^{16}), 3.79 (s, 3H, H^{19}), 2.16 (s, 3H, H^{13}), 2.00 (s, 6H, H^{11,15}), 1.97 (s, 3H, H^{9}).**^{13}C NMR** (176 MHz, $CDCl_3$) δ 171.1 (C^{10}), 170.6 (C^{14,18}), 170.4 (C^{12}), 170.3 (C^{8}), 156.0 (C^{21}), 143.8 (C^{29}), 141.5 (C^{24}), 128.0/128.0 (C$^{27/27'}$), 127.3/127.3 (C$^{26/26'}$), 125.2 (C^{25}), 120.2 (C^{28}), 99.3 (C^{1}), 69.8 (C^{22}), 68.4 (C^{3}), 67.5 (C^{22}), 67.3 (C^{4}), 62.0 (C^{6}), 54.5 (C^{17}), 53.0 (C^{19}), 47.8 (C^{2}), 47.2 (C^{23}), 23.4 (C^{9}), 20.9 (C$^{11/13/15}$), 20.9 (C$^{11/13/15}$), 20.8 (C$^{11/13/15}$).

Fmoc-Ser[GalNAc(Ac)$_3$-β-D]-OMe β8

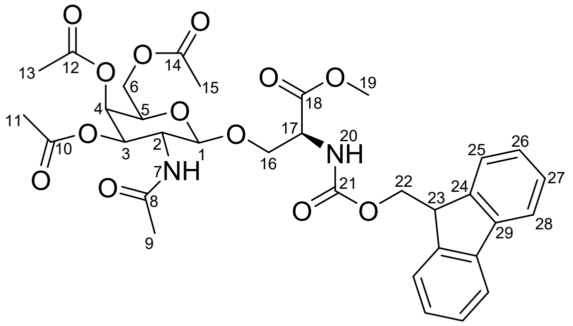

Followed typical glycosylation procedure, in which the donor was *N*-acetylgalactosamine tetra acetate and acceptor was Fmoc Ser-OMe (440 mg, 1.29 mmol, 5 equiv.) and reacted for 1 hour 40 mins. Fmoc-Ser[GalNAc(Ac)$_3$-β-D]-OMe was isolated (114 mg, 66%) as a white solid following purification by silica gel column chromatography eluting with 3:1 EtOAc:Hexane.

R$_f$: 0.10 (2:1 EtOAc:Hexane). **IR** (neat): v_{max}/cm^{-1} 3317.8 (N-H, m, broad), 3018.0 – 2955.0 (C-H, w), 1741.7 (C=O, s), 1662.2 (C=C, s). **HRMS:** (ESI)+ calcd for $C_{33}H_{38}N_2O_{13}$ $[M+H]^+$: 671.2447, found 671.2443. **^{1}H NMR** (300 MHz, $CDCl_3$) δ 7.77 (d, *J* = 8.5 Hz, 2H, H^{28}), 7.64 (d, *J* = 7.4 Hz, 2H, H^{25}), 7.40 (td, *J* = 7.5, 1.3 Hz, 2H, H^{27}), 7.32 (tt, *J* = 7.4, 1.5 Hz, 2H, H^{26}), 5.79 (d, *J* = 8.2 Hz, 1H, H^{20}), 5.55 (d, *J* = 8.6 Hz, 1H, H^{7}), 5.33 (dd, *J* = 3.4, 1.2 Hz, 1H, H^{4}), 5.18 (dd, *J* = 11.3, 3.4 Hz, 1H, H^{3}), 4.61 (d, *J* = 8.4 Hz, 1H, H^{1}), 4.55 – 4.38 (m, 3H, $H^{17,22}$), 4.26 – 4.17 (m, 2H, $H^{16,23}$), 4.11 (d, *J* = 6.5 Hz, 2H, H^{6}), 3.96 (dt, *J* = 11.3, 8.5 Hz, 1H, H^{2}), 3.89 – 3.82 (m, 2H, $H^{5,23}$), 3.76 (s, 3H, H^{19}), 2.13 (s, 3H, H^{13}), 2.03 (s, 3H, H^{15}), 2.00 (s, 3H, H^{11}), 1.85 (s, 3H, H^{9}). **^{13}C NMR** (75 MHz, $CDCl_3$) δ 170.9 (C^{8}), 170.7 (C^{10}), 170.6 (C^{14}), 170.3 ($C^{12,18}$), 156.2 (C^{21}), 143.9/143.8 ($C^{29/29'}$), 141.4/141.4 ($C^{24/24'}$), 127.9 (C^{27}), 127.3/127.3 (C^{26}), 125.2 (C^{25}), 120.2/120.1 ($C^{28/28'}$), 101.5 (C^{1}), 70.9 (C^{5}), 70.0 (C^{3}), 69.0 (C^{16}), 66.9 (C^{22}), 66.7 (C^{4}), 61.5 (C^{6}), 54.3 (C^{17}), 52.9 (C^{19}), 51.2 (C^{2}), 47.3 (C^{23}), 23.4 (C^{9}), 20.8 ($C^{11/13/15}$), 20.8 ($C^{11/13/15}$).

Fmoc-Thr[GlcNAc(Ac)$_3$-α-D]-OMe α9

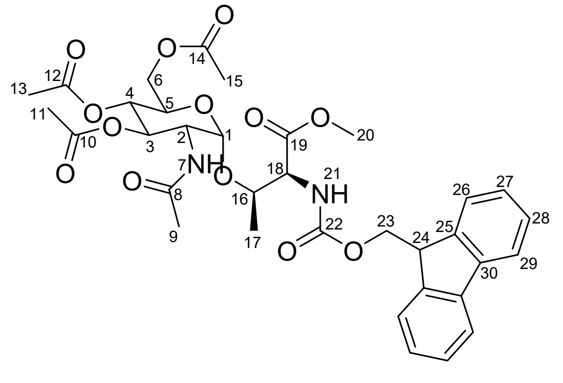

Followed typical glycosylation procedure, in which the donor was *N*-acetylglucosamine tetra acetate and acceptor was Fmoc Thr-OMe (458 mg, 1.29 mmol, 5 equiv.) and reacted for 16 hours. Fmoc-Thr[GlcNAc(Ac)$_3$-β-D]-OMe (β, 53 mg, 29%) and Fmoc-Thr[GlcNAc(Ac)$_3$-α-D]-OMe (α, 18 mg, 10%) were each isolated as light brown oils following purification by silica gel column chromatography eluting with 3:1 EtOAc:Hexane.

Characterisation data below corresponds to α**10**.

R$_f$: 0.23 (2:1 EtOAc:Hexane). **IR** (neat): v_{max}/cm^{-1} 3321.9 (N-H, w, broad), 3066.5 – 2955.3 (C-H, w), 1743.7 (C=O, s), 1663.6 (C=C, m). **HRMS:** (ESI)+ calcd for $C_{34}H_{40}N_2O_{13}$ [M+H]$^+$: 685.2603, found 685.2599. **^{1}H NMR** (400 MHz, $CDCl_3$) δ 7.78 (d, *J* = 7.7 Hz, 2H, H^{29}), 7.66 (t, *J* = 6.1 Hz, 2H, H^{26}), 7.47 – 7.38 (m, 2H, H^{28}), 7.38 – 7.30 (m, 2H, H^{27}), 5.88 (d, *J* = 9.6 Hz, 1H, H^{7}), 5.61 (d, *J* = 9.5 Hz, 1H, H^{21}), 5.17 (t, *J* = 10.0 Hz, 1H, H^{3}), 5.09 (t, *J* = 9.7 Hz, 1H, H^{4}), 4.84 (d, *J* = 3.8 Hz, 1H, H^{1}), 4.50 – 4.41 (m, 3H, H^{18,23}), 4.37 – 4.25 (m, 3H, H^{2,16,24}), 4.21 (dd, J = 12.3, 5.1 Hz, 1H, H^{6}), 4.15 – 4.06 (m, 1H, H^{6})K, 4.05 – 3.95 (m, 1H, H^{5}), 3.75 (s, 3H, H^{20}), 2.08 (s, 3H, H$^{11/13/15}$), 2.05 (s, 3H, H$^{11/13/15}$), 2.03 (s, 3H, H$^{11/13/15}$), 2.00 (s, 3H, H^{9}), 1.32 (d, *J* = 6.4 Hz, 3H, H^{17}). **^{13}C NMR** (101 MHz, $CDCl_3$) δ 171.5 (C^{19}), 171.4 (C$^{8/10/14}$), 170.7 (C$^{8/10/14}$), 170.5 (C$^{8/10/14}$), 169.4 (C^{12}), 156.7 (C^{22}), 143.8 (C^{30}), 141.5 (C^{25}), 128.0 (C^{28}), 127.3 (C^{27}), 125.3 (C^{26}), 120.2 (C^{29}), 99.6 (C^{1}), 71.3 (C^{3}), 68.5 (C^{5}), 68.4 (C^{4}), 67.6 (C^{23}), 62.2 (C^{6}), 58.6 (C^{18}), 52.8 (C^{20}), 51.8 (C^{2}), 47.3 (C^{24}), 23.2 (C^{9}), 20.9 (C$^{11/13/15}$), 20.8 (C$^{11/13/15}$), 20.8 (C$^{11/13/15}$), 18.4 (C^{17}).

Fmoc-Thr[GlcNAc(Ac)$_3$-β-D]-OMe β9

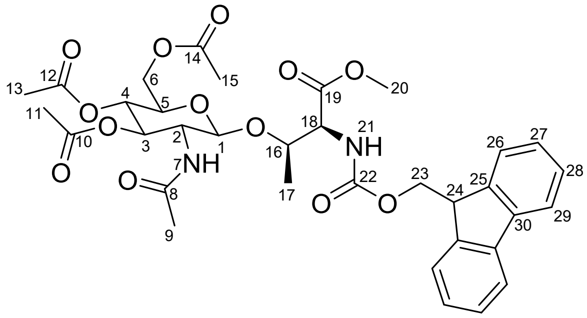

Followed typical glycosylation procedure, in which the donor was *N*-acetylglucosamine tetra acetate and acceptor was Fmoc Thr-OMe (458 mg, 1.29 mmol, 5 equiv.) and reacted for 1 hour 40 mins. Fmoc-Thr[GlcNAc(Ac)$_3$-β-D]-OMe was isolated (100 mg, 57%) as a white solid following purification by silica gel column chromatography eluting with 3:1 EtOAc:Hexane.

R_f: 0.10 (2:1 EtOAc:Hexane). **IR** (neat): v_{max}/cm^{-1} 3326.0 (N-H, w, broad), 2971.1 (C-H, w), 1743.7 – 1727.6 (C=O, s), 1659.8 (C=C, m). **HRMS:** (ESI)+ calcd for $C_{34}H_{40}N_2O_{13}$ $[M+H]^+$: 685.2603, found 685.2599. **^{1}H NMR** (300 MHz, $CDCl_3$) δ 7.76 (d, *J* = 7.4 Hz, 2H, H^{29}), 7.69 – 7.56 (m, 2H, H^{26}), 7.39 (t, *J* = 7.4 Hz, 2H, H^{28}), 7.31 (tt, *J* = 7.7, 1.9 Hz, 2H, H^{27}), 5.78 (d, *J* = 9.0 Hz, 1H, H^{21}), 5.74 (d, *J* = 8.4 Hz, 1H, H^7), 5.36 – 5.27 (m, 1H, H^3) 5.03 (t, *J* = 9.6 Hz, 1H, H^4), 4.72 (d, *J* = 8.3 Hz, 1H, H^1), 4.52 – 4.32 (m, 4H, $H^{16,18,23}$), 4.30 – 4.17 (m, 2H, $H^{6,24}$), 4.15 – 4.04 (m, 1H, H^6), 3.73 (s, 3H, H^{20}), 3.71 – 3.61 (m, 2H, $H^{2,5}$), 2.06 (s, 3H, H^{15}), 2.03 (s, 3H, H^{11}), 2.01 (s, 3H, H^{13}), 1.93 (s, 3H, H^9), 1.19 (d, *J* = 6.2 Hz, 3H, H^{17}). **^{13}C NMR** (75 MHz, $CDCl_3$) δ 171.0 (C^{10}), 170.8 ($C^{14/19}$), 170.8 ($C^{14/19}$), 170.5 (C^8), 169.5 (C^{12}), 156.9 (C^{22}), 144.0/143.9 ($C^{30/30'}$), 141.4/141.4 ($C^{25/25'}$), 127.9/127.8 ($C^{28/28'}$), 127.2/127.2 ($C^{27/27'}$), 125.3/125.2 ($C^{26/26'}$), 120.1/120.1 ($C^{29/29'}$), 98.9 (C^1), 74.8 (C^{16}), 72.0 (C^3), 71.7 (C^5), 68.7 (C^4), 67.3 (C^{23}), 62.1 (C^6), 58.7 (C^{18}), 55.2 (C^{20}), 52.6 (C^2), 47.3/47.2 ($C^{24/24'}$), 23.4 (C^9), 20.8 ($C^{11/13/15}$), 20.7 ($C^{11/13/15}$), 17.1 (C^{17}).

Fmoc-Ser[GlcNAc(Ac)$_3$-β-D]-OMe β10

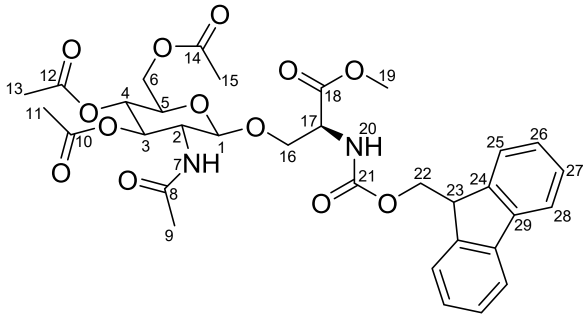

Followed typical glycosylation procedure, in which the donor was *N*-acetylglucosamine tetra acetate and acceptor was Fmoc Ser-OMe (440 mg, 1.29 mmol, 5 equiv.) and reacted for 1 hour 40 mins. Fmoc-Ser[GlcNAc(Ac)$_3$-β-D]-OMe was isolated (117 mg, 68%) as a white solid following purification by silica gel column chromatography eluting with 3:1 EtOAc:Hexane.

R$_f$: 0.18 (3:1 EtOAc:Hexane). **IR** (neat): v_{max}/cm^{-1} 3316.5 (N-H, m, broad), 2980.6 – 2890.5 (C-H, w), 1741.8 (C=O, s), 1665.1 (C=C, m). **HRMS:** (ESI)+ calcd for $C_{33}H_{38}N_2O_{13}$ $[M+H]^+$: 671.2447, found 671.2443. **^{1}H NMR** (300 MHz, $CDCl_3$) δ 7.81 – 7.70 (m, 2H, H^{28}), 7.63 (d, *J* = 7.5 Hz, 2H, H^{25}), 7.39 (t, *J* = 7.4 Hz, 2H, H^{27}), 7.31 (td, *J* = 7.4, 1.3 Hz, 2H, H^{26}), 5.84 (d, *J* = 8.5 Hz, 1H, H^{20}), 5.78 (d, *J* = 8.6 Hz, 1H, H^{7}), 5.23 (dd, *J* = 10.6, 9.3 Hz, 1H, H^{3}), 5.03 (t, *J* = 9.6 Hz, 1H, H^{4}), 4.63 (d, *J* = 8.2 Hz, 1H, H^{1}), 4.53 – 4.35 (m, 3H, $H^{17,22}$), 4.29 – 4.06 (m, 4H, $H^{6,16,23}$), 3.89 – 3.79 (m, 1H, $H^{2,16}$), 3.74 (s, 3H, H^{19}), 3.66 (ddd, *J* = 10.0, 4.8, 2.5 Hz, 1H, H^{5}), 2.06 (s, 3H, H^{15}), 2.02 (s, 3H, H^{11}), 2.01 (s, 3H, H^{13}), 1.84 (s, 3H, H^{9}). **^{13}C NMR** (75 MHz, $CDCl_3$) δ 171.0 (C^{10}), 170.8 ($C^{8/14}$), 170.8 ($C^{8/14}$), 170.3 (C^{18}), 169.5 (C^{12}), 156.2 (C^{21}), 143.8 (d, *J* = 9.6 Hz, C^{29}), 141.4 (d, *J* = 4.7 Hz, H^{24}), 127.9 (d, *J* = 3.1 Hz, H^{27}), 127.2 (d, *J* = 2.0 Hz, H^{26}), 125.2 (d, *J* = 2.1 Hz, H^{25}), 120.1 (d, *J* = 1.9 Hz, H^{28}), 100.9 (C^{1}), 72.3 (C^{3}), 72.0 (C^{5}), 70.0 (C^{16}), 68.6 (C^{4}), 67.0 (C^{22}), 62.1 (C^{6}), 54.4 (C^{17}), 54.3 (C^{2}), 52.8 (C^{19}), 47.3 (C^{23}), 23.2 (C^{9}), 20.8 ($C^{11/13/15}$), 20.8 ($C^{11/13/15}$), 20.7 ($C^{11/13/15}$).

Typical procedure for demethylation with LiI.

Fmoc-Thr[GalNAc(Ac)$_3$-α-D]-OH α1

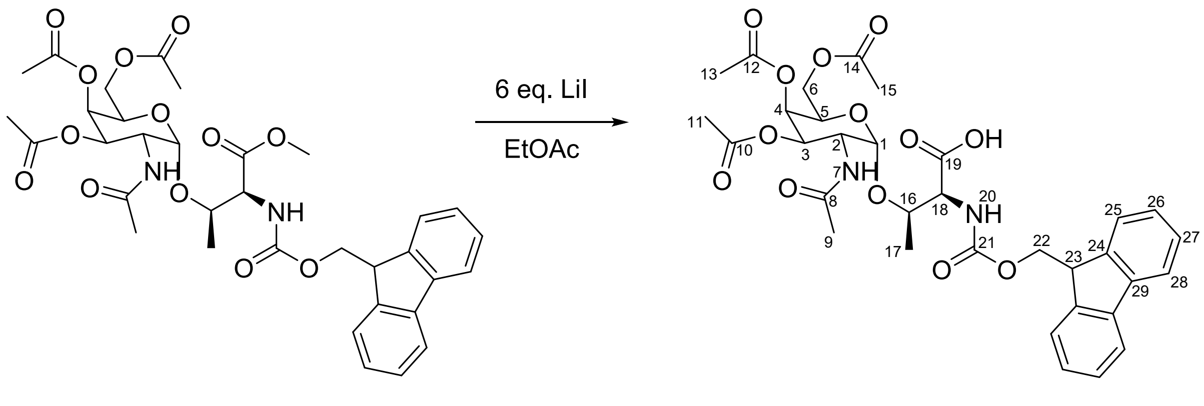

To a 2-neck 25 mL rbf, under nitrogen, was added Fmoc-Thr[GalNAc(Ac)$_3$-α-D]-OMe (281 mg, 0.410 mmol) and LiI (330 mg, 2.462 mmol). Dry EtOAc (4.1 mL, 10 mL/mmol) was added, degassed (x3) and stirred under reflux for 24 hours. The reaction mixture was diluted with EtOAc (5 mL), washed with 10% HCl (15mL) and sat. $Na_2O_3S_3$ (15 mL). The desired product was extracted from the organic layer with sat. $NaHCO_3$ (3 ×15 mL) and then the aqueous layer was acidified with 10% HCl. The desired product was extracted from the aqueous layer with EtOAc (3 × 15 mL), dried over $MgSO_4$, filtered and solvent removed under reduced pressure to give Fmoc-Thr[GalNAc(Ac)$_3$-α-D]-OH (120 mg, 44%) as a light brown oil.

Data matched literature reports. [4]

HRMS: (ESI)+ calcd for $C_{32}H_{36}N_2O_{13}$ [M+H]$^+$: 657.2290, found 657.2285. **[α]$_D$** = +176° (*c* = 0.1, DMSO) **^{1}H NMR** (700 MHz, MeOD) δ 7.83 (d, *J* = 7.6 Hz, 2H, H^{28}), 7.70 (ddd, *J* = 12.5, 7.5, 1.0 Hz, 2H, H^{25}), 7.41 (dddd, *J* = 9.3, 4.5, 2.6, 0.9 Hz, 2H, H^{27}), 7.33 (qd, *J* = 7.5, 1.2 Hz, 2H, H^{26}), 5.41 (dd, *J* = 3.3, 1.2 Hz, 1H, H^4), 5.08 (dd, *J* = 11.5, 3.3 Hz, 1H, H^3), 4.95 (d, *J* = 3.9 Hz, 1H*, H^1), 4.61 (dd, *J* = 10.8, 6.4 Hz, 1H, H^{22}), 4.48 (dd, *J* = 10.8, 6.2 Hz, 1H, H^{22}), 4.42 – 4.37 (m, 2H, H^{2,16}), 4.32 – 4.24 (m, 3H, H^{5,18,23}), 4.19 – 4.08 (m, 2H, H^6), 2.15 (s, 3H, H$^{11/13/15}$), 2.05 (s, 3H, H$^{11/13/15}$), 1.96 (s, 3H, H$^{11/13/15}$), 1.95 (s, 3H, H^9), 1.25 (d, *J* = 6.5 Hz, 3H, H^{17}). **signal partially overlaps with residual solvent peak.* **^{13}C NMR** (176 MHz, MeOD) δ 173.6 (C^8), 173.3 (C^{19}), 172.1 (C$^{10/12/14}$), 172.1 (C$^{10/12/14}$), 172.0 (C$^{10/12/14}$), 159.2 (C^{21}), 145.4/145.1 (C$^{29/29'}$), 142.7/142.7 (C$^{24/24'}$), 128.8 (C^{27}) 128.2 (C^{26}), 126.2/126.0 (C$^{25/25'}$), 121.0/121.0 (C$^{28/28'}$), 100.8 (C^1),

77.6 (C^{16}), 69.7 (C^{3}), 68.8 (C^{4}), 68.2 (C^{5}), 67.7 (C^{22}), 63.3 (C^{6}), 59.8 (C^{18}), 48.9 (C^{23}), 48.6 (C^{2}), 22.9 (C^{9}), 20.6 ($C^{11/13/15}$), 20.6 ($C^{11/13/15}$), 20.5 ($C^{11/13/15}$), 19.2 (C^{17}).

Fmoc-Thr[GalNAc(Ac)$_3$-β-D]-OH β1

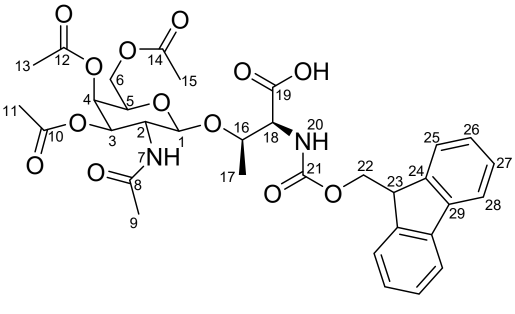

Followed demethylation procedure starting from Fmoc-Thr[GalNAc(Ac)$_3$-β-D]-OMe (200 mg, 0.292 mmol) and refluxed for 12 hours. Fmoc-Thr[GalNAc(Ac)$_3$-β-D]-OH was isolated (165 mg, 84%) as a colourless oil.

Data matched literature reports. [4]

HRMS: (ESI)+ calcd for $C_{33}H_{38}N_2O_{13}$ $[M+H]^+$: 671.2447, found 671.2441. **$[\alpha]_D$** = -22° (*c* = 0.1, DMSO) **^{1}H NMR** (700 MHz, MeOD) δ 7.82 (d, *J* = 7.6 Hz, 2H, H^{28}), 7.72 (dd, *J* = 12.5, 7.5 Hz, 2H, H^{25}), 7.41 (tdd, *J* = 7.5, 2.2, 1.3 Hz, 2H, H^{27}), 7.34 (tdd, *J* = 7.5, 2.0, 1.2 Hz, 2H, H^{26}), 5.35 (dd, *J* = 3.4, 1.2 Hz, 1H, H^{4}), 5.09 (dd, *J* = 11.3, 3.4 Hz, 1H, H^{3}), 4.61 (d, *J* = 8.5 Hz, 1H, H^{1}), 4.45 (qd, *J* = 6.4, 2.7 Hz, 1H, H^{16}), 4.43 – 4.35 (m, 2H, H^{22}), 4.28 (t, *J* = 7.2 Hz, 1H, H^{23}), 4.25 (d, *J* = 2.7 Hz, 1H, H^{18}), 4.18 (dd, *J* = 11.1, 7.6 Hz, 1H. H^{6}), 4.12 (dd, *J* = 11.7, 5.6 Hz, 1H, H^{6}), 4.06 (dd, *J* = 11.3, 8.4 Hz, 1H, H^{2}), 4.01 (ddd, *J* = 7.5, 6.1, 1.2 Hz, 1H, H^{5}), 2.12 (s, 3H, $H^{13/15}$), 2.04 (s, 3H, $H^{13/15}$), 1.97 (s, 3H, H^{11}), 1.96 (s, 3H, H^{9}), 1.22 (d, *J* = 6.4 Hz, 3H, H^{17}). **^{13}C NMR** (176 MHz, MeOD) δ 174.0 (C^{8}), 173.4 (C^{19}), 172.2 ($C^{12/14}$), 172.1 ($C^{12/14}$), 171.7 (C^{10}), 159.1 (C^{21}), 145.3/145.1 ($C^{29/29'}$) 142.6/142.6 ($C^{24/24'}$), 128.8 (C^{27}) 128.2/128.2 ($C^{26/26'}$), 126.4/126.3 ($C^{25/25'}$), 120.9/120.9 ($C^{28/28'}$), 101.7 (C^{1}), 76.8 (C^{16}), 71.9 (C^{3}), 71.6 (C^{5}), 68.3 (C^{22}), 67.8 (C^{4}), 62.2 (C^{6}), 60.0 (C^{18}), 51.6 (C^{2}), 48.4 (C^{23}), 22.9 (C^{9}), 20.6 ($C^{11/13/15}$), 20.5 ($C^{11/13/15}$), 18.0 (C^{17}).

Fmoc-Ser[GalNAc(Ac)$_3$-α-D]-OH α2

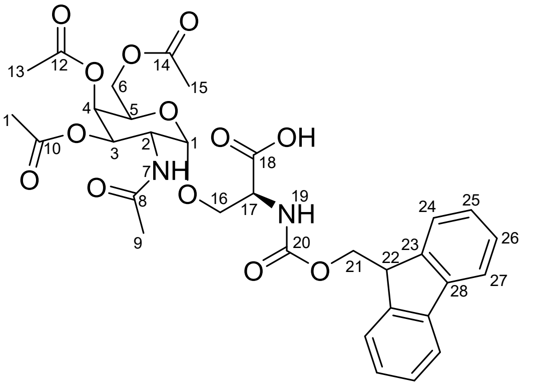

Followed demethylation procedure starting from Fmoc-Ser[GalNAc(Ac)$_3$-α-D]-OMe (73 mg, 0.109 mmol). Fmoc-Ser[GalNAc(Ac)$_3$-α-D]-OH was isolated (44 mg, 62%) as a light brown oil.

Data matched literature reports. [5]

HRMS: (ESI)+ calcd for $C_{32}H_{36}N_2O_{13}$ $[M+H]^+$: 657.2290, found 657.2285. **$[\alpha]_D$** = +80° (*c* = 0.1, DMSO) **^{1}H NMR** (700 MHz, MeOD) δ 7.81 (d, *J* = 7.5 Hz, 2H, H^{27}), 7.69 (dd, *J* = 11.2, 7.2 Hz, 2H, H^{24}), 7.40 (t, *J* = 7.5 Hz, 2H, H^{26}), 7.36 – 7.31 (m, 2H, H^{25}), 5.40 (dd, *J* = 3.3, 1.3 Hz, 1H, H^4), 5.15 (dd, *J* = 11.5, 3.3 Hz, 1H, H^3), 4.90* (d, *J* = 3.8 Hz, 1H, H^1), 4.52 – 4.38 (m, 4H, $H^{2,17,21}$), 4.25 (q, *J* = 6.5 Hz, 2H, $H^{5,22}$), 4.09 (dd, *J* = 16.3, 6.6 Hz, 1H, H^6), 4.03 (dd, *J* = 11.2, 7.1 Hz, 1H, H^6), 3.95 (t, *J* = 4.1 Hz, 2H, H^{16}), 2.14 (s, 3H, $H^{11/13/15}$), 1.96 (s, 3H, $H^{11/13/15}$), 1.95 (s, 3H, $H^{1/13/15}$), 1.93 (s, 3H, H^9). **signal partially overlaps with residual solvent peak.* **^{13}C NMR** (176 MHz, MeOD) δ 173.6 (C^8), 173.2 (C^{18}), 172.1 ($C^{10/12/14}$), 172.1 ($C^{10/12/14}$), 171.9 ($C^{10/12/14}$), 158.5 (C^{20}), 145.3/145.2 ($C^{28/28'}$), 142.7 (C^{23}), 128.8 (C^{26}), 128.2 (C^{25}), 126.2/126.1 ($C^{24/24'}$), 121.0 (C^{27}), 99.9 (C^1), 69.8 (C^{16}), 69.6 (C^3), 68.6 (C^4), 68.2 (C^5), 68.0 (C^{21}), 63.0 (C^6), 55.8 (C^{17}), 48.9 (C^2) 48.4 (C^{22}), 22.6 (C^9), 20.7 ($C^{11/13/15}$), 20.5 ($C^{11/13/15}$), 20.5 ($C^{11/13/15}$).

Fmoc-Ser[GalNAc(Ac)$_3$-β-D]-OH β2

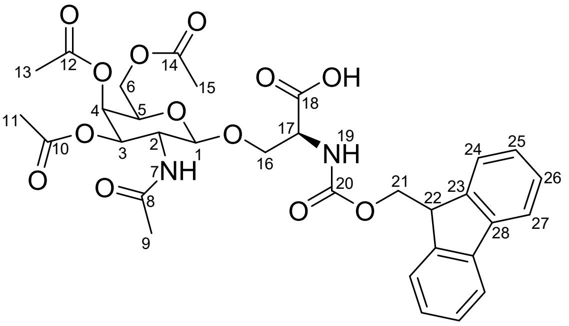

Followed demethylation procedure starting from Fmoc-Ser[GalNAc(Ac)$_3$-β-D]-OMe (200 mg, 0.298 mmol) and refluxed for 4 hours. Fmoc-Ser[GalNAc(Ac)$_3$-β-D]-OH was isolated (167 mg, 85%) as a colourless oil.

IR (neat): v_{max}/cm^{-1} 3334.7 (N-H & O-H, m, broad), 2968.3 (C-H, w), 1741.4 (C=O, s), 1662.0 (C=C, s). **HRMS:** (ESI)+ calcd for $C_{32}H_{36}N_2O_{13}$ $[M+H]^+$: 657.2290, found 657.2285. **$[\alpha]_D$** = +234° (*c* = 0.1, DMSO) **^{1}H NMR** (700 MHz, MeOD) δ 7.80 (d, *J* = 7.5 Hz, 2H, H^{27}), 7.69 (t, *J* = 8.2 Hz, 2H, H^{24}), 7.40 (td, *J* = 7.4, 2.6 Hz, 2H, H^{26}), 7.33 (qd, *J* = 7.9, 1.1 Hz, 2H, H^{25}), 5.33 (d, *J* = 2.4 Hz, 1H, H^{4}), 5.07 (dd, *J* = 11.3, 3.3 Hz, 1H, H^{3}), 4.63 (d, *J* = 8.5 Hz, 1H, H^{1}), 4.44 (dd, *J* = 10.6, 6.8 Hz, 1H, H^{21}), 4.39 (t, *J* = 4.7 Hz, 1H, H^{5}), 4.33 (dd, *J* = 10.6, 7.0 Hz, 1H, H^{21}), 4.25 (t, *J* = 6.8 Hz, 1H, H^{22}), 4.18 – 4.09 (m, 3H, $H^{6,16}$), 4.06 (dd, *J* = 11.2, 8.5 Hz, 1H, H^{2}), 3.99 (t, *J* = 7.2 Hz, 1H, H^{17}), 3.93 (dd, *J* = 10.5, 4.1 Hz, 1H, H^{6}), 2.12 (s, 3H, H^{15}), 2.01 (s, 3H, H^{13}), 1.95 (s, 3H, H^{11}), 1.88 (s, 3H, H^{9}). **^{13}C NMR** (176 MHz, MeOD) δ 174.0 (C^{8}), 173.0 (C^{18}), 172.2 (C^{12}), 172.2 (C^{14}), 171.8 (C^{10}), 158.4 (C^{20}), 145.3/145.2 ($C^{28/28'}$), 142.6/142.6 ($C^{23/23'}$), 128.8 (C^{26}), 128.2 (C^{25}), 126.3/126.2 ($C^{24/24'}$), 121.0 (C^{27}), 102.5 (C^{1}), 71.9 (C^{17}), 71.9 (C^{3}), 69.9 (C^{6}), 68.1 (C^{4}), 68.1 (C^{21}), 62.6 (C^{16}), 55.5 (C^{5}), 51.5 (C^{2}), 48.3 (C^{22}), 22.9 (C^{9}), 20.6 ($C^{11/13/15}$), 20.5 ($C^{11/13/15}$), 20.5 ($C^{11/13/15}$).

Fmoc-Thr[GlcNAc(Ac)$_3$-β-D]-OH β12

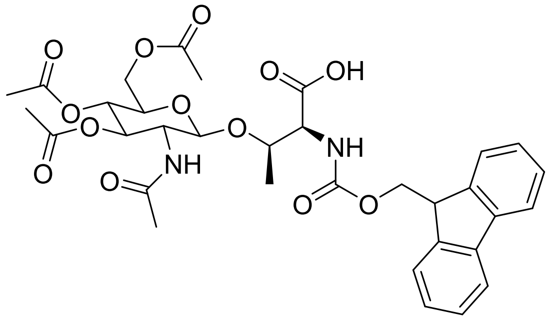

Followed demethylation procedure starting from Fmoc-Thr[GlcNAc(Ac)$_3$-β-D]-OMe (27 mg, 0.039 mmol). Fmoc-Thr[GlcNAc(Ac)$_3$-β-D]-OH was isolated (15 mg, 58%) as a colourless oil.

Data matched literature reports. [4]

Fmoc-Ser[GlcNAc(Ac)$_3$-β-D]-OH β13

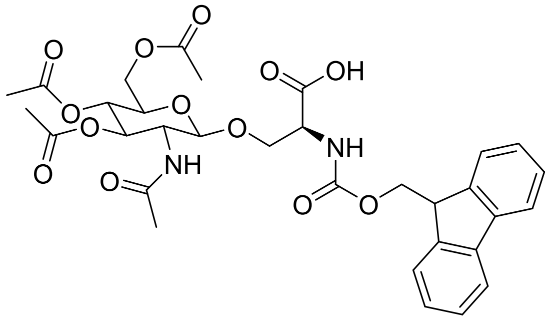

Followed demethylation procedure starting from Fmoc-Ser[GlcNAc(Ac)$_3$-β-D]-OMe (26 mg, 0.039 mmol). Fmoc-Ser[GlcNAc(Ac)$_3$-β-D]-OH was isolated (17 mg, 66%) as a colourless oil.

Data matched literature reports. [6]

Anomerisation time course experiment

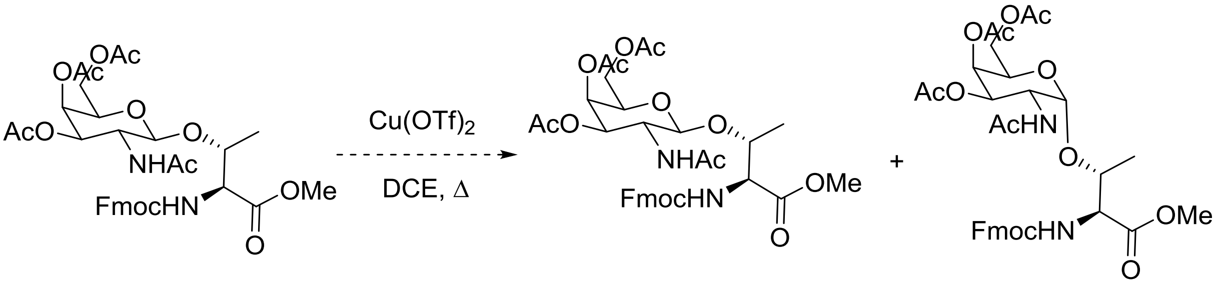

To a 2-neck 25mL rbf, under nitrogen, was added Fmoc-Thr[GalNAc(Ac)$_3$-β-D]-OMe β**7** and Fmoc-Thr[GalNAc(Ac)$_3$-β-D]-OH α**7** (80 mg, 0.117 mmol, approx. 7:1 β**7**/α**7** ratio), Cu(OTf)$_2$ (40 mg, 0.117 mmol) and DCE (2.27 mL). The reaction mixture was degassed (x3) and heated to reflux. The reaction was initially monitored by LCMS, taking a timepoint every 30 mins, starting from t = 0 up to t = 330, after which the reaction was left overnight with a final timepoint at t = 1380 mins. Each LCMS sample was made up of 2.8 µL of crude reaction mixture and 1 mL of MeCN. The UV absorbance at 280 nm was used to quantify the species present based on peak area:

	Peak retention time (min)	Region for area calculation (min)
Fmoc-Thr-OMe **6**	3.86	3.78-3.94
Fmoc-Thr[GalNAc(Ac)3-β-D]-OMe β**7**	4.04	3.97-4.11
Fmoc-Thr[GalNAc(Ac)3-α-D]-OMe α**7**	4.22	4.15-4.29

The areas for **6**, β**7** and α**7** were divided by the sum of the areas at each timepoint, to normalise between samples, and multiplied by 100 to calculate the percentage of each species in the reaction at the given time. These values are shown below (Table SX)and was plotted as a function of time to produce Figure 2 of the main text.

	Percentage area		
Time (min)	**6**	β**7**	α**7**
0	3.95	82.23	13.82
30	13.06	76.28	10.66
60	24.92	61.99	13.10
90	34.04	55.10	10.86

120	39.38	48.25	12.37
150	43.95	43.24	12.81
180	51.67	36.98	11.35
210	55.44	32.91	11.65
240	57.17	30.81	12.02
270	55.97	30.19	13.84
300	58.66	27.61	13.73
330	58.85	25.64	15.51
1380	48.20	11.17	40.63

Calculation of Synthesis cost

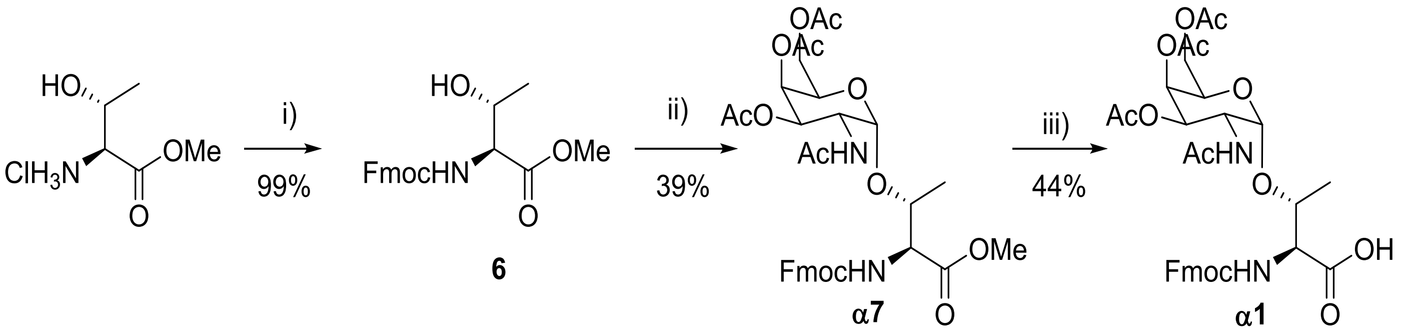

i) Cl.H_3N-Thr-OMe (1 equiv.), Fmoc-Cl (1 equiv.); 1,4-dioxane/20% $NaHCO_3$(aq) (1:2) 0.66 M
ii) Fmoc-Thr-OMe (5 equiv.), GalNAc(Ac3)-β-OAc (1 equiv.), $Cu(OTf)_2$ (1 equiv.); 1,4-dichloroethane 51 mM
iii) Fmoc-Thr(GalNAc(Ac)3-α-D]-OMe (1 equiv.), LiI (6 equiv.); EtOAc 10 mM

To calculate the total cost of synthesis Fmoc-Thr[GalNAc(Ac)3-α-D]-OH α**1** the yields across steps i)-iii) were used to calculate the amount of each commercially available compound required to produce the target quantity of 100 mg.

As an example in the demethylation reaction, step iii); 100 mg of Fmoc-Thr[GalNAc(Ac)3-α-D]-OH α**1** (0.149 mmol) can be expected from a reaction with input of 243.1 mg Fmoc-Thr[GalNAc(Ac)3-α-D]-OMe α**7** (0.355 mmol) as the expected yield is 44%. A reaction on this scale would require:

- Fmoc-Thr[GalNAc(Ac)3-α-D]-OMe α**7**: 243.1 mg (0.355 mmol, 1 equiv.) produced from step ii). A similar calculation was used to determine the input reactants required to produce this quantity from step ii) and likewise for step i) to determine the quantity required of all commercial reagents.
- LiI: 285.3 mg (2.131 mmol, 6 equiv.) of LiI. This is purchased and would cost £0.229.
- EtOAc: 35.5 mL (10 mM reactant concentration based on α**7**). This is purchased and would cost £1.302.

The sum of the cost of the required reagents was calculated to give the total cost.

Step	Reagents	rMM	mass (mg)	mmol	Equiv.	cost (£)	yield(%)
	Fmoc-Thr[GalNAc(Ac)$_3$-α-D]-OH	670.24	100.0	0.149	N/A	Made in iii)	44
iii)	Fmoc-Thr[GalNAc(Ac)$_3$-α-D]-OMe	684.25	243.1	0.339	1	Made in ii)	39
	LiI	133.84	285.3	2.035	6	0.229	
ii)	GalNAc(Ac$_3$)-β-OAc	389.36	354.7	0.869	1	0.704	
	Cu(OTf)$_2$	361.68	329.4	0.869	1	0.252	
	Fmoc-Thr-OMe	355.14	1617.4	4.347	5	Made in i)	99
i)	Cl.H3N-Thr-OMe	169.60	780.2	4.391	1	1.013	
	Fmoc-Cl	258.70	1190.1	4.391	1	0.727	
	**Solvents**		**Volume (mL)**	**mmol**	**concentration (mM)**	**cost (£)**	
**iii)**	EtOAc		33.9	0.339	10	1.302	
**ii)**	1,2-Dichloroethane		17.0	0.869	51	1.454	
**i)**	1,4-dioxane		2.2	4.391	2000*	0.425	
					**Total cost (£):**	**6.105**	

Table S2: Cost calculation for Fmoc-Thr[GalNAc(Ac)$_3$-α-D]-OH **α1.** *reaction uses mixed solvent system, this would be the concentration with 1,4-dioxane only

Cheapest commercial alternative: BLD Pharm BD131423, 250 mg for £1049 (Table S1). 100 mg cost = £419.60. Our cost is 1.46% of this.

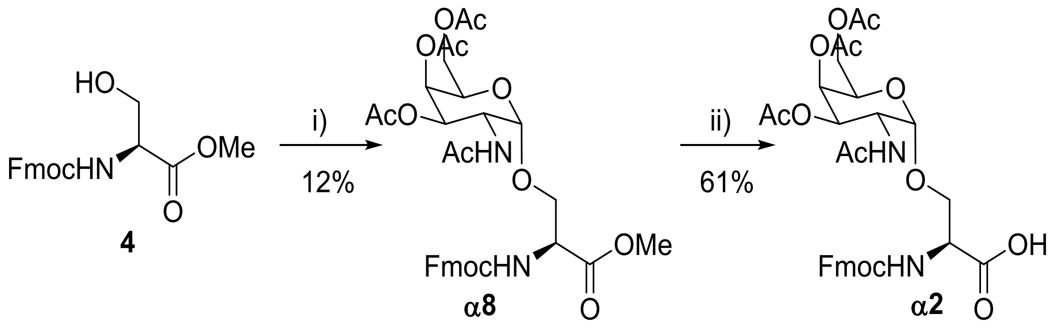

i) Fmoc-Ser-OMe (1 equiv.), GalNAc(Ac3)-β-OAc (1 equiv.), $Cu(OTf)_2$ (1 equiv.), 1,2-dichloroethane 51 mM
ii) Fmoc-Ser(GalNAc(Ac)3-α-D]-OMe (1 equiv.), LiI (6 equiv.), EtOAc 10 mM

Step	Reagents	rMM	mass (mg)	mmol	Equiv.	cost (£)	yield(%)
	Fmoc-Ser[GalNAc(Ac)$_3$-α-D]-OH	656.22	100.0	0.149	N/A	Made in ii)	61
ii)	Fmoc-Ser[GalNAc(Ac)$_3$-α-D]-OMe	670.24	167.4	0.250	1	Made in i)	12
	LiI	133.84	200.6	1.499	6	0.169	
i)	GalNAc(Ac$_3$)-β-OAc	389.36	810.6	2.082	1	1.686	
	$Cu(OTf)_2$	361.68	329.4	2.082	1	0.602	
	Fmoc-Ser-OMe	355.14	1617.4	2.082	1	0.739	
	**Solvents**		**Volume (mL)**	**mmol**	**concentration (mM)**	**cost (£)**	
ii)	EtOAc		24.9	0.250	10	0.959	
i)	1,2-Dichloroethane		40.8	2.082	51	3.482	
					**Total cost (£):**	**7.637**	

Table S3: Cost calculation for Fmoc-Ser[GalNAc(Ac)$_3$-α-D]-OH **α2**

Cheapest commercial alternative: Key Organics BS-49043, 250 mg for £552 (Table S1). 100 mg cost = £220.80. Our cost is 3.46% of this.

Reagents	CAS	Supplier	Amount (g)	Price/£	Link	Date
Fmoc-Ser-OMe	82911-78-2	Doug Discovery	25	25	F234541	07/03/2025
Cl.H3N-Thr-OMe	39994-75-7	Doug Discovery	25	34	M02985	07/03/2025
Fmoc-Cl	28920-43-6	Doug Discovery	25	16	F022072	07/03/2025
GalNAc(Ac3)-β-OAc	3006-60-8	Doug Discovery	25	52	F238299	27/03/2025
GlcNAc(Ac3)-β-OAc	7772-79-4	Doug Discovery	25	26	F239393	27/03/2025
Cu(OTf)2	34946-82-2	Doug Discovery	25	20	F012761	07/03/2025
LiI	10377-51-2	Doug Discovery	25	21	F493928	07/03/2025
**Solvents**	**CAS**	**Supplier**	**Amount (L)**	**Price/£**	**Link**	**Date**
1,2-Dichloroethane	107-06-2	Sigma Aldrich	1	85.3	284505	07/03/2025
EtOAc	141-78-6	Sigma Aldrich	2.5	96	33211m	07/03/2025
1,4-dioxane	123-91-1	Doug Discovery	0.1034	20	F044719	07/03/2025

Table S4: Cost of reagents used.

NMR Spectra

Fmoc Thr-OMe 6

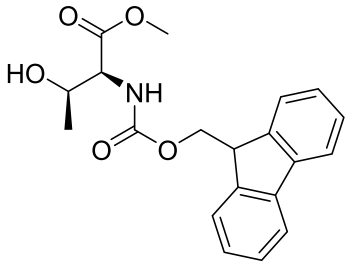

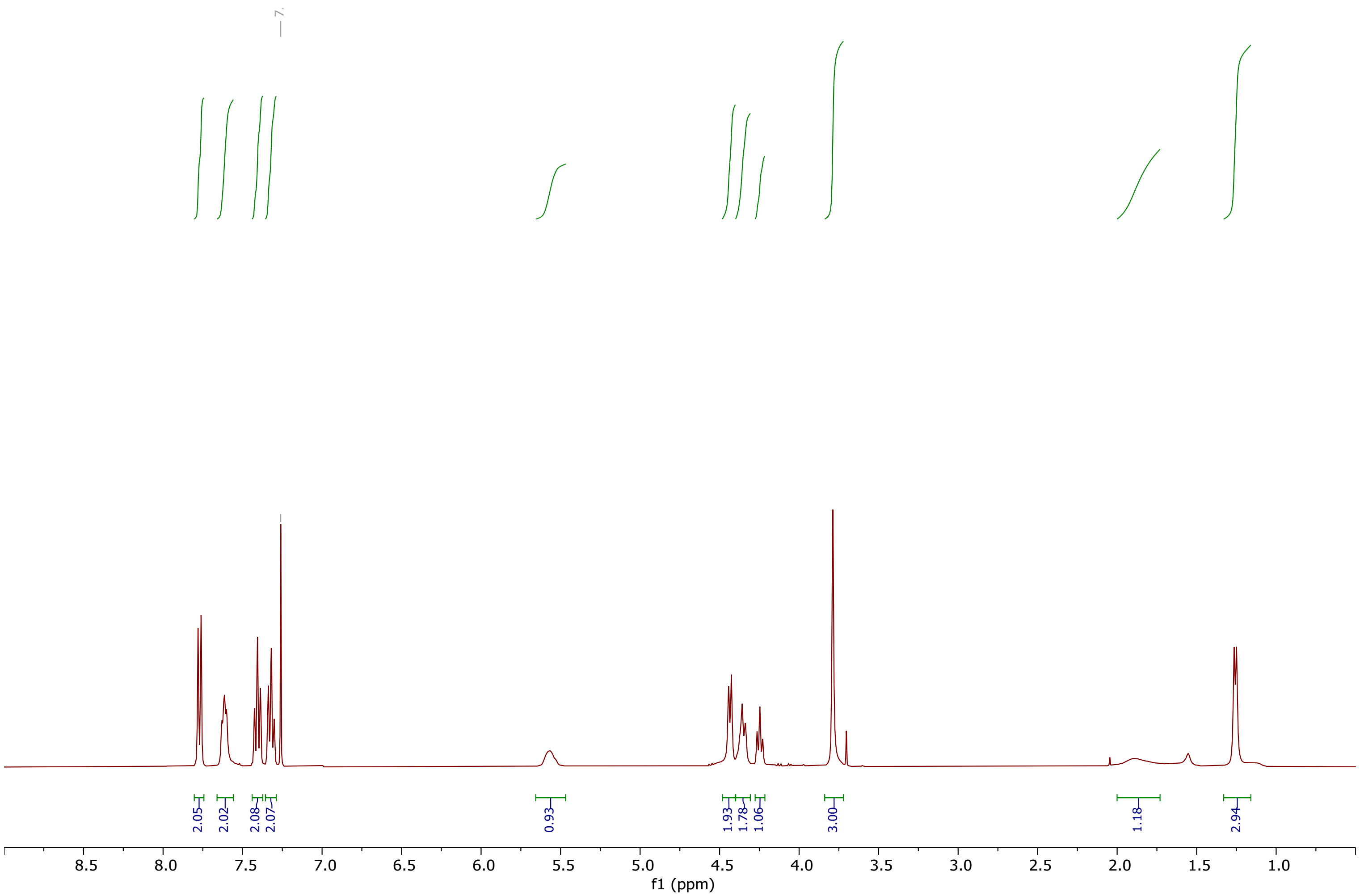

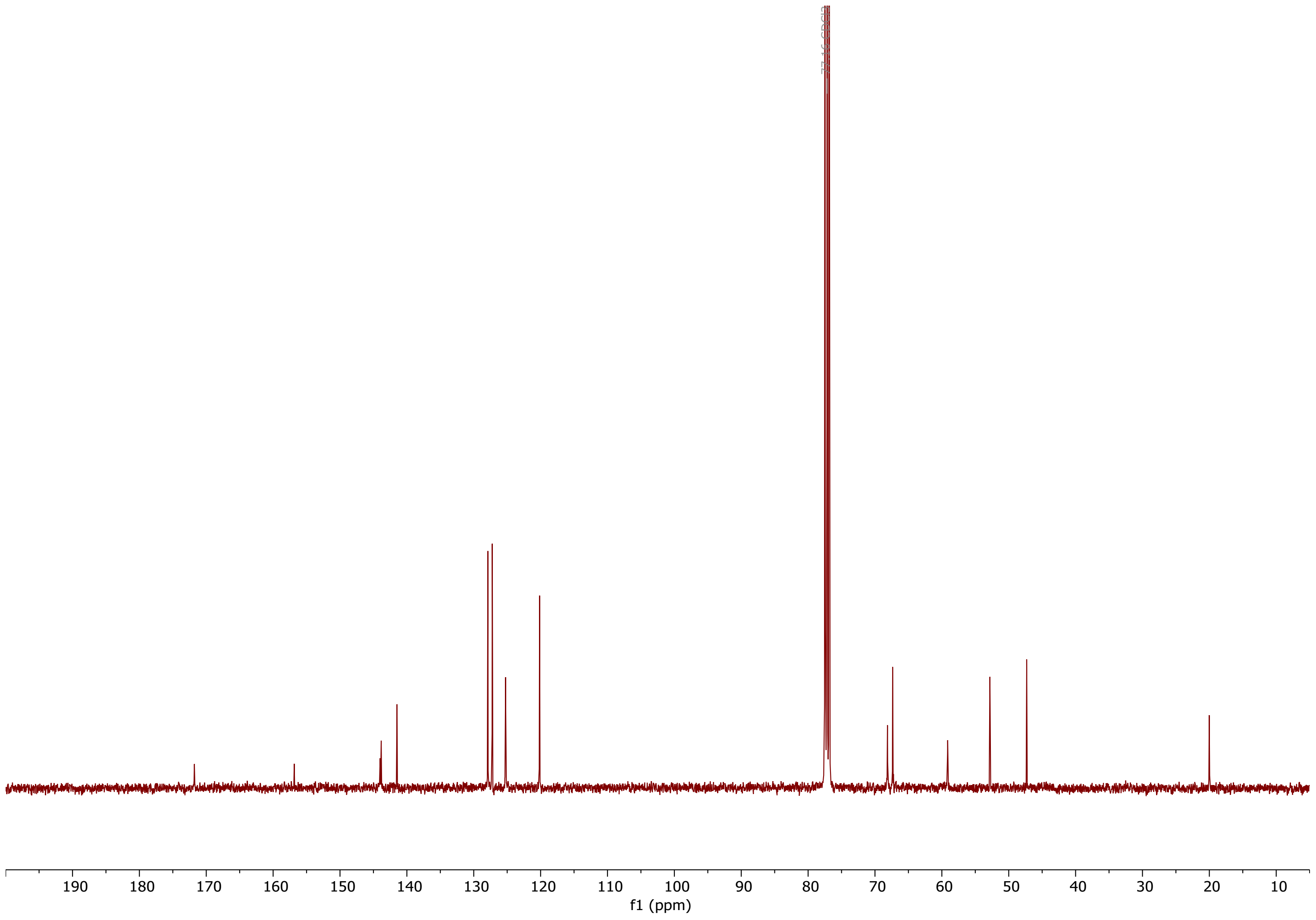
190
180
170
160
150
140
130
120
110
100
90
80
70
60
50
40
30
20
10
f1 (ppm)

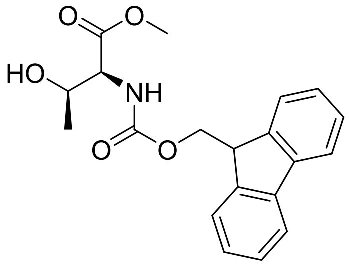
HO
O
O
NH
O
O

Fmoc-Thr[GalNAc(Ac)3-α-D]-OMe α7

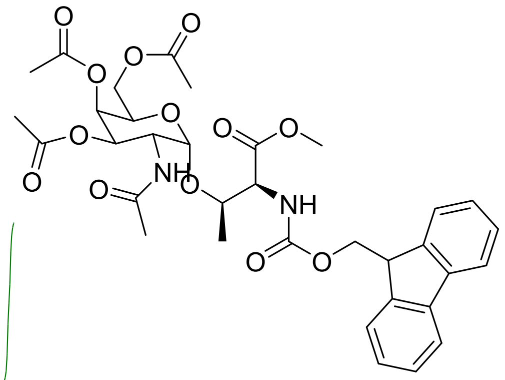

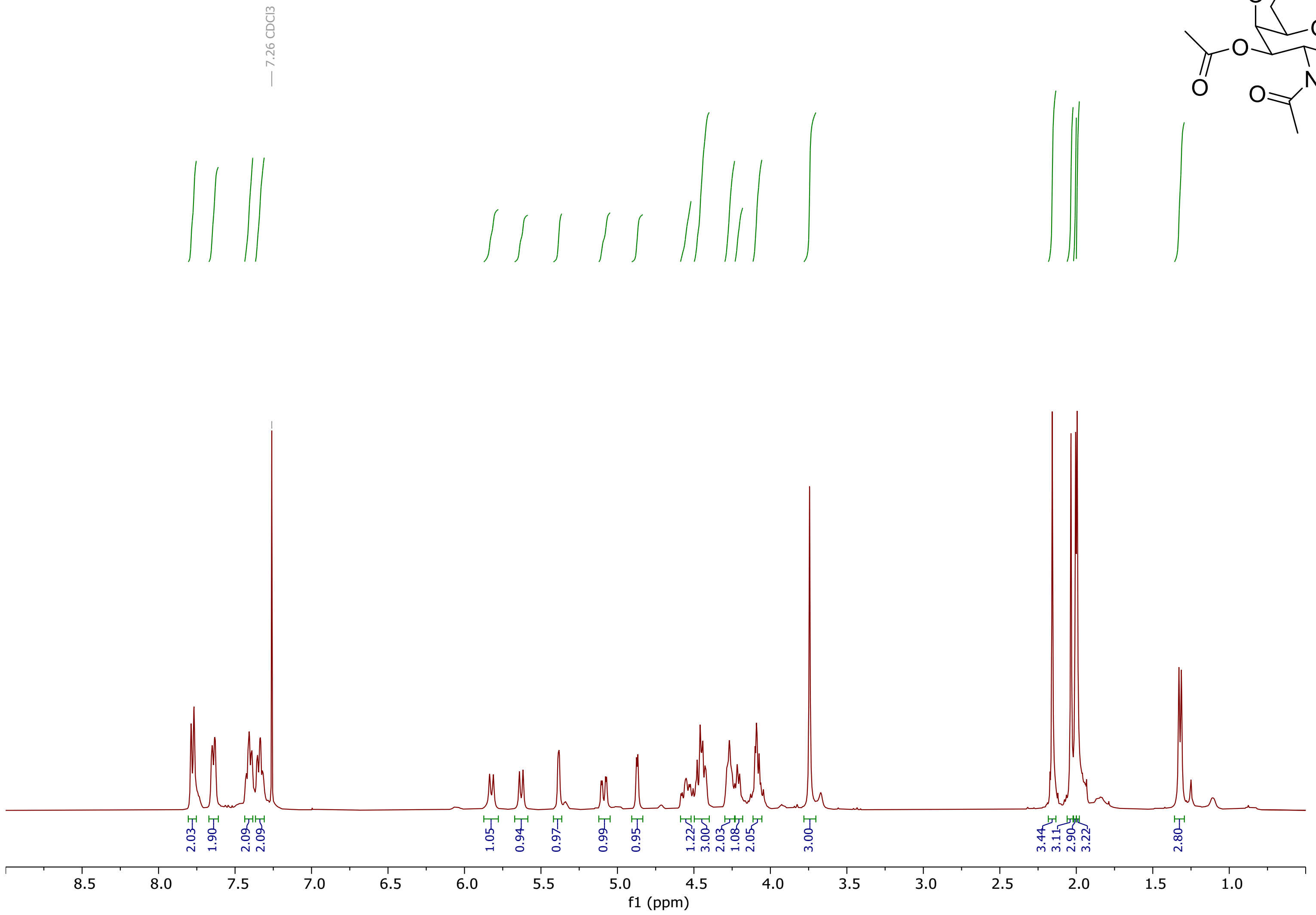

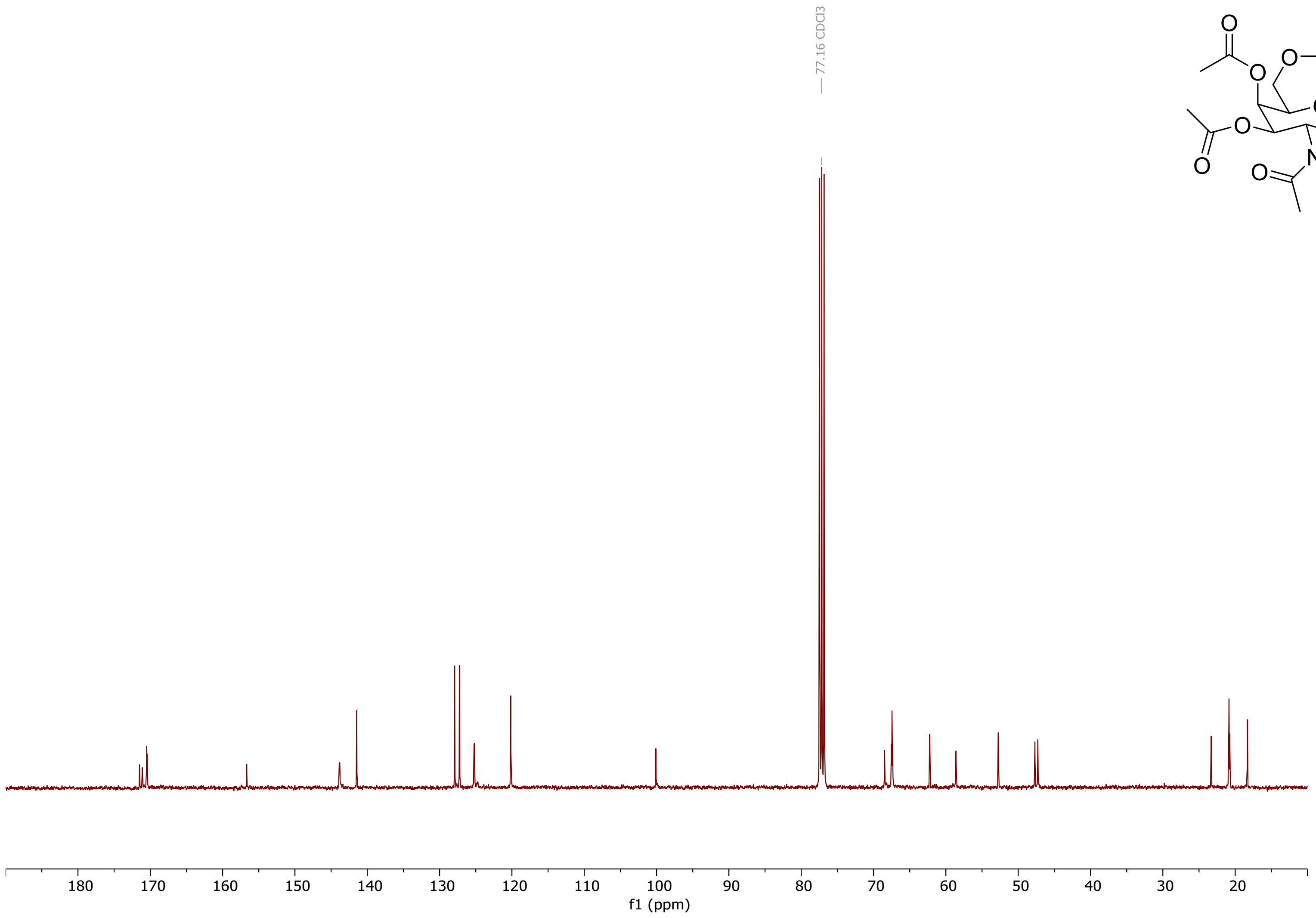

77.16 CDCl3
180
170
160
150
140
130
120
110
100
90
80
70
60
50
40
30
20
f1 (ppm)

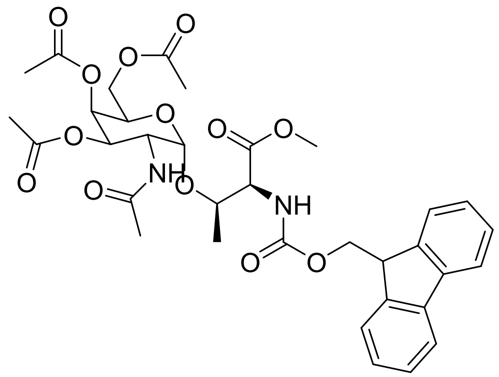

Fmoc-Thr[GalNAc(Ac)3-β-D]-OMe β7

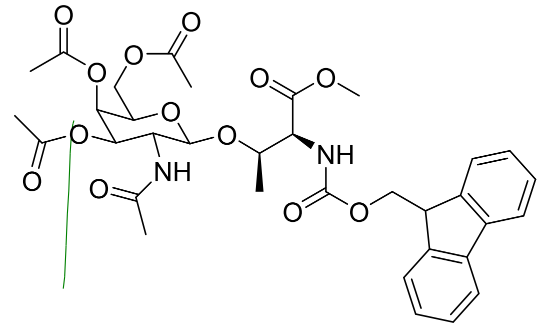

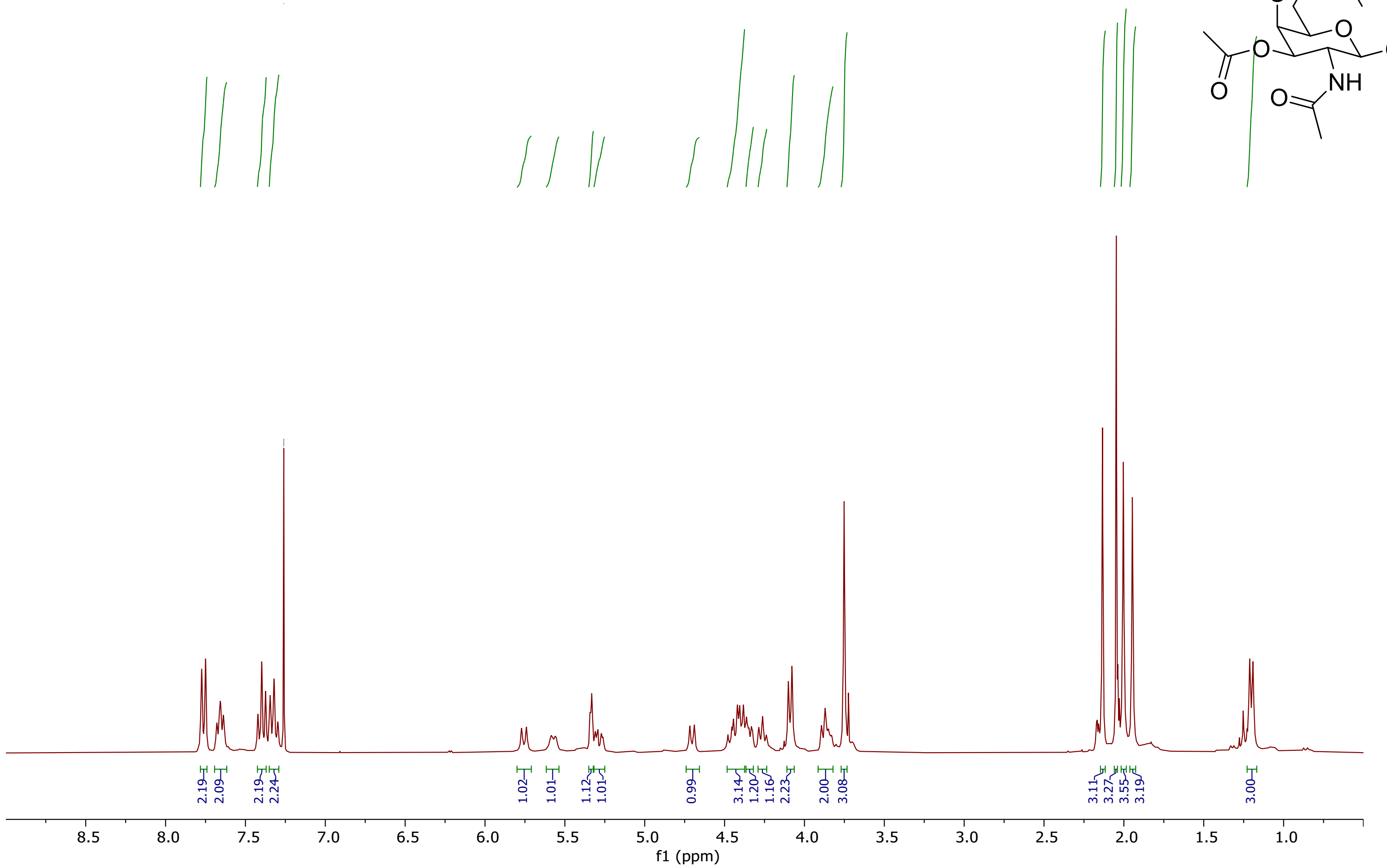

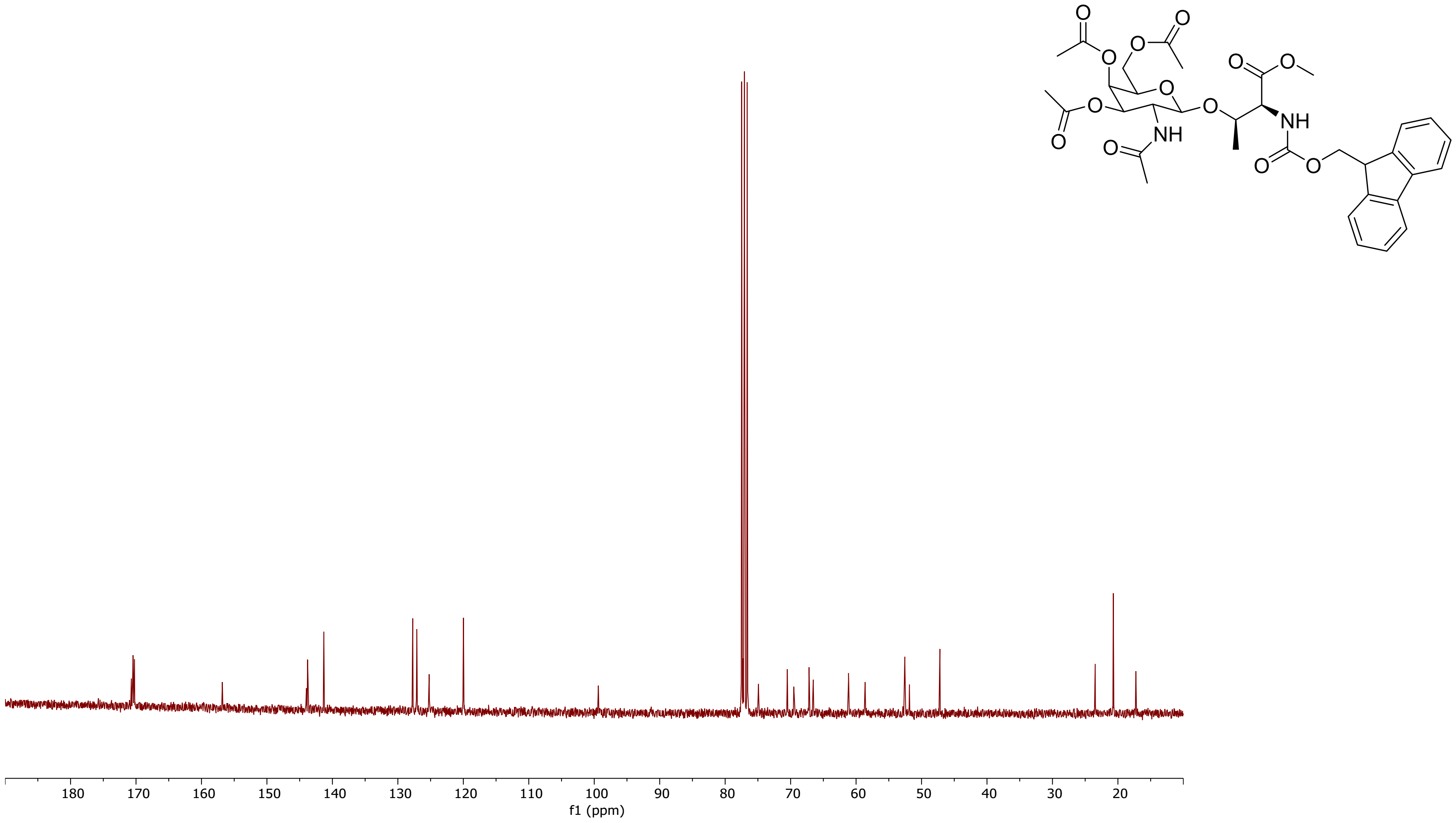
180 170 160 150 140 130 120 110 100 90 80 70 60 50 40 30 20
f1 (ppm)

Fmoc-Ser[GalNAc(Ac)3-α-D]-OMe α8

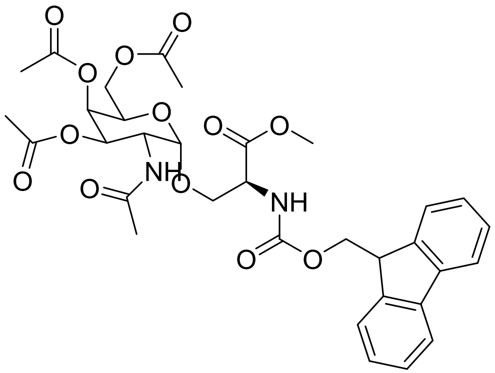

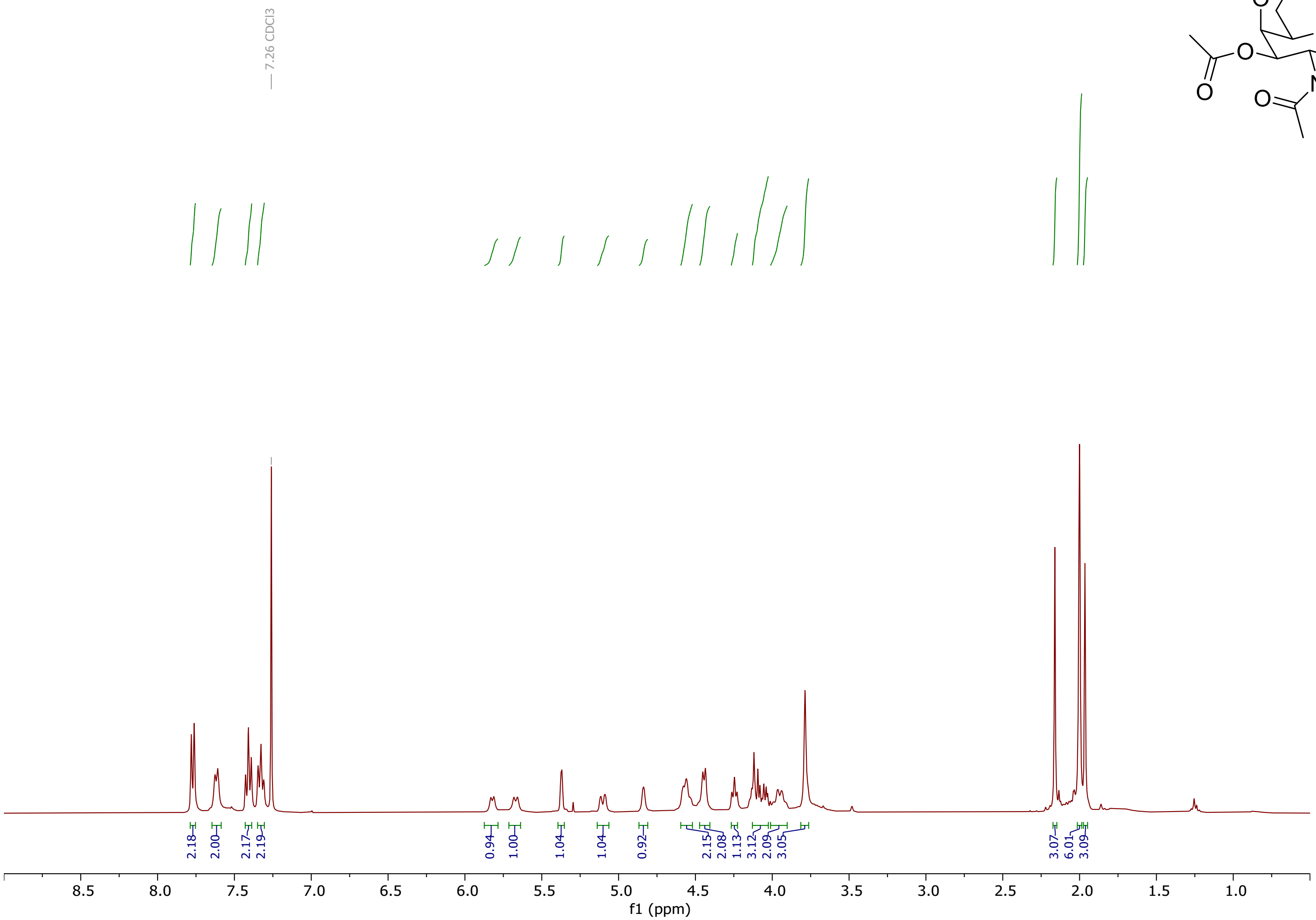

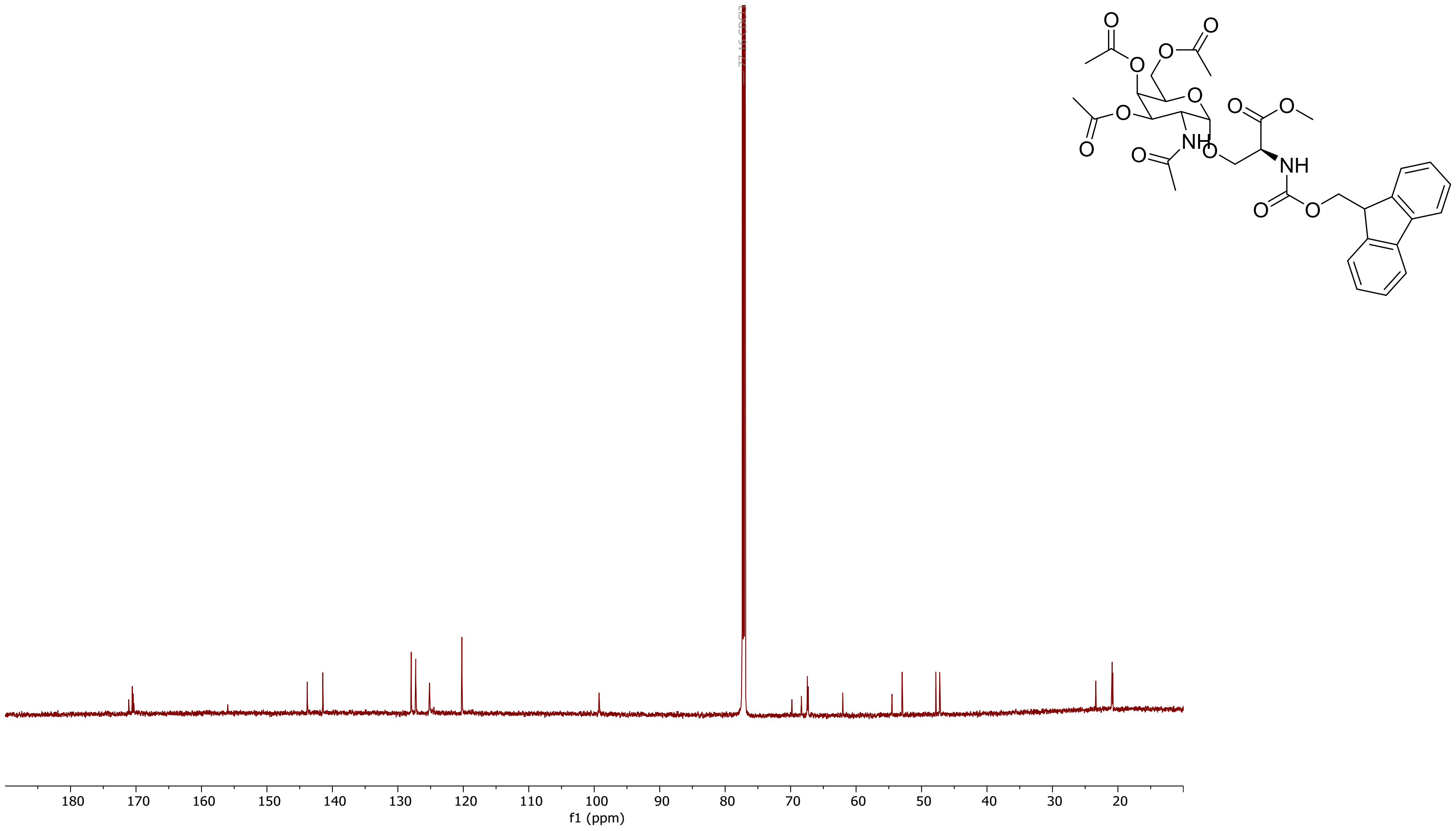
77.16 CDCl3
180 170 160 150 140 130 120 110 100 90 80 70 60 50 40 30 20
f1 (ppm)

Fmoc-Ser[GalNAc(Ac)3-β-D]-OMe β8

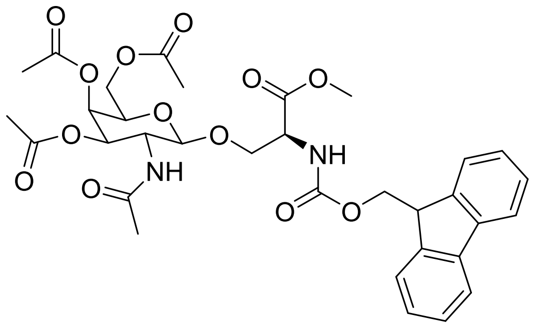

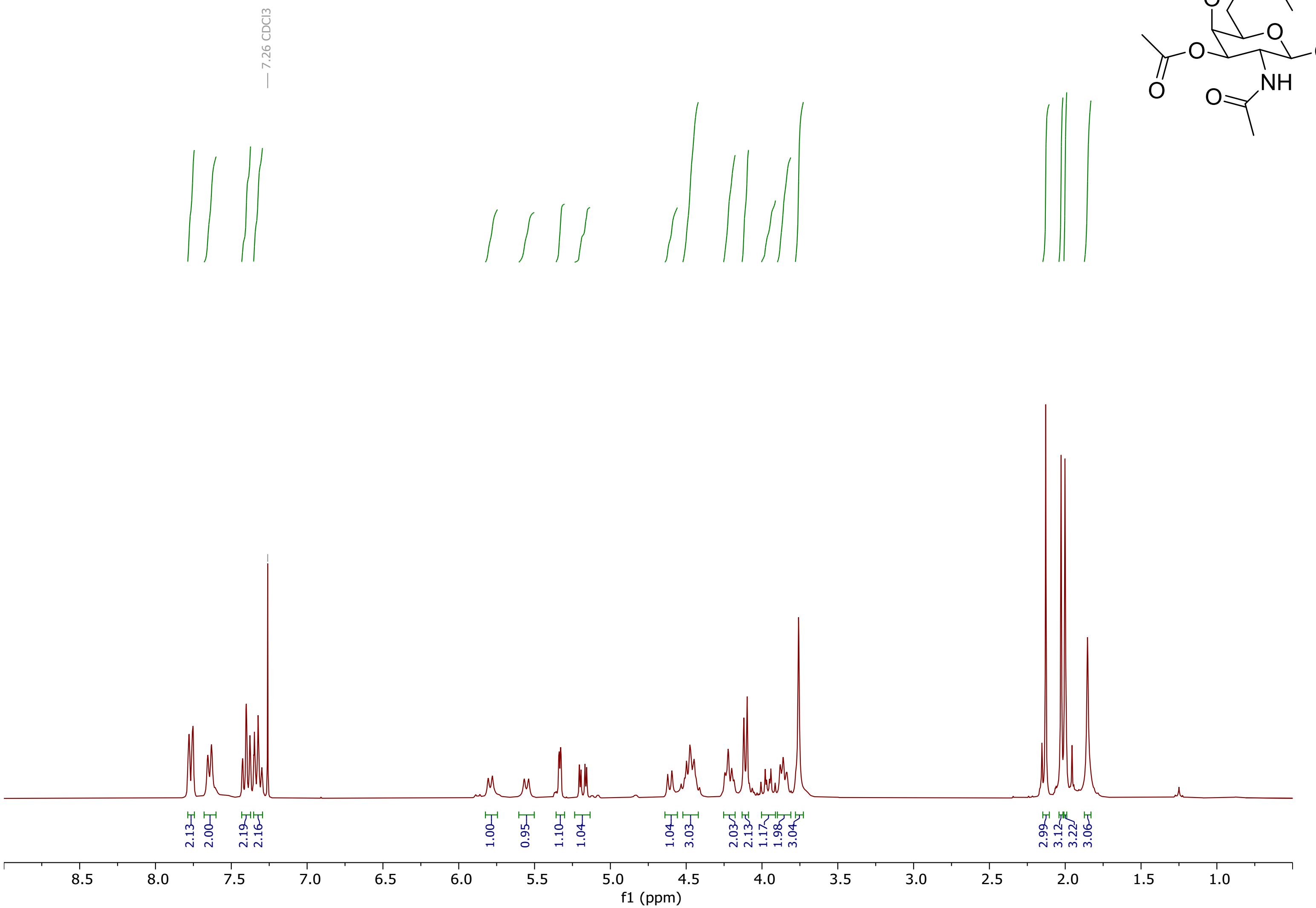

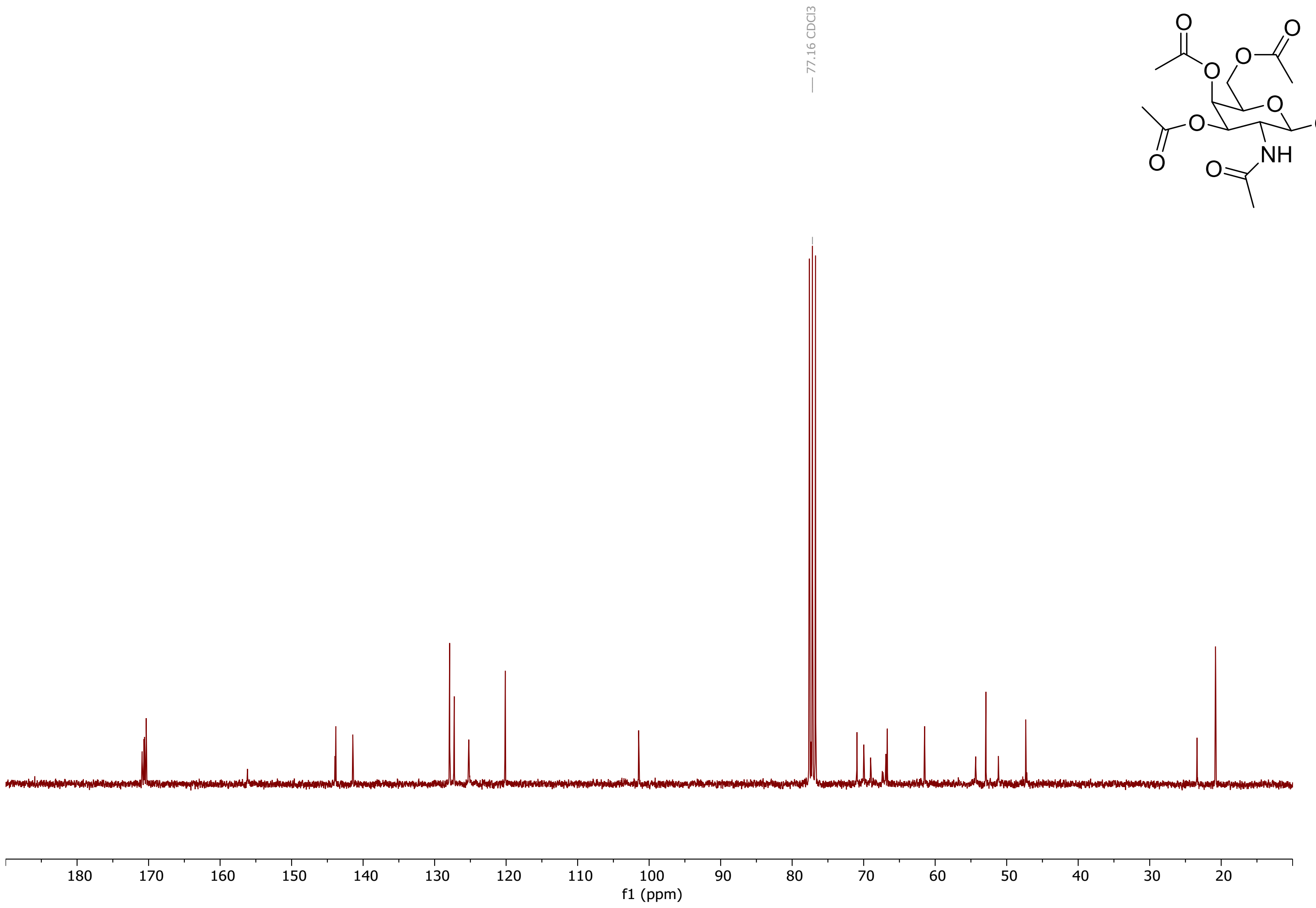
77.16 CDCl3
180
170
160
150
140
130
120
110
100
90
80
70
60
50
40
30
20
f1 (ppm)

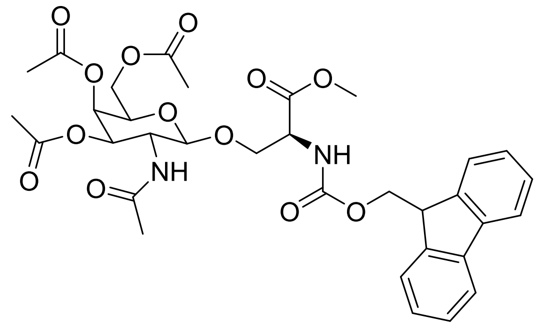
O
NH
NH

Fmoc-Thr[GlcNAc(Ac)3-α-D]-OMe α9

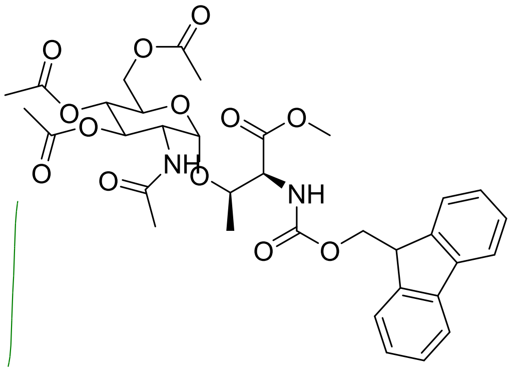

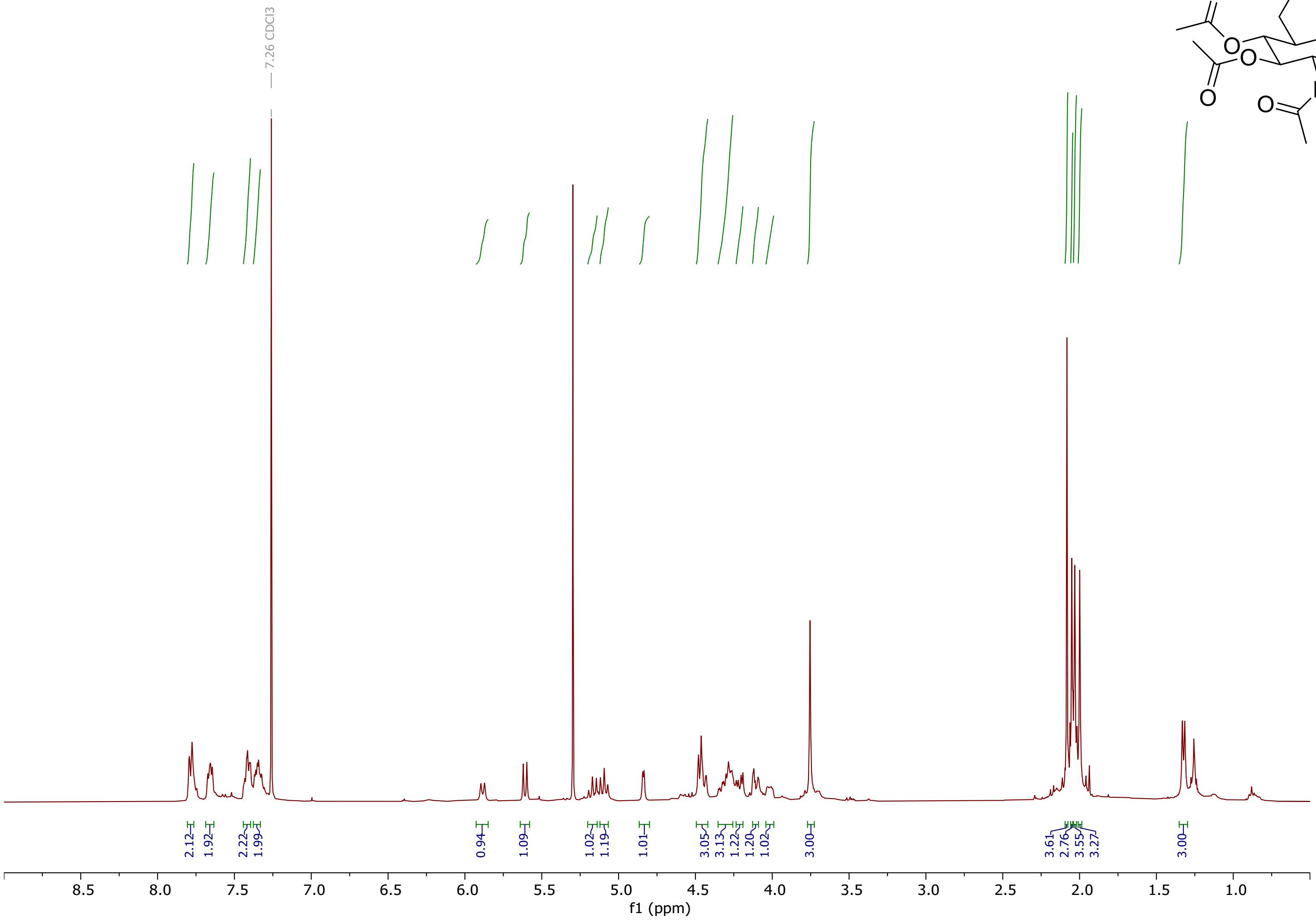

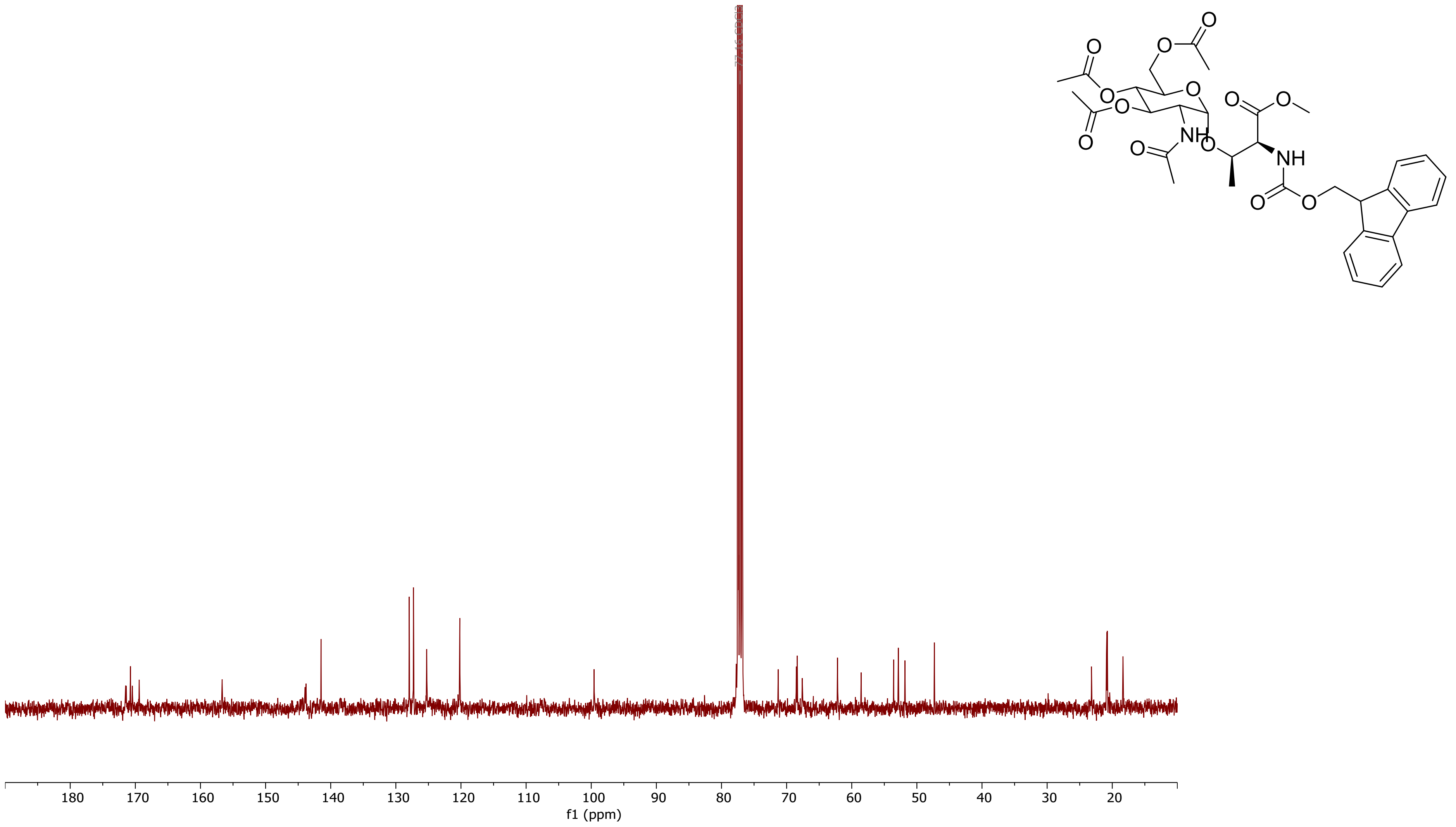
180
170
160
150
140
130
120
110
100
90
80
70
60
50
40
30
20
f1 (ppm)
O
NH
O

Fmoc-Thr[GlcNAc(Ac)3-β-D]-OMe β9

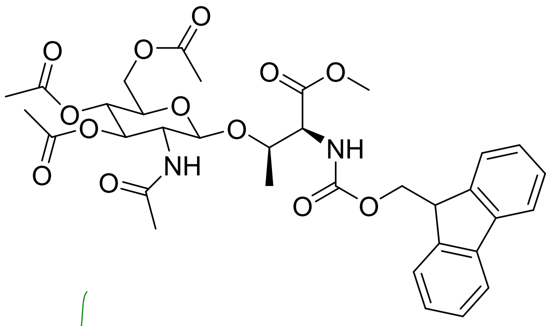

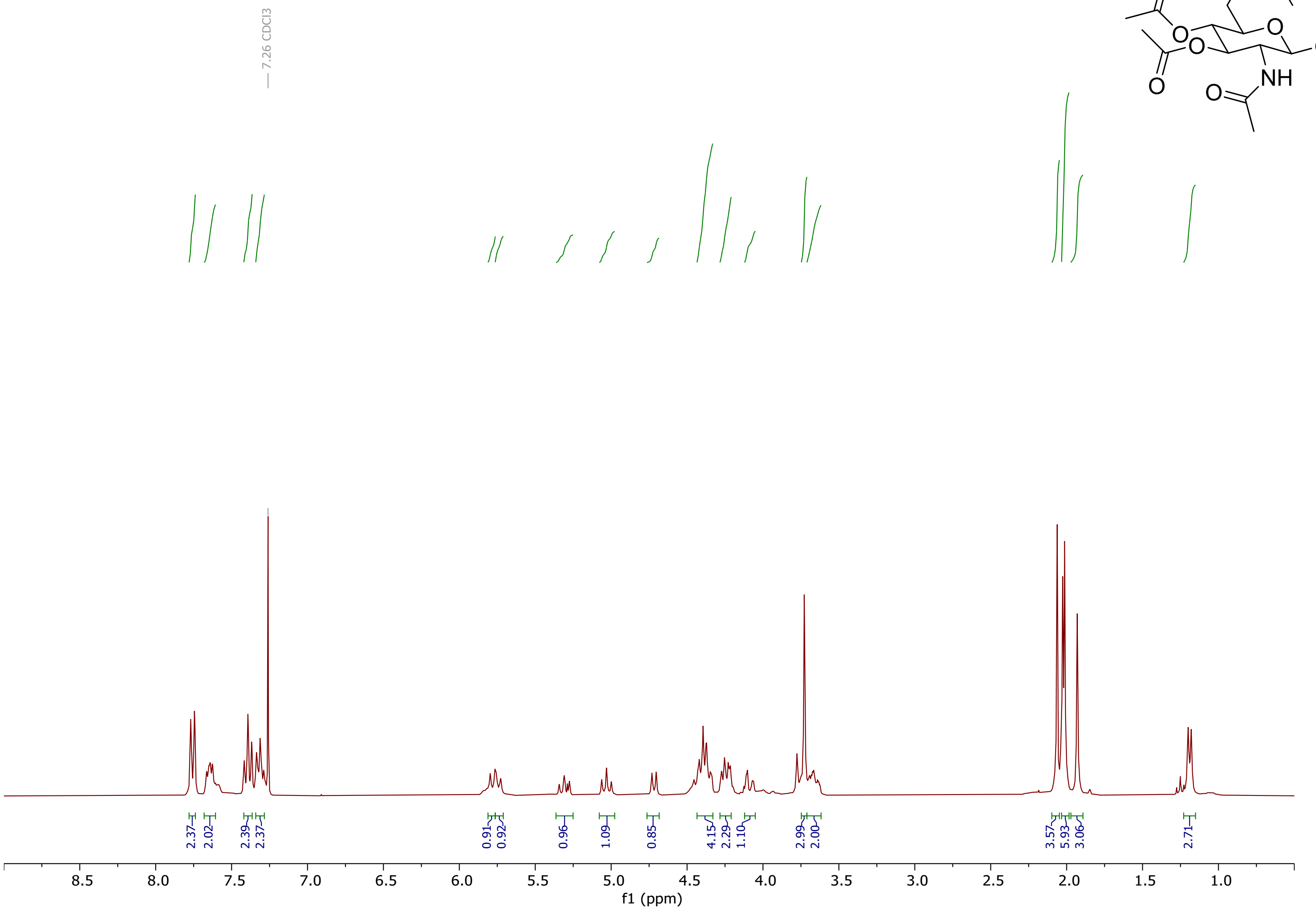

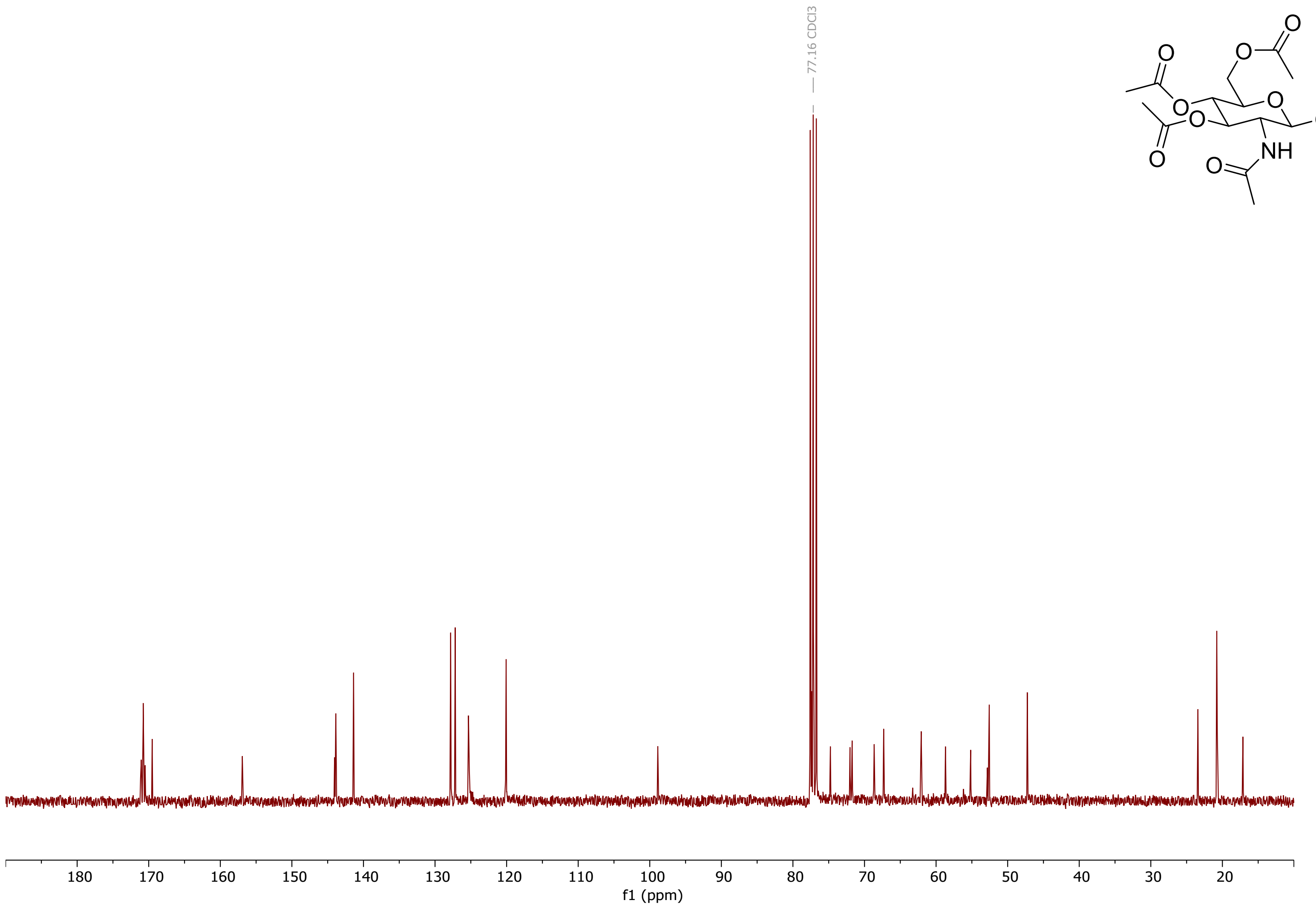

77.16 CDCl3
180
170
160
150
140
130
120
110
100
90
80
70
60
50
40
30
20
f1 (ppm)

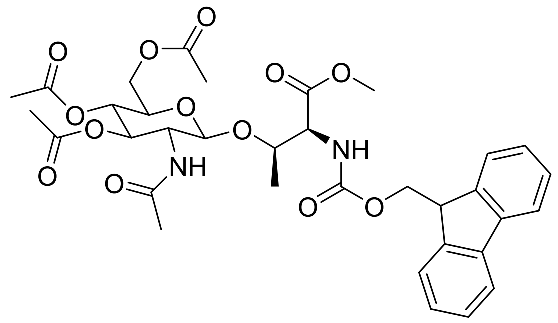

Fmoc-Ser[GlcNAc(Ac)3-β-D]-OMe β10

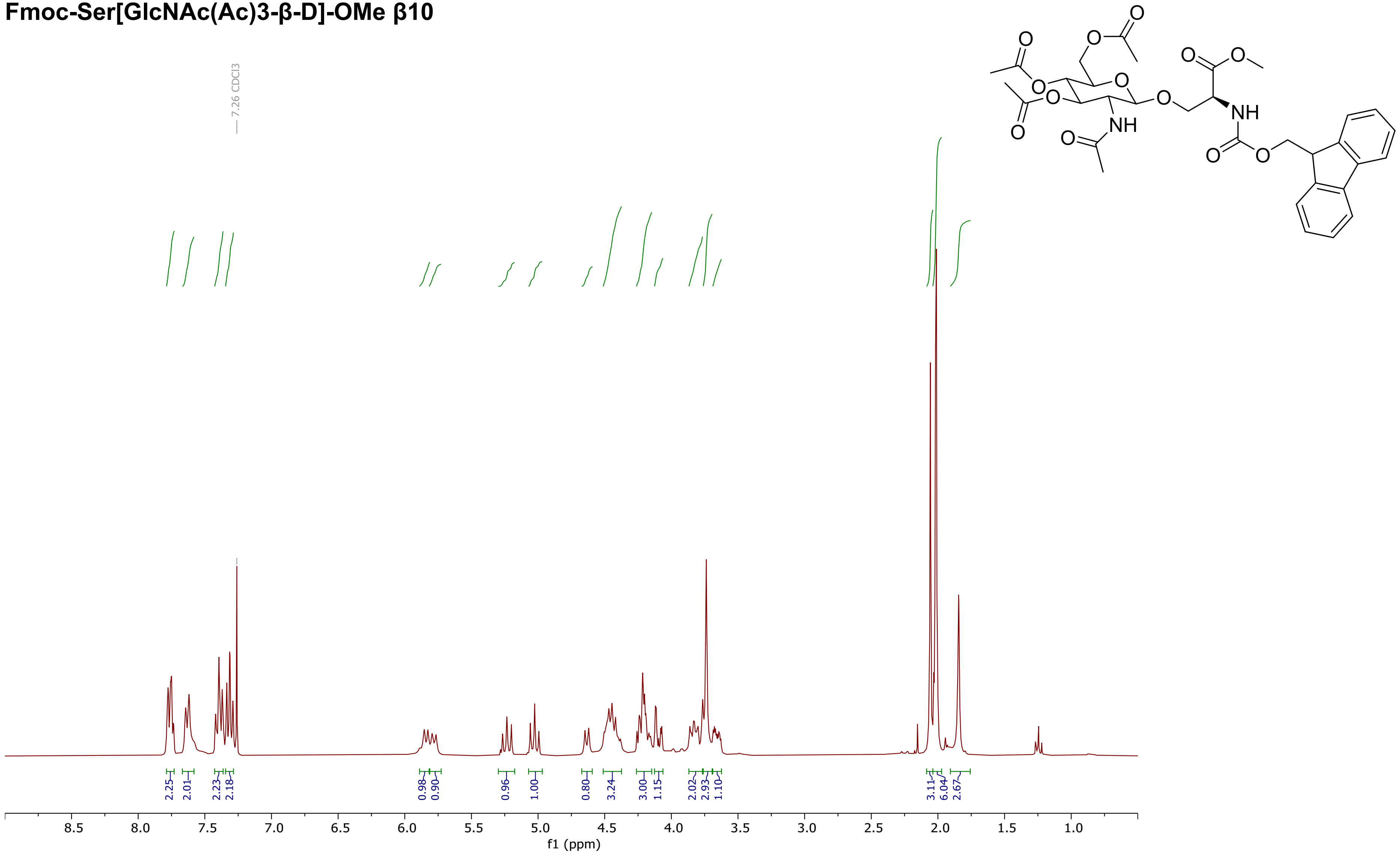

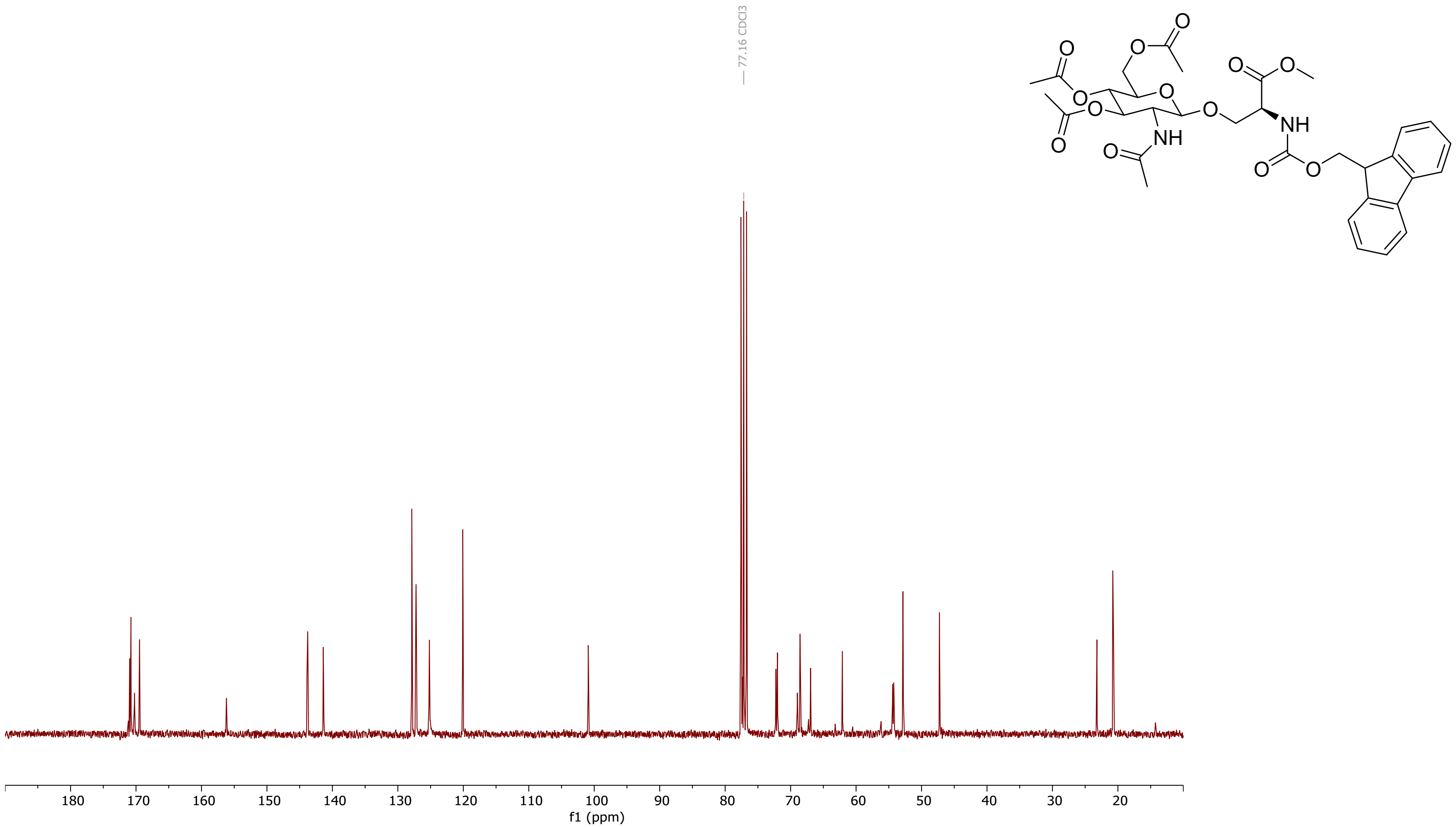
77.16 CDCl3
180 170 160 150 140 130 120 110 100 90 80 70 60 50 40 30 20
f1 (ppm)
O
O
O
O
O
O
O
O
O
O
NH
O
NH
O
O

Fmoc-Thr[GalNAc(Ac)3-α-D]-OH α1

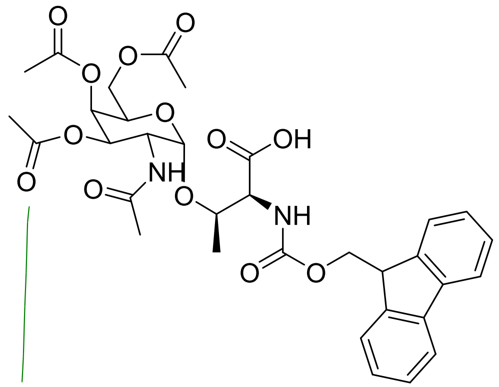

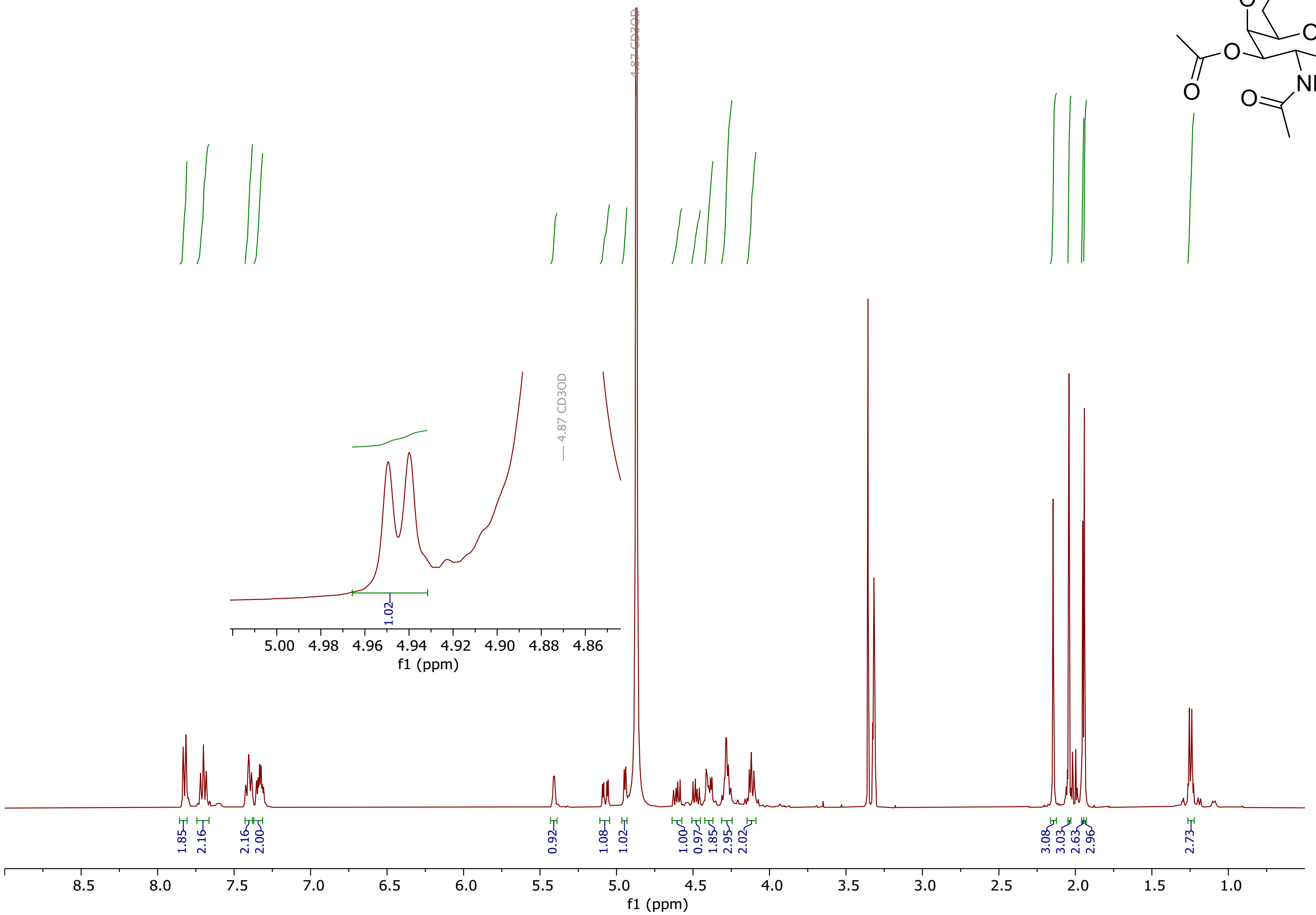

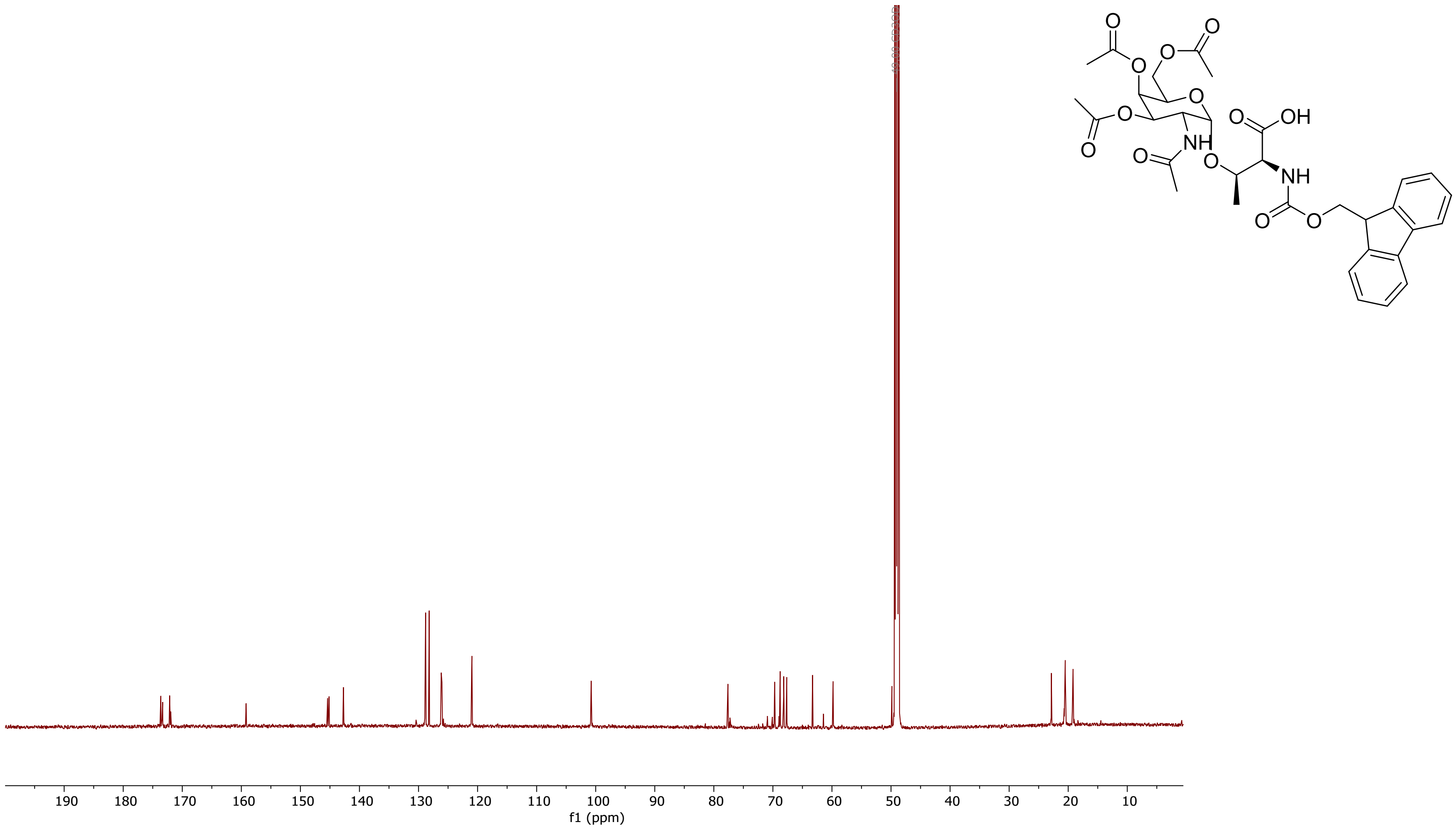
190
180
170
160
150
140
130
120
110
100
90
80
70
60
50
40
30
20
10
f1 (ppm)
O
OH
NH
O
O

Fmoc-Thr[GalNAc(Ac)3-β-D]-OH β1

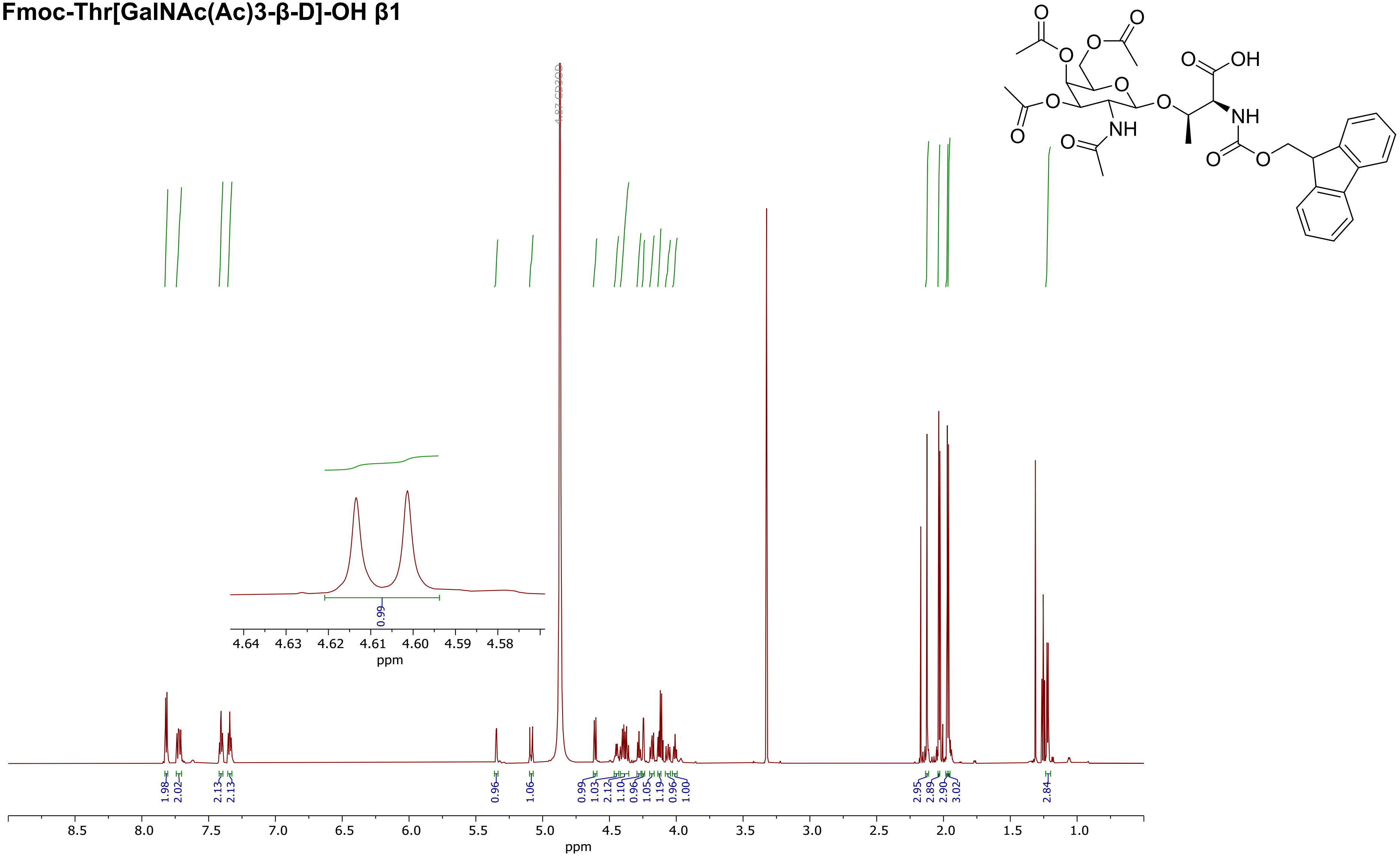

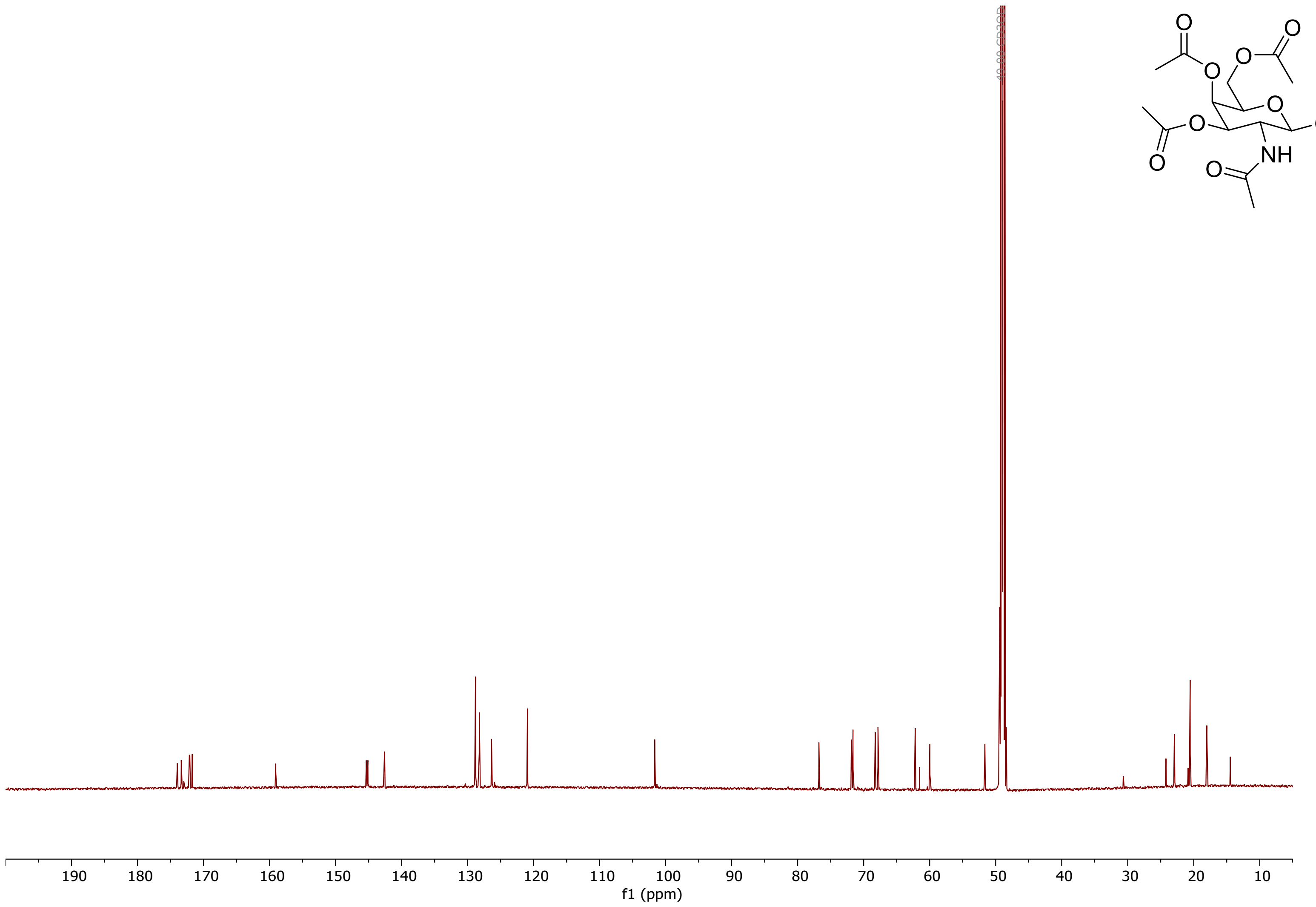
190
180
170
160
150
140
130
120
110
100
90
80
70
60
50
40
30
20
10
f1 (ppm)

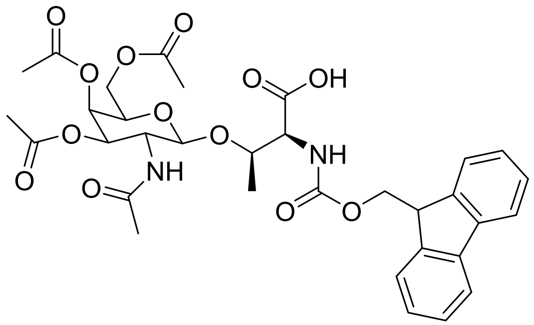
O
O
O
O
OH
O
O
NH
O
NH
O
O
O
O

Fmoc-Ser[GalNAc(Ac)3-α-D]-OH α2

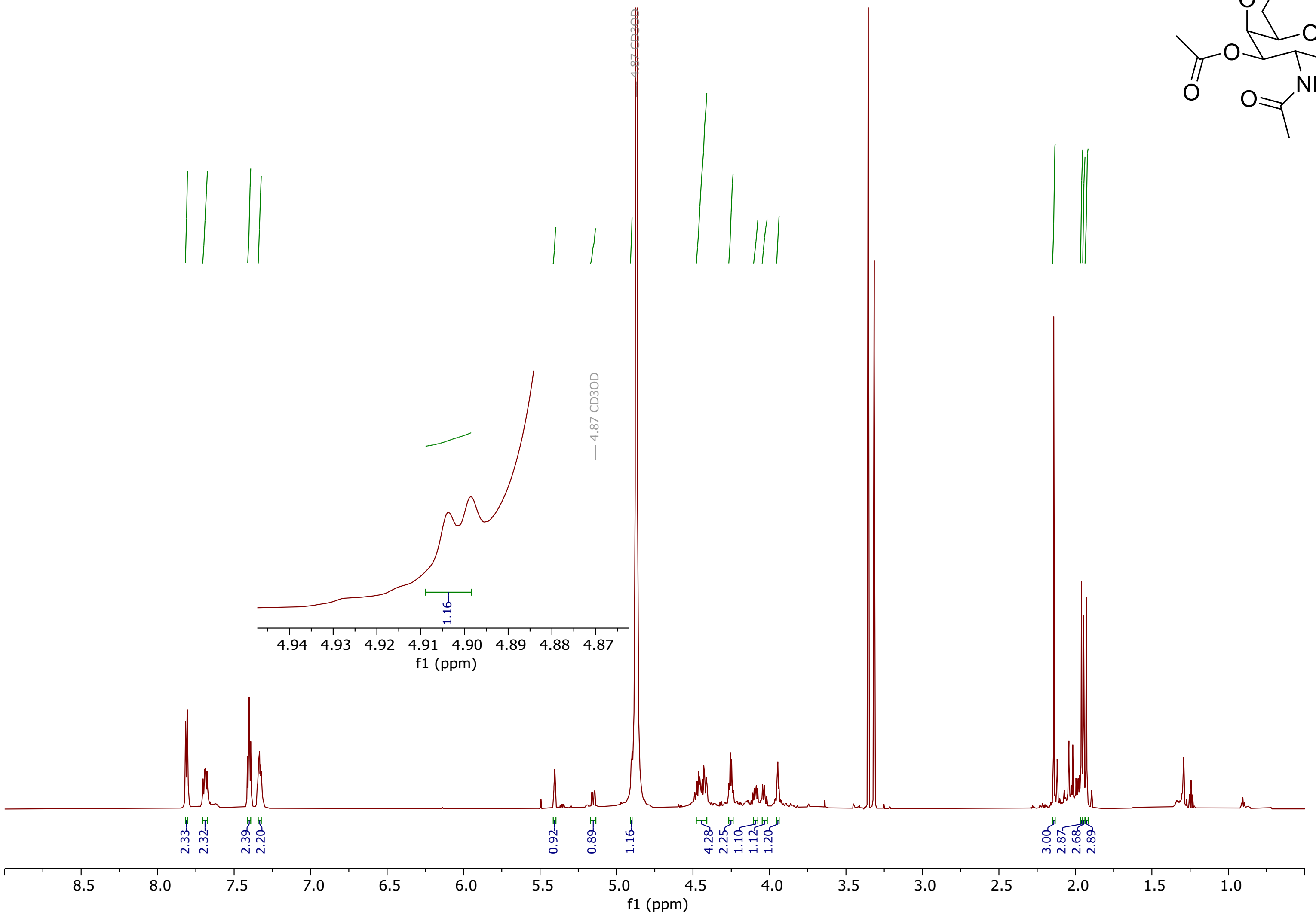

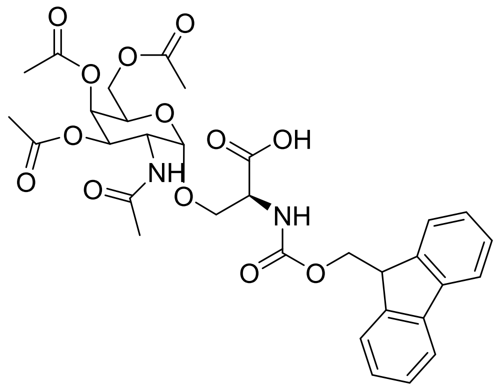

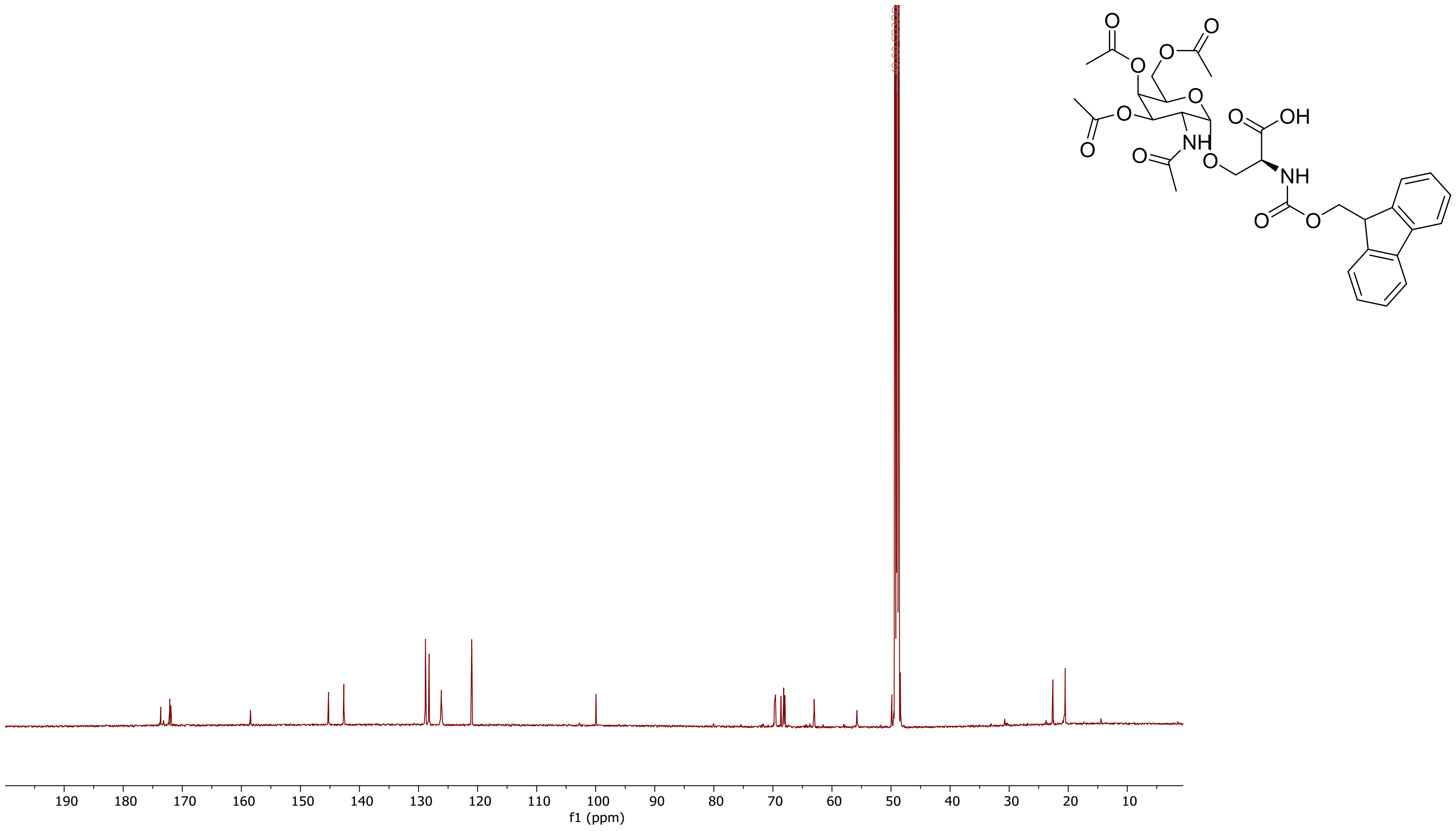
190
180
170
160
150
140
130
120
110
100
90
80
70
60
50
40
30
20
10
f1 (ppm)
O
OH
NH
O
O
NH
O
O
O
O
O
O
O
O
O
O

Fmoc-Ser[GalNAc(Ac)3-β-D]-OH β2

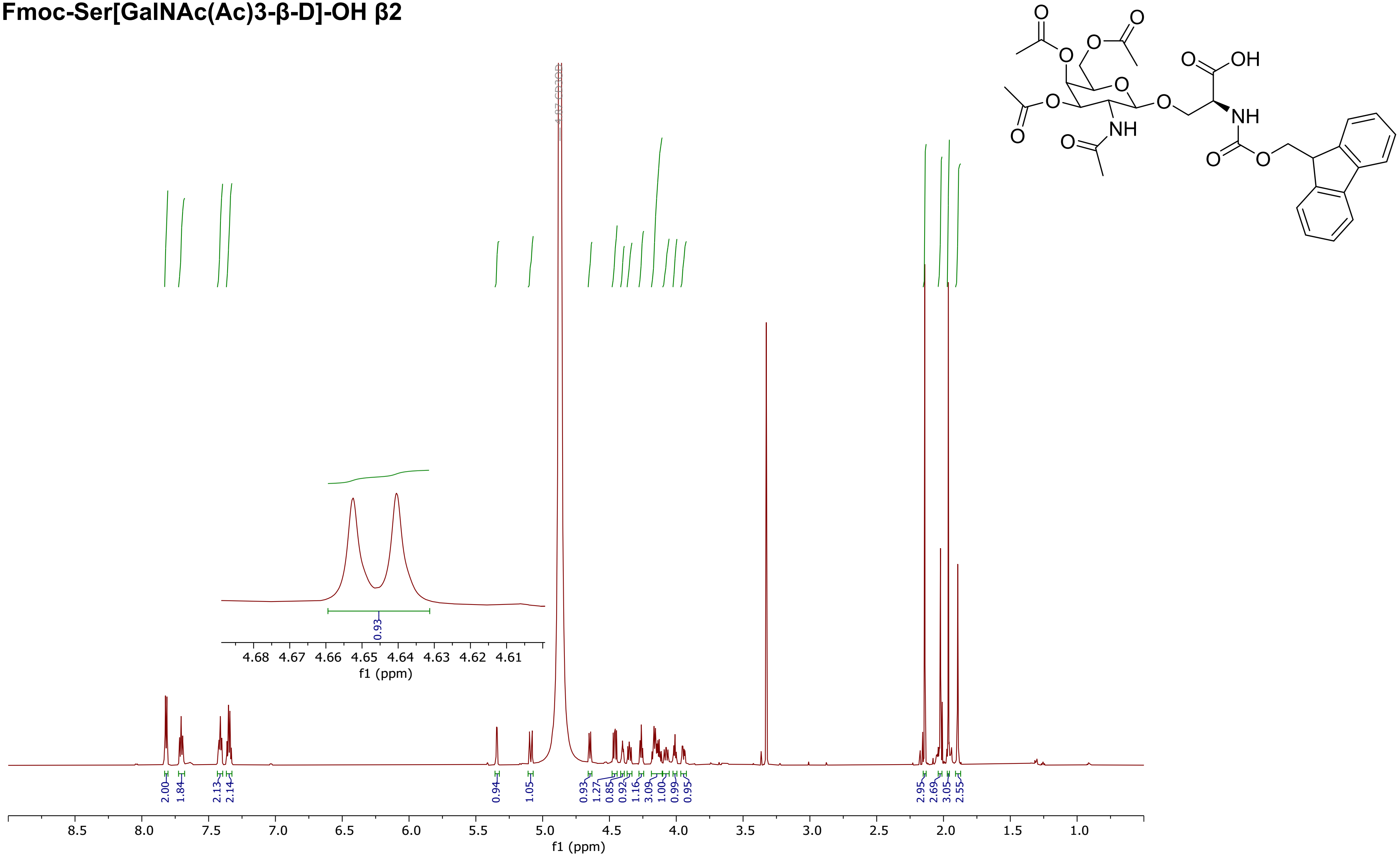

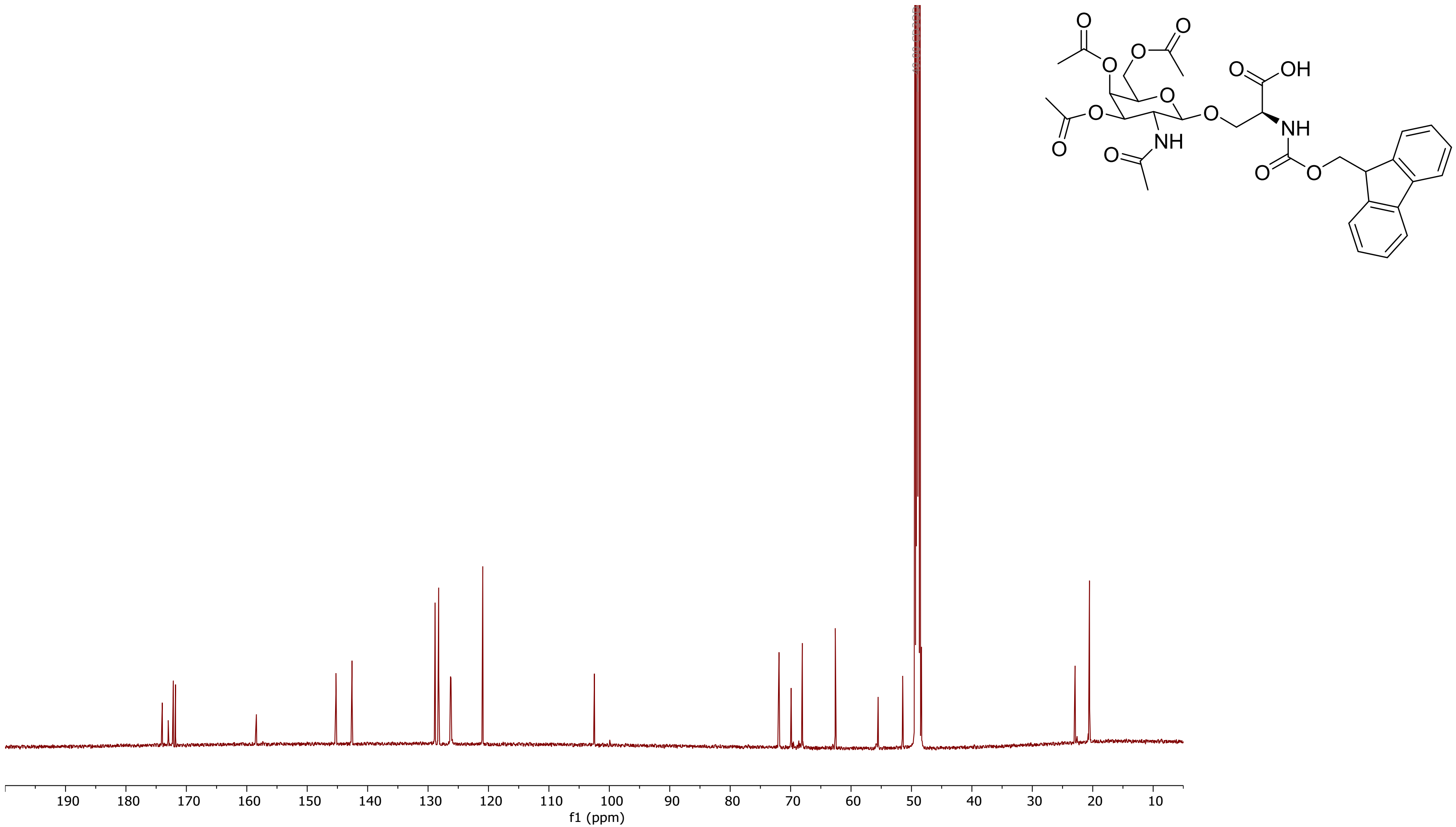
190 180 170 160 150 140 130 120 110 100 90 80 70 60 50 40 30 20 10
f1 (ppm)
O
OH
NH
O

Supplementary references

1 M. Hashimoto, M. Sugiura and S. Terashima, *Tetrahedron*, 2003, **59**, 3063–3087.

2 F. Yu, M. S. McConnell and H. M. Nguyen, *Org Lett*, 2015, **17**, 2018–2021.

3 J. Voorneveld, J. G. M. Rack, L. van Gijlswijk, N. J. Meeuwenoord, Q. Liu, H. S. Overkleeft, G. A. van der Marel, I. Ahel and D. V. Filippov, *Chemistry – A European Journal*, 2021, **27**, 10621–10627.

4 S. Talat, M. Thiruvikraman, S. Kumari and K. J. Kaur, *Glycoconj J*, 2011, **28**, 537–555.

5 E. T. Sletten, S. K. Ramadugu and H. M. Nguyen, *Carbohydr Res*, 2016, **435**, 195–207.

6 B. Yan, W. Li and C. P. R. Hackenberger, *Org Biomol Chem*, 2021, **19**, 8014–8017.
